# Tuna Longline Fishing around West and Central Pacific Seamounts

**DOI:** 10.1371/journal.pone.0014453

**Published:** 2010-12-29

**Authors:** Telmo Morato, Simon D. Hoyle, Valerie Allain, Simon J. Nicol

**Affiliations:** 1 Oceanic Fisheries Programme, Secretariat of the Pacific Community, Noumea, New Caledonia; 2 Departamento de Oceanografia e Pescas, Universidade dos Açores, Horta, Portugal; University of California Davis, United States of America

## Abstract

**Background:**

Seamounts have been identified as aggregating locations for pelagic biodiversity including tuna; however the topography and prevailing oceanography differ between seamounts and not all are important for tuna. Although a relatively common feature in oceanic ecosystems, little information is available that identifies those that are biologically important. Improved knowledge offers opportunities for unique management of these areas, which may advance the sustainable management of oceanic resources. In this study, we evaluate the existence of an association between seamounts and tuna longline fisheries at the ocean basin scale, identify significant seamounts for tuna in the western and central Pacific Ocean, and quantify the seamount contribution to the tuna longline catch.

**Methodology/Principal Findings:**

We use data collected for the Western and Central Pacific Ocean for bigeye, yellowfin, and albacore tuna at the ocean basin scale. GLMs were applied to a coupled dataset of longline fisheries catch and effort, and seamount location information. The analyses show that seamounts may be associated with an annual longline combined catch of 35 thousand tonnes, with higher catch apparent for yellowfin, bigeye, and albacore tuna on 17%, 14%, and 14% of seamounts respectively. In contrast 14%, 18%, and 20% of seamounts had significantly lower catches for yellowfin, bigeye and albacore tuna respectively. Studying catch data in relation to seamount positions presents several challenges such as bias in location of seamounts, or lack of spatial resolution of fisheries data. Whilst we recognize these limitations the criteria used for detecting significant seamounts were conservative and the error in identification is likely to be low albeit unknown.

**Conclusions/Significance:**

Seamounts throughout the study area were found to either enhance or reduce tuna catch. This indicates that management of seamounts is important Pacific-wide, but management approaches must take account of local conditions. Management of tuna and biodiversity resources in the region would benefit from considering such effects.

## Introduction

Seamounts are common topographic features in the world's ocean with the total global area of the seamount biome being recently estimated as 28.8 million km^2^ [1]. Estimates of the number of seamounts taller than approximately 1.5 km occurring worldwide are high and highly variable from about 10 to 14 thousand mapped to over 100 thousand predicted [2]. Seamounts have been identified as hotspots for pelagic biodiversity [3] and some have also been identified as aggregating locations for some tunas (e.g. [4]–[7]). However, their importance for tuna fisheries has not been demonstrated and the contribution of seamounts to global fisheries catch is still poorly estimated [8]–[9].

Tuna is one of the most important world marine fish resources, accounting for nearly 10% of the global marine fisheries catches by landed weight [10] and 20–30% by landed value [11]. Skipjack (*Katsuwonus pelamis*), yellowfin (*Thunnus albacares*), bigeye (*Thunnus obesus*) and albacore (*Thunnus alalunga*) are the species primarily targeted and account for approximately 70% of the global tuna catch [10], [12]. The western and central Pacific Ocean (WCPO) fisheries are the largest tuna fisheries. In 2007, the most recent year with confirmed statistics, the annual catch in the WCPO exceeded 2.4 million tonnes [13], comprised 56% of the total global tuna catch, and was valued at over USD 5 billion dollars. The purse-seine fishery operates predominately between 10°N to 10°S in latitude and accounts for ∼75% of the annual catch. The longline and pole and line fisheries however provide more comprehensive coverage of the region, operating between 35°N to 50°S in latitude and from 120°E to 110°W in longitude and account for ∼10% and ∼7% of the annual catch (i.e. about 64 thousand tonnes of yellowfin tuna, 76 for bigeye and 52 for albacore). In the last decade the pole and line fishery has become more restricted in its operations. The remainder of the annual catch is taken by troll gear and a variety of artisanal gears. Although the longline catch is small in comparison to purse-seine its value is relatively high (30% of the total value). It targets adult bigeye, yellowfin, and albacore tuna, and in some cases sharks or swordfish (*Xiphias gladius*), and operates with fairly standard gear configurations that comprise a main line, branch lines between floats, and float lines.

The data supporting association between tuna and seamounts is only from a few well-studied seamounts in the Azores, north east Atlantic [7] and in the Pacific [14]–[17]. A recent study [3] provided evidence that these observations may hold more generally, but the conclusions were drawn from aggregated data and identification of the number and location of important seamounts for tuna was not possible. Consequently there is only limited information to inform debate upon the value of seamounts for the sustainable management of tuna fisheries.

Here we use data collected for Western and Central Pacific Ocean (WCPO) to address this important knowledge gap. This is the most comprehensive spatial and temporal tuna fisheries and seamount location data available for bigeye, yellowfin, and albacore tuna at the ocean basin scale. We apply generalized linear models (GLMs) to this location-specific fisheries catch data to analyzed catch rate in relation to distance to seamounts to assess the seamount-tuna association at the ocean basin scale, to identify those seamounts that aggregate tuna and then to quantify the contribution to Pacific Ocean tuna catches over time from these seamounts.

## Materials and Methods

### Tuna fisheries and seamount data

The Secretariat of the Pacific Community (SPC) maintains a regional database for tuna catch and effort in the WCPO that dates back to 1958. The resolution of the data varies from precise location data to aggregated data at coarser resolution. This database has been extensively used for research and monitoring purposes such as assessing the state of exploitation of the tuna stocks. From this dataset all longline sets (n = 1.8 million) from the period 1960–2007 and for the area 50°N-50°S and 105°E-95°W were extracted (Figure S1). Purse seine and pole-and-line information were not used in the study due to limited spatial coverage. Catch by species was returned as numbers, estimated weight, and discarded fish. Date and set location at an approximately 0.1 degrees resolution, number of hooks, flag, and fleet of the fishing boat were also extracted. Data for the high-seas areas are commonly reported at 5 by 5 degree squares. These data points were excluded from the analyses reducing the number of longline sets available for the high-seas and thus increasing the uncertainty for these areas.

The term seamount has been defined many times but there is no “generally accepted” definition. Instead, most definitions serve the particular needs of a discipline. In this study we adopt a general and broad definition of seamounts as any topographically distinct seafloor feature that is at least 100 m height but which does not break the sea surface. The numbers and locations of Pacific seamounts have been determined by Kitchingman and Lai [18] and updated in Kitchingman et al. [19]. This dataset was later validated for the WCPO by Allain et al. [20] by cross checking its seamount positions with other datasets available for the Pacific region. The cross-checking method validated the Kitchingman and Lai features that were confirmed by at least one of the other datasets derived from ship sounding. When the feature was only confirmed by satellite-derived datasets, the Kitchingman and Lai feature could not be considered as ‘validated’, but was noted as ‘cross-checked’. Seamounts not listed in Kitchingman and Lai but occurring in another dataset were added to the database after screening and cross-checking with bathymetric maps and other datasets. Geographically aggregated potential seamounts were examined separately. They were plotted on top of the best resolution bathymetric map available for the area of interest (i.e., multibeam maps) to confirm if they represented several spatially close seamounts or a single large feature misidentified as several seamounts. Decision criteria were based on visual interpretation of the bathymetric map that was trusted over the automatic extraction. Redundant records or duplicates were removed from the database. The spatial location of a seamount was assumed to be at the center of the feature. This process was able to remove atolls and islands that had been incorrectly classified as seamounts. The resulting seamount database included 7741 features ([18], [20], Dataset S1).

The distance (*d*) of each longline set to the closest seamount was estimated using the simple spherical law of cosines. Only sets located within 100 km from any seamount summit were selected (n = 1.05 million sets), to allow computing and because longline sets beyond 100 km are unlikely to be under the influence of the seamount itself which was estimated at 20–40 km [3], [7]. Of the 7741 seamounts in the dataset, only 4465 had longline sets within 100 km of their summits. Information on the physical characteristics of most seamounts, such as depth of the summit, elevation and slope are unknown or not accurately measured preventing any detailed analyses on the parameters that may be driving tuna aggregations.

### Data analyses and modeling

To quantify the interaction between tuna fisheries in the Pacific Ocean and seamounts we undertook the data modeling in two parts. Firstly, we evaluated whether the ocean-basin scale patterns of association with seamounts detected for tuna in the Pacific Ocean through the analysis of at-sea observer data [3] were repeated in the more comprehensive tuna longline logbook data, and secondly we modeled individual seamounts to identify those where aggregating effects were evident. We then quantified the catch attributable to seamounts from these identified seamounts.

### Ocean-basin scale model

We used GLM techniques to standardize logbook catch data [21], [22] for albacore, bigeye and yellowfin tuna (n = 1.05 million sets). The explanatory variables included in the model were year from 1960 to 2007 as a proxy for temporal variability, moon phase divided into 8 categories from New to Full as the relationship between lunar periodicity and catch rates has been demonstrated for a wide variety of commercially exploited species (e.g. [23]), area of 5 by 5 degrees latitude and longitude, fleet type categorized by the country in which each vessel is registered (flag) and fleet type (i.e. domestic, locally-based offshore, and distant-water), fishing effort measured as the number of hooks in each set, and distance to seamount. The species being targeted, and the depth and time of a set can influence the catch. Information on these variables was not contained within the database and fleet type and number of hooks was used as a proxy measures for these variables. The volume of data was beyond the computing capacity available to fit one single model to the data. To resolve this issue the model was fitted independently to the data in each nineteen geographical regions of 20 by 20 degrees latitude and longitude.

The Akaike's Information Criterion (AIC) was used to test for effects of distance to seamount by modeling the data with and without the distance to seamount term. The model used for each geographical area was:

Model 1: Ln(Catch_n_ +1) ∼ Year + moon phase +5×5Latlong area + Flag_fleet + LN(effort)

Model2: Model1 + distSM

A Gaussian family of error distributions with identity link function was used. We examined the residuals to check that the assumptions were not violated for each model. Tuna species were considered to be affected by seamounts if the distance to seamount term improved the model (i.e. if the ΔAIC between the two models was negative). For these models, negative estimates (i.e. slope) for the distance to seamount parameter indicated higher catch rates, and positive estimates lower catch rates closer to summits.

### Detecting significant seamounts

To identify seamounts with significantly different catch rates close to their summits when compared to further away from the summit we restricted the data set to only the seamounts with more than 100 longline sets within 100 km from their summits. This restriction resulted in 1345 seamounts within Exclusive Economic Zones (EEZs) and 313 seamounts in the high seas being evaluated. The identification of important seamounts was expected to be influenced by the sample size of longline sets and to account for this potential uncertainty we conducted the same analyses increasing the minimum sample size to n>1000 longline sets within 100 km of their summit for comparison. The two models used for each seamount were the same as for the ocean basin analyses. The AIC was used to test for effects of distance to seamount. Seamounts were considered to have an aggregation effect if the seamount distance effect was significant (negative ΔAIC) and if the distance to seamount estimated parameter was negative (i.e. higher catch rate when closer to seamount summits), while positive estimates indicated no aggregation effect and lower catch rates closer to seamount summits. The model was run for each species separately.

### Quantifying tuna seamounts catch

After selecting seamounts that significantly increased the catch rate we quantified the proportion of the longline catch reported in the catch and effort database that was caught within 100 km of their summits. These proportions were then applied to the total longline catch of tuna species in WCPO [13] to estimate the longline catch around WCPO seamounts.

## Results

### Ocean-basin scale patterns

Pseudo-R2 values for all GLMs averaged 0.45 with yellowfin ranging between 0.24 and 0.48; bigeye between 0.21 and 0.60; albacore between 0.21 and 0.83 (Table S1). Thus the explained variance of the GLM fitted to each area was consistent with those typically fitted for standardization of tuna catch data [24]. There was some support for including the explanatory variable “distance to seamount” at the ocean basin scale, but the level of support varied among the 19 areas modeled (Table S1). Significant seamount aggregating effects were detected in 32% of the areas for yellowfin, 16% for bigeye, and 11% for albacore. Significantly lower catch rates at the seamount summit was detected in 11% of areas for yellowfin, 16% for bigeye, and 37% for albacore. No effect was detected in 10 (53%) of the areas for bigeye and yellowfin and 9 areas (47%) for albacore.

### Detecting individual seamounts with significant associations of tuna

**Figure 1:**
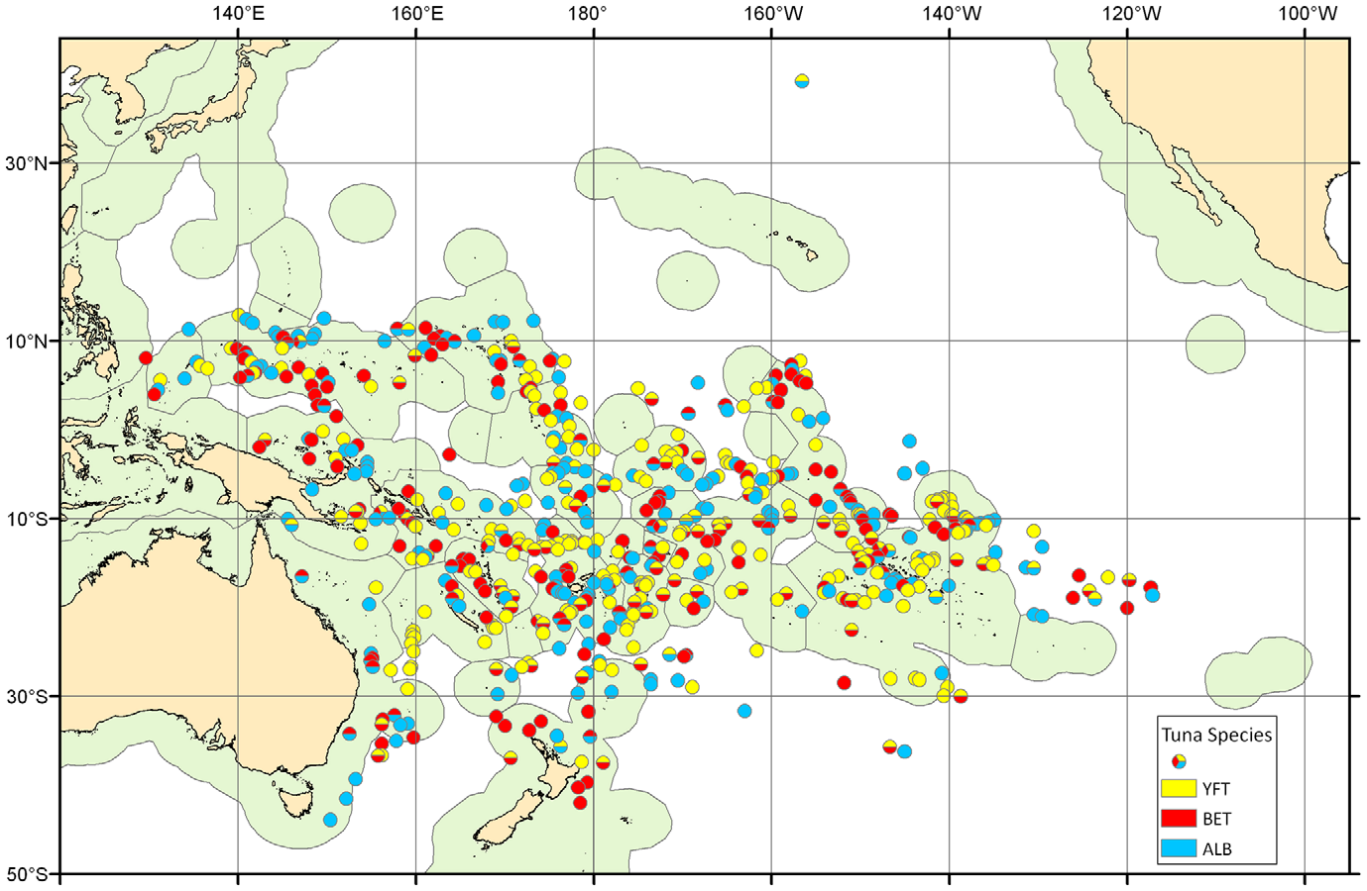
Location of seamounts with significant higher catch rates of tuna. Significant seamounts were detected by Akaike's Information Criterion on modeling the catch data with and without the distance to seamount term. YFT is yellowfin tuna (yellow), BET is bigeye (red), and ALB is albacore (blue).

There was model support for significantly higher catch rates of tuna close to seamount summits when compared to further away for any species of tuna on 602 seamounts, representing 36% of all screened seamount (where n>100 longline sets was applied; Table 1). From the 1658 seamounts screened, 283 showed higher catch rates for yellowfin (17.1% of seamounts, Figure S2), 233 for bigeye (14.1%, Figure S3) and 230 for albacore (13.9%, Figure S4). A further 41% of seamounts screened have significantly lower tuna catch rates close to their summits. Lower catches were detected on 232 (14.0%), 303 (18.3%), 336 (20.3%) seamounts for yellowfin, bigeye and albacore respectively. Seamounts with significantly higher tuna catch rates for all three species were found throughout the study area (Figure 1). More significant seamounts were located within EEZs (n = 510) than on the high seas (n = 107), but these values match the number of screened seamounts since only 19% of the screened seamounts were located on the high seas.

**Table 1:** Number of seamounts showing significant increase in tuna catch rates.

	Significant Seamounts			
	n>100		n>1000	
	No.	%	No.	%
Screened	1658		212	
Unique	602	36	97	46
YFT	283	17.1	40	18.9
BET	233	14.1	49	23.1
ALB	230	13.9	40	18.9
YFT-BET-ALB	9	0.5	2	0.9
YFT-BET	80	4.8	15	7.1
YFT-ALB	24	1.4	5	2.4
ALB-BET	49	3.0	14	6.6

No. is the number of seamounts, % is the percentage of screened seamounts showing significantly increase in tuna catch rates as detected by Akaike's Information Criterion on modeling the catch data with and without the distance to seamount term. n is the number of longline sets performed within 100 km from any seamount summit; YFT is yellowfin tuna, BET is bigeye and ALB is albacore.

The EEZs showing larger numbers of significant seamounts (n>30) were French Polynesia, Fiji, Federated States of Micronesia and the Line Islands in Kiribati, and Solomon Islands. There were 144 seamounts that showed higher catch rates for more than 1 species with 9 seamounts showing higher catches for all three species, 80 for bigeye - yellowfin, 49 for bigeye – albacore, and 24 for yellowfin-albacore. A detailed table with the information on the significant seamounts by EEZ is presented as supplementary information (Table S2). The number of seamounts identified as important was reduced to 97 when the minimum criteria for screening was set to N>1000 sets on the seamount, but the percentages were comparable (Table 1).

### Quantifying tuna longline catch around seamounts

**Figure 2:**
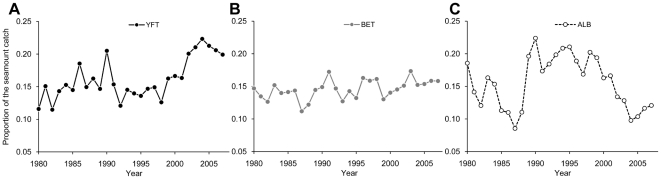
Longline tuna catch around Western Central Pacific Ocean significant seamounts as proportion of region's catch. A) YFT is yellowfin tuna, B) BET is bigeye and C) ALB is albacore.

The proportion of tuna longline catch in association with significant seamounts varied over time (Figure 2). For yellowfin tuna, the proportion of the catch around significant yellowfin seamounts varied from about 12% of the catch reported at the operational level in the 1980's to over 20% in recent years. For bigeye tuna, the proportion of the catch taken near significant bigeye seamounts was more constant while for albacore, the proportion increased from 1980's to the 1990, and decreased to about 10% in recent years.

**Figure 3:**
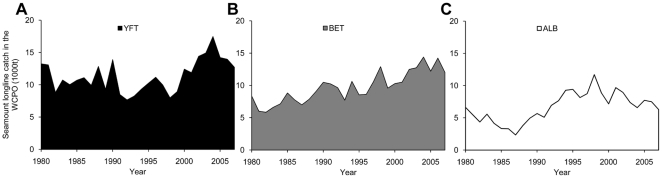
Longline tuna catch around Western Central Pacific Ocean significant seamounts as cumulative catch (thousands tonnes). A) YFT is yellowfin tuna, B) BET is bigeye and C) ALB is albacore.

Estimates of annual landings for each species were generated from the tuna longline catch associated with seamounts (Figure 3). Approximately 15 thousand tonnes per year of yellowfin, 12 thousand tonnes per year of bigeye and 7.5 thousand tonnes per year of albacore were caught around significant seamounts in recent years. These catches have increased over time for bigeye and albacore but have been stable for yellowfin. For recent years, significant seamounts in the western central Pacific region may be associated with an annual catch by longline of as much as 35 thousand tonnes per year or ∼16% of the longline catch.

The estimated catches for each significant seamount and species are shown in Supplementary Figures S5, S6, S7. Seamounts showing higher catches of yellowfin (Figure S5) and bigeye (Figure S6) for the whole period 1980–2007 were located between the parallels 10°N and 10°S with Federated States of Micronesia, Papua New Guinea, Solomon Islands and Kiribati (Phoenix Islands and Line Islands) having important seamounts. For albacore, the most productive seamounts were located south of the parallel 10°S, in Australia, New Zealand, New Caledonia, Fiji (Figure S7). Estimated tuna catch for each EEZ is presented in Table S3.

## Discussion

Our analyses suggest that higher catch rates of tuna by the longline fleet are associated with a significant number of seamounts throughout the Pacific Ocean. This study concluded that about 36%–46% of the screened seamounts in the west and central Pacific show significant higher catch rate values for at least one tuna species. Our study estimated that seamounts that significantly increased tuna catch rates may be responsible for up to 16% of the annual longline catch, i.e. about 35 thousand tonnes. These estimates are high considering that many seamounts in the region are very deep [20] and thus unlikely to aggregate pelagic visitors [7], and that many seamounts were not included in the study due to insufficient fisheries logbook data. In contrast with previous studies [8], the methodology applied was very conservative with only those seamounts that showed a significant effect in increasing fishing catches used in the calculations. Furthermore, the complexity of the western Pacific Ocean basin, with many islands, atolls and ridges, and the existence of numerous fishing aggregating devices (FAD) in the region may also divert tuna from gathering around specific seamounts. It should be noted that this study covered only 1658 of the 7741 seamounts that have been inferred in the region [18], [20], and although we cannot generalize to the other seamounts these numbers may be underestimated.

Aggregations of yellowfin, bigeye or albacore in the Pacific have been previously described for only a few seamounts, such as the Hawaiian Cross [15] and Emperor seamounts [4], the Espiritu Santo seamount in Baja California, Mexico [16], and the Capricorn seamount in Tonga [25]. The Emperor Seamount chain (Hawaii), for example, has a long history of tuna fishing around its features. The Japanese fleet has been longlining for albacore since 1938 and fishing with pole-and-line since 1973 [4]. Pole-and-line catches in this seamount chain represented 5 to 25% of the total albacore landings by Japanese vessels. Cross seamount, also in Hawaii waters, is another well known seamount in the Pacific that has become a handline and deep longline fishing ground for bigeye and yellowfin tuna in the 1990's [26], [27]. The handline fishery on the Cross Seamount was based on high catch rates of juvenile fish. In the western and central Pacific Ocean there are fewer reported examples of tuna fishing around seamounts. The exception is the longline fishing experiments in Tongan waters where catch rates were found to be much higher close to Capricorn seamount when compared to the open ocean [25]. During these experiments catch per unit of effort on Capricorn were 12.7 tuna per hundred hooks (mainly bigeye and yellowfin) while open ocean sets averaged 1.9 tuna per hundred hooks (mainly albacore). Our study identified many seamounts throughout the western Pacific Ocean that may act as important aggregating points for tuna species. The main challenge in the future will be to understand what factors are driving tuna aggregations on specific seamounts. We believe that incorporating detailed oceanographic data along with better seamount morphological data will unveil many of the seamount and tuna ecology paradigms.

Purse seine and pole-and-line information were not used in the study due to limited spatial coverage but a similar study including these fishing methods should be made when better coverage is available. Such study would be especially useful for skipjack in addition to yellowfin and bigeye. Studying catch data in relation to seamount positions presents several major challenges. The first is common to any large scale study on seamounts and lies in deciding what seamounts are and where they are located. Mislocation of seamounts may occur in the databases which may increase the uncertainty of the forthcoming analyses. The seamount database used in this analysis has been extensively screened to remove incorrectly identified features and/or locations [20] and whilst the potential for error remains this has been minimized. The second lies in the spatial resolution of the fisheries data for the specific purpose of quantifying seamount-associated catches. For example, the position of a longline set represents only a rough approximation of where the gear is actually fishing since one set can be more than 100 km long and the logsheet will contain only one lat/long position. Our analyses are also likely to include type 1 and type 2 errors, with non-significant seamounts estimated to be significant or the other way around. Our model assumes that all sets are independent, but this assumption was violated to some extent. For example, the effort of individual vessels, which tend to have different catch rates, may be spatially aggregated. Whilst we recognize these limitations in the methods the criteria used for detecting significant seamounts was conservative and the error in identification is likely to be low albeit unknown.

It is likely that these issues influence the ability to detect the effect of seamounts at the ocean basin scale and might explain why more equivocal results than positive or negative effects were observed in the analyses conducted. The low volume of location specific data for the high seas areas available for this study is also likely to have contributed and revisiting this analysis with the inclusion of this data would be beneficial. Inclusion of a seamount depth term in the GLM would also be beneficial as seamounts whose topography is less favorable could be excluded but mainly for identifying ideal seamount depths conducive for aggregation of pelagic species. The trend in the analyses however was consistent with that reported from observer data in the Pacific Ocean with a positive effect on catch rates detected for yellowfin, negative effects for albacore and equivocal results for bigeye [3]. The ocean basin scale analysis was also hindered by computing limitations with a requirement to analyse the data in sub-blocks.

Seamounts enhancing tuna catch were found throughout the study area, with many lying within national EEZs. This aspect may facilitate management measures, since it is easier to implement effective management within national boundaries [28]–[30]. We observed that some seamounts have positive effects on catch for more than one species in most EEZs and these seamounts in particular might be priorities for management. Few seamounts on the high seas had enough data to screen, and many of these seamounts may also enhance tuna catch. Seamounts on the high seas with higher catch were located in the pocket between the Solomon Islands and Tuvalu, and in the high seas area south of Cook Islands. A more detailed analysis is required to evaluate the importance of these areas for tuna resources.

The higher concentrations of tuna in some predictable locations indicate that tuna are vulnerable to concentrated fishing effort, since as abundances drop, such as the present situation of yellowfin and bigeye [31], [32] fishing vessels may concentrate on areas where fish remain. This has important management implications since such aggregation areas may promote hyperstability of catch rates, and raise concerns about range contraction and concentration during stock declines [33]–[35]. Similarly our detection of seamounts with significantly lower catches indicates that management approaches must take account of local conditions. The influence of seamounts should be carefully accounted and future abundance estimations should consider spatial along with temporal variation in abundance [36].

## Supporting Information

Figure S1 Location of the 1.8 million longline sets (blue dots) recorded in the SPC's Catch and effort database (1960–2007). Location of seamounts (black stars) included in the present study (n = 1658) and EEZs boundaries (grey lines) are also shown.
(2.35 MB TIF)

Figure S2 Location of seamounts with higher catch rates of yellowfin tuna (YFT). Seamounts detected by Akaike's Information Criterion on modeling the data with and without the distance to seamount term.
(0.96 MB TIF)

Figure S3 Location of seamounts with higher catch rates of bigeye tuna (BET). Seamounts detected by Akaike's Information Criterion on modeling the data with and without the distance to seamount term.
(0.95 MB TIF)

Figure S4 Location of seamounts with higher catch rates of albacore (ALB). Seamounts detected by Akaike's Information Criterion on modeling the data with and without the distance to seamount term.
(0.94 MB TIF)

Figure S5 Estimated seamount catches (tons) for the whole period (1965–2007) for yellowfin tuna.
(0.99 MB TIF)

Figure S6 Estimated seamount catches (tons) for the whole period (1965–2007) for bigeye tuna.
(0.98 MB TIF)

Figure S7 Estimated seamount catches (tons) for the whole period (1965–2007) for albacore.
(0.97 MB TIF)

Table S1 Summary statistics for the GLM used to standardized yellowfin (YFT), bigeye (BET) and albacore (ALB) catch data for longline sets (N) performed within 100 km from any seamount summit. For each model we present the effect of including the term for distance to seamount on the Akaike's Information Criterion (ΔAIC), the parameter estimate for the relationship with distance-to-seamount, and whether the effect represents a significantly higher or lower catch rate close to seamounts summits (SM).
(0.12 MB DOC)

Table S2 Summary statistics for the GLM used to identify seamounts with significantly higher catch rates close to their summits, restricted to seamounts with more than 100 longline sets (N) within 100 km from their summits. Models were run for each individual seamount. For each model we present the effect of including the term for distance to seamount on the Akaike's Information Criterion (ΔAIC), the parameter estimate for the relationship with distance-to-seamount.
(1.15 MB DOC)

Table S3 Estimated seamounts catch of tuna for different EEZ in the Pacific Ocean. Prop. of EEZ catch is the proportion of the tuna catch in that EEZ allocated to seamounts. Catch values are in tons.
(0.52 MB DOC)

Dataset S1 Dataset containing the seamounts mapped in the Pacific Ocean from 50°N to 50°S [18]–[20]. Longitude, latitude, depth and elevation may not be accurate since in most cases it was measured from global datasets with low resolution.
(1.12 MB XLSX)
