# Supplementary figures and images for: Tuna Longline Fishing around West and Central Pacific Seamounts
Source: PLoS One. 2010 Dec 29;5(12):e14453. doi: 10.1371/journal.pone.0014453 (PMC3012065; doi:10.1371/journal.pone.0014453)

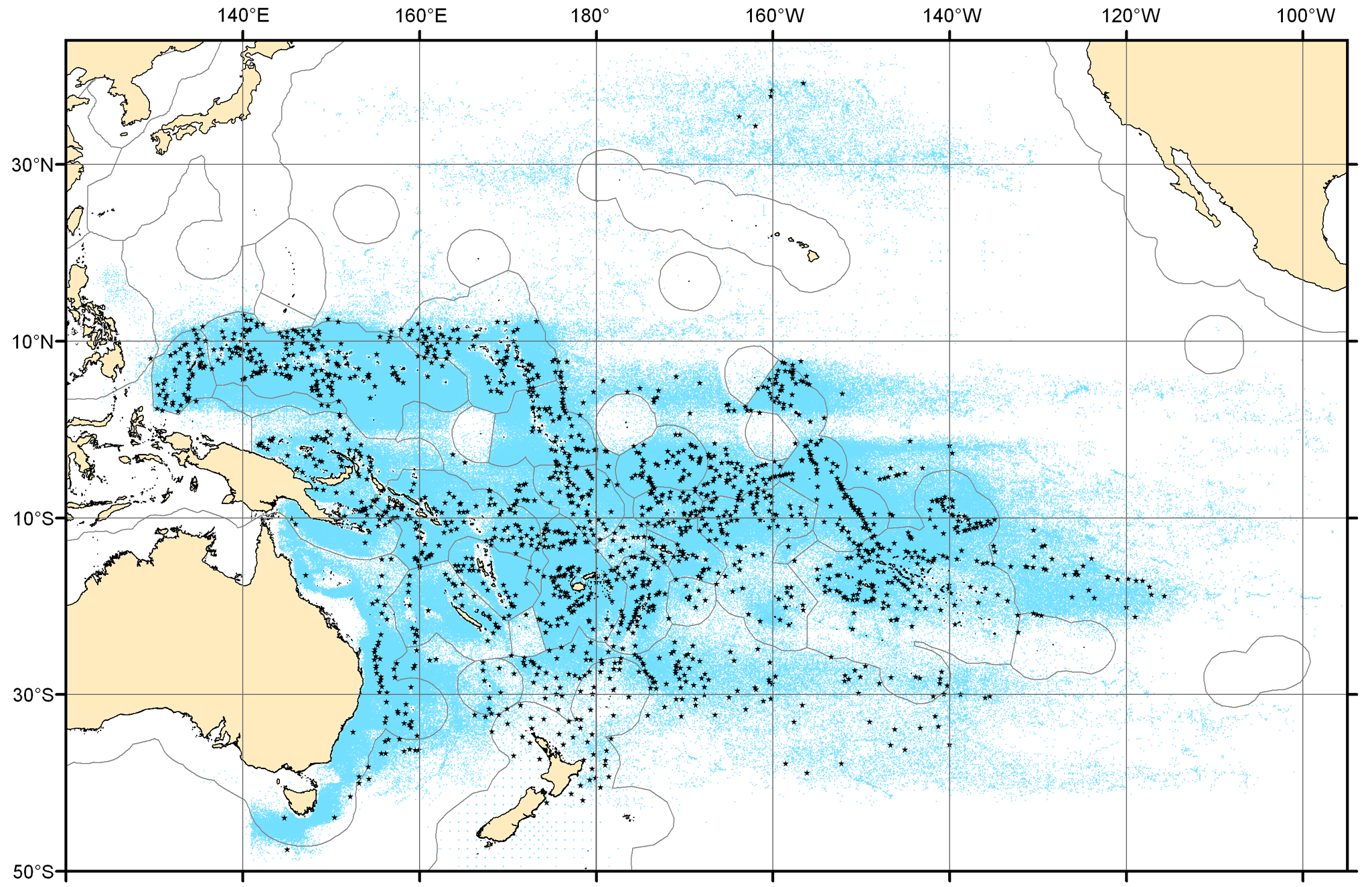

Supplement: Figure S1 — Location of the 1.8 million longline sets (blue dots) recorded in the SPC's Catch and effort database (1960–2007). Location of seamounts (black stars) included in the present study (n = 1658) and EEZs boundaries (grey lines) are also shown. (2.35 MB TIF) [file pone.0014453.s001.tif]

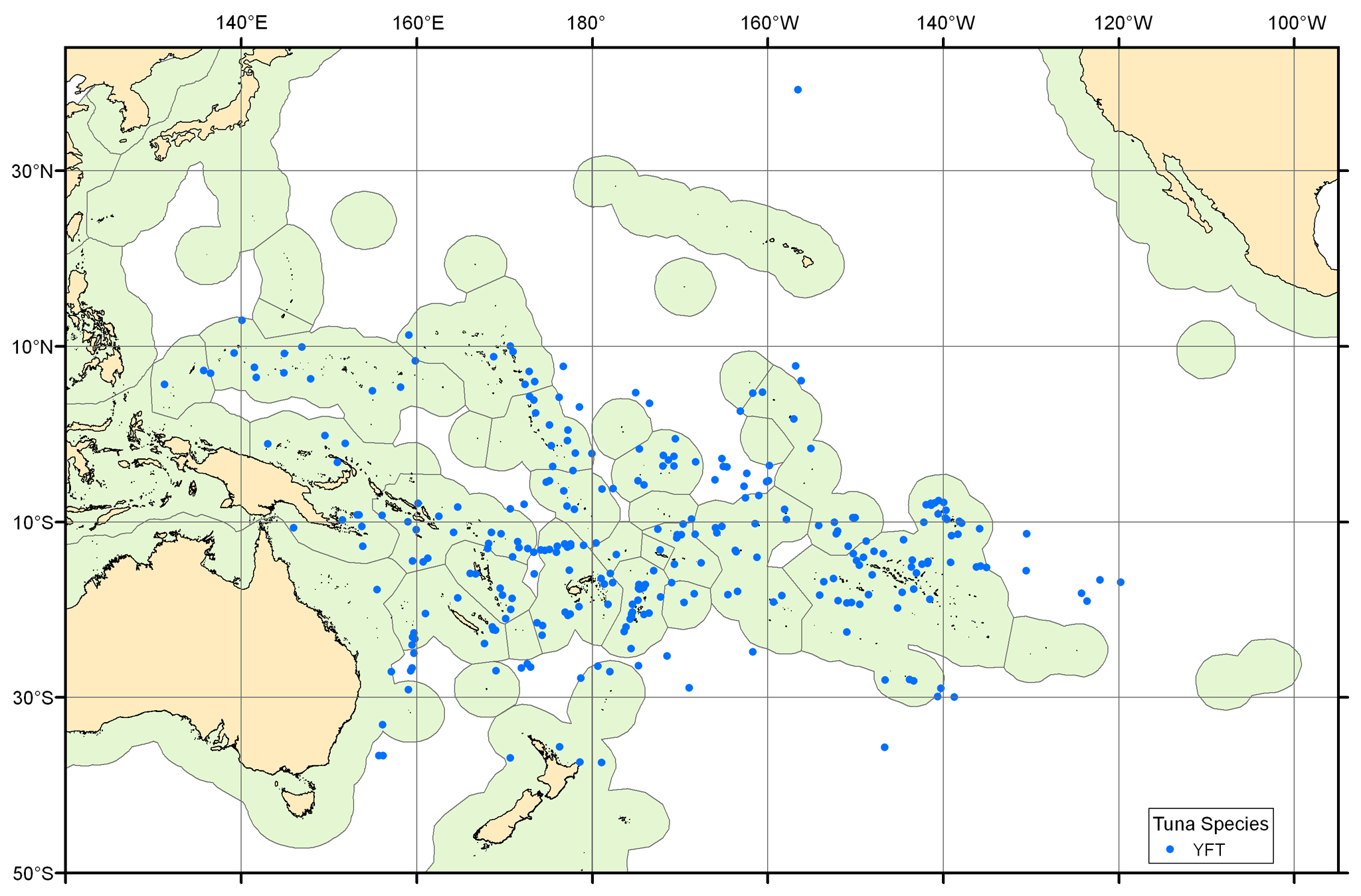

Supplement: Figure S2 — Location of seamounts with higher catch rates of yellowfin tuna (YFT). Seamounts detected by Akaike's Information Criterion on modeling the data with and without the distance to seamount term. (0.96 MB TIF) [file pone.0014453.s002.tif]

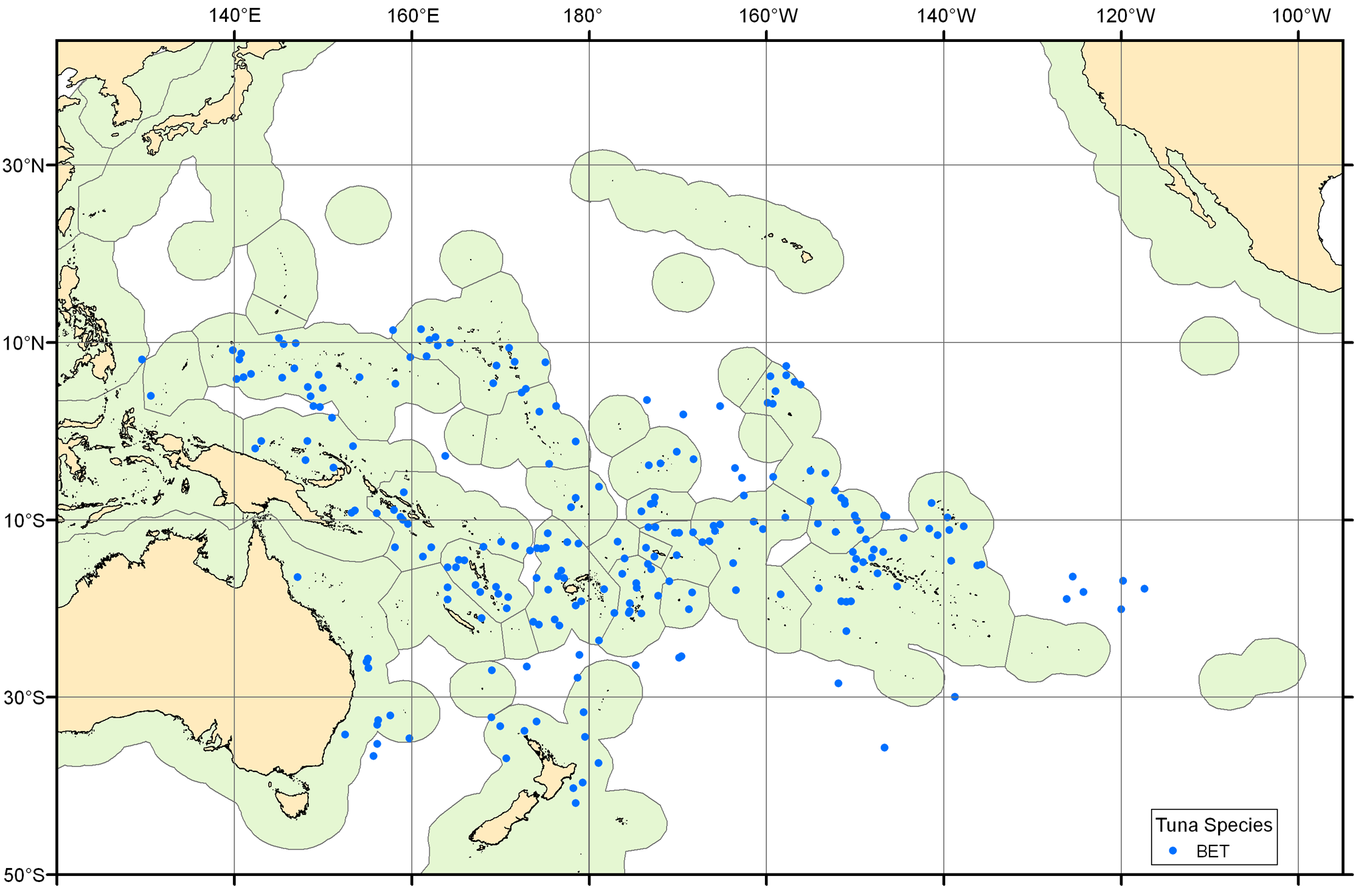

Supplement: Figure S3 — Location of seamounts with higher catch rates of bigeye tuna (BET). Seamounts detected by Akaike's Information Criterion on modeling the data with and without the distance to seamount term. (0.95 MB TIF) [file pone.0014453.s003.tif]

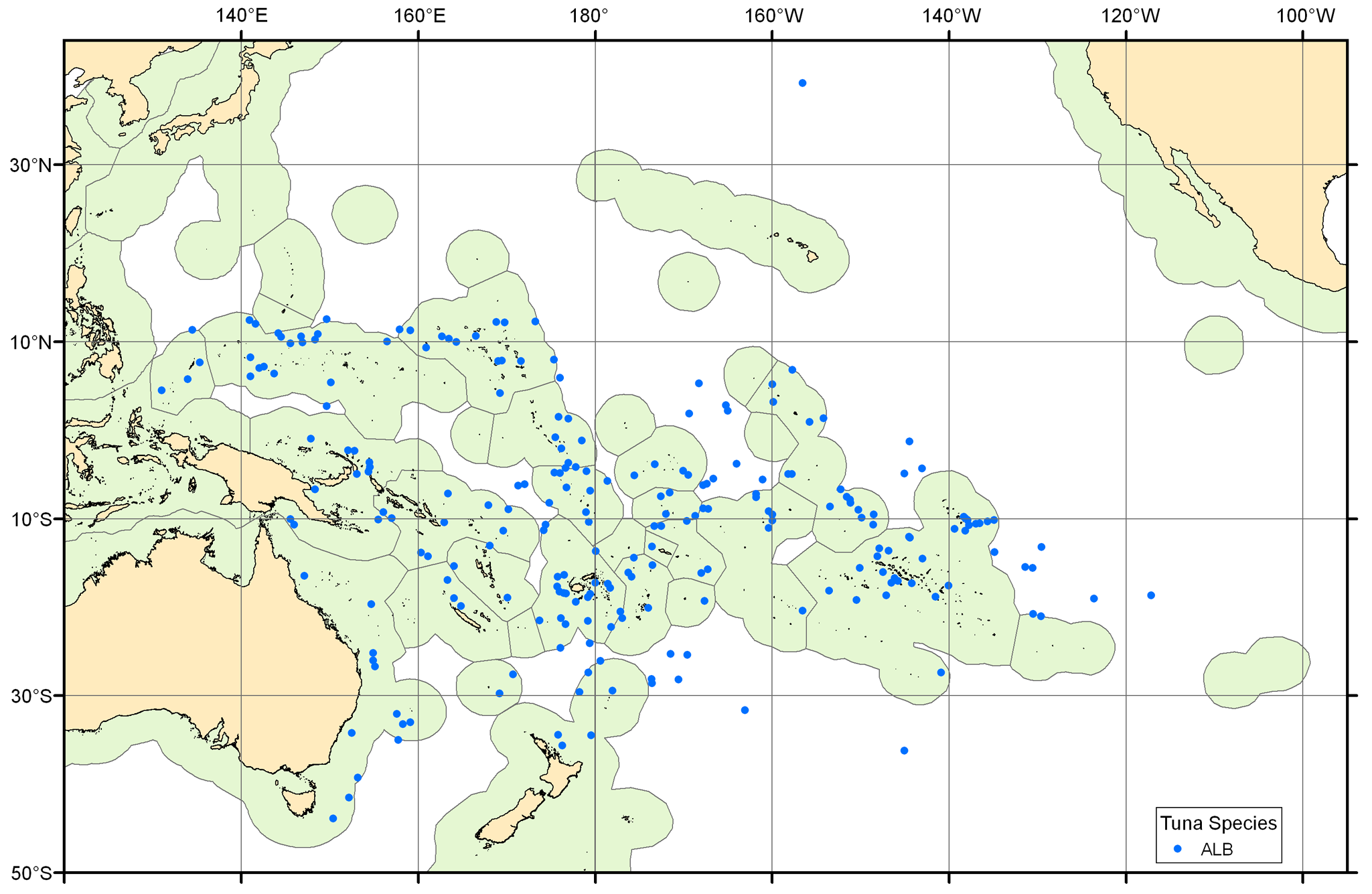

Supplement: Figure S4 — Location of seamounts with higher catch rates of albacore (ALB). Seamounts detected by Akaike's Information Criterion on modeling the data with and without the distance to seamount term. (0.94 MB TIF) [file pone.0014453.s004.tif]

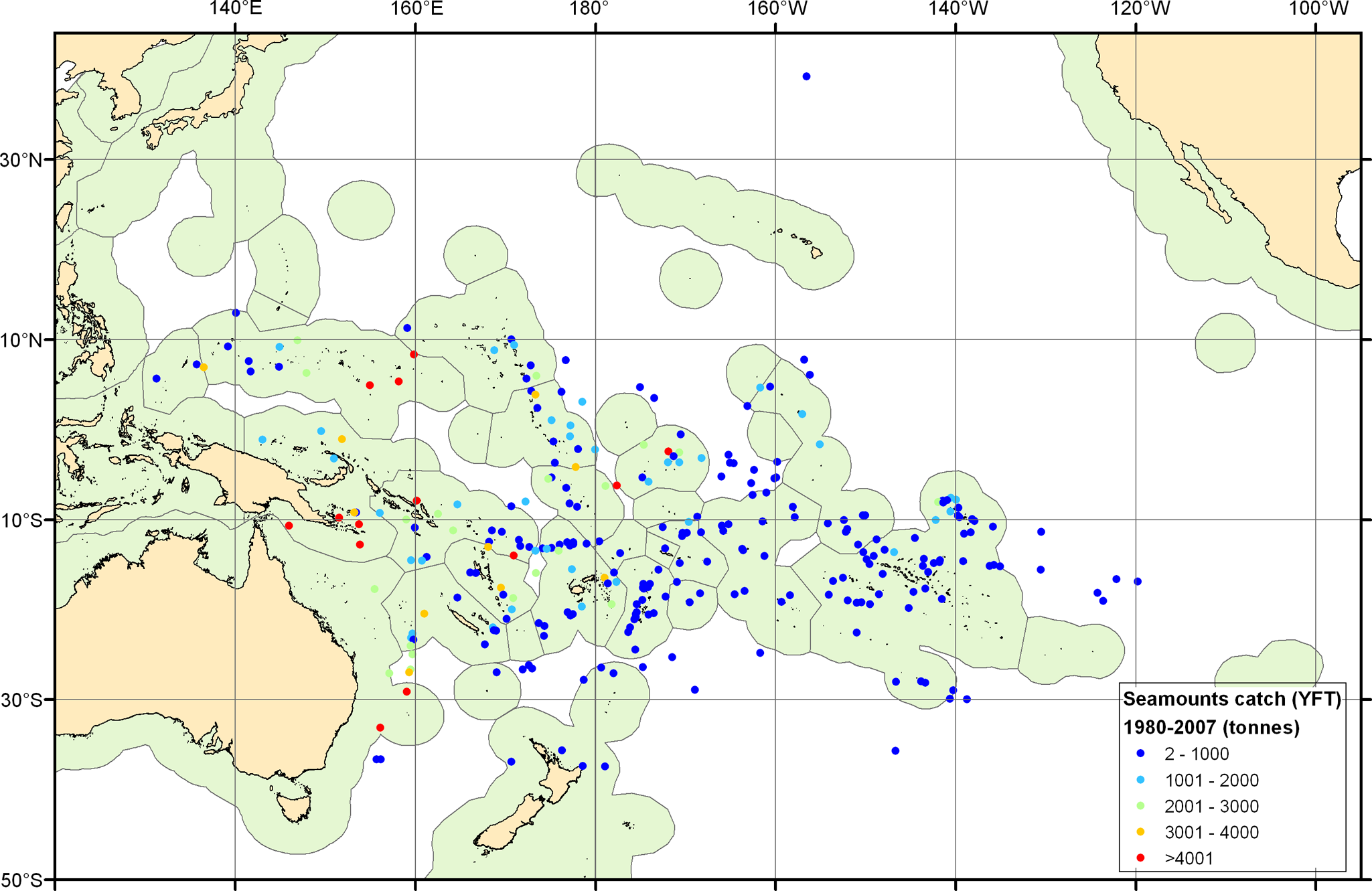

Supplement: Figure S5 — Estimated seamount catches (tons) for the whole period (1965–2007) for yellowfin tuna. (0.99 MB TIF) [file pone.0014453.s005.tif]

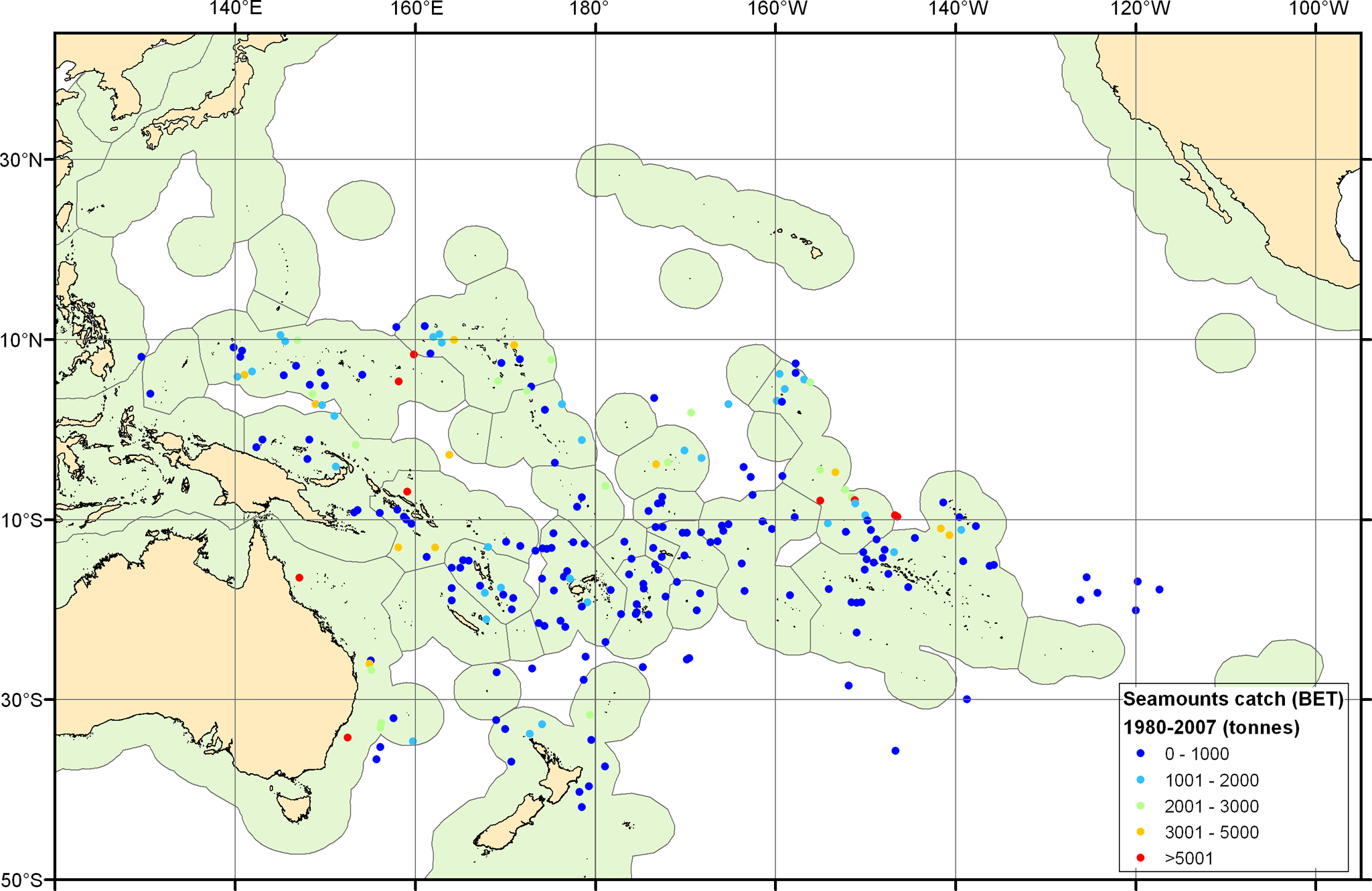

Supplement: Figure S6 — Estimated seamount catches (tons) for the whole period (1965–2007) for bigeye tuna. (0.98 MB TIF) [file pone.0014453.s006.tif]

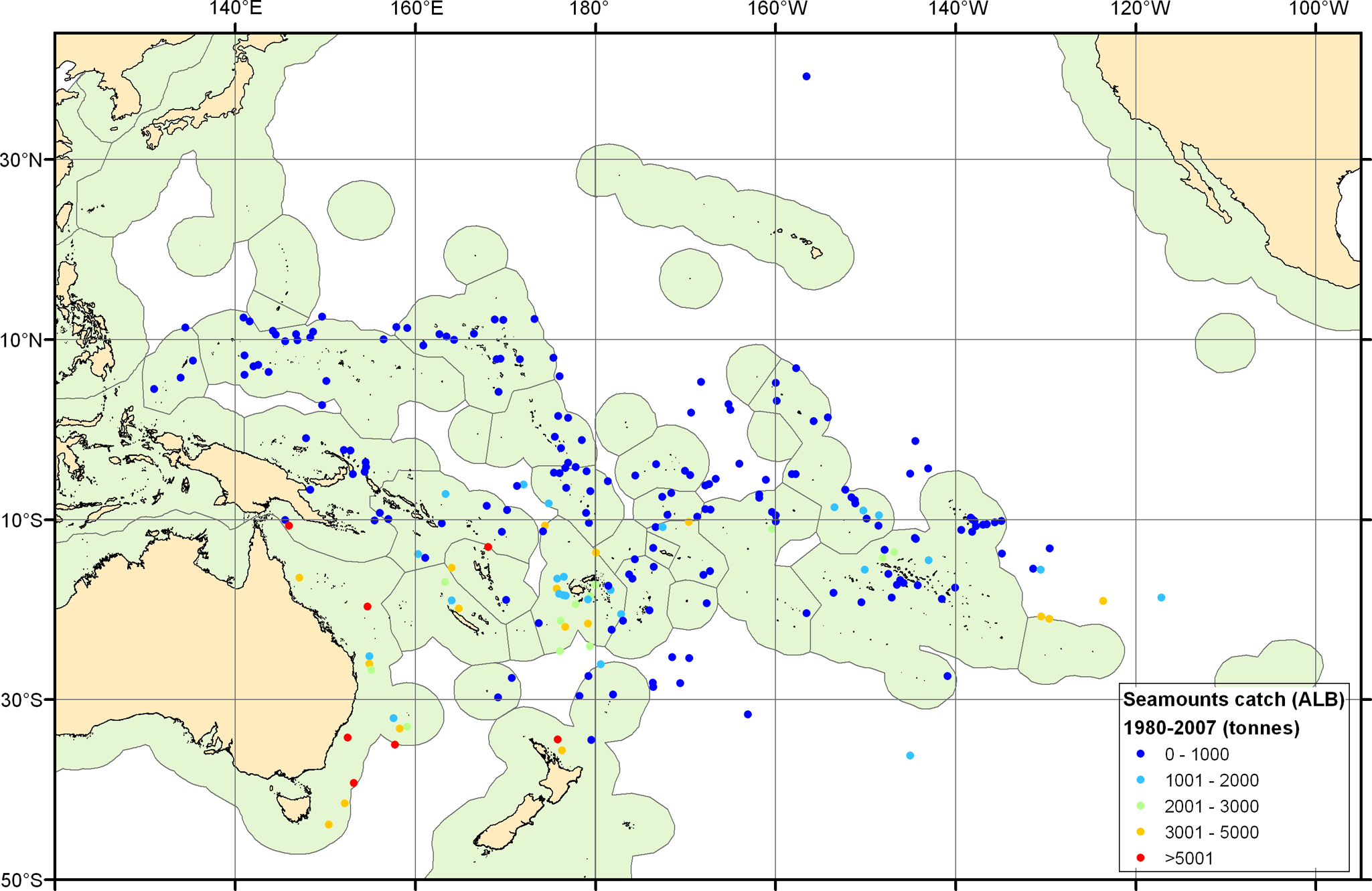

Supplement: Figure S7 — Estimated seamount catches (tons) for the whole period (1965–2007) for albacore. (0.97 MB TIF) [file pone.0014453.s007.tif]
