# Supplementary material for: Tuna Longline Fishing around West and Central Pacific Seamounts
Source: PLoS One. 2010 Dec 29;5(12):e14453. doi: 10.1371/journal.pone.0014453 (PMC3012065; doi:10.1371/journal.pone.0014453)
Supplement: Table S1 — Summary statistics for the GLM used to standardized yellowfin (YFT), bigeye (BET) and albacore (ALB) catch data for longline sets (N) performed within 100 km from any seamount summit. For each model we present the effect of including the term for distance to seamount on the Akaike's Information Criterion (ΔAIC), the parameter estimate for the relationship with distance-to-seamount, and whether the effect represents a significantly higher or lower catch rate close to seamounts summits (SM). (0.12 MB DOC) [file pone.0014453.s008.doc]

**Table S1*.*** Summary statistics for the GLM used to standardized yellowfin (YFT), bigeye (BET) and albacore (ALB) catch data for longline sets (N) performed within 100km from any seamount summit. Models were run for areas of 20 by 20 degrees. For each model we present the effect of including the term for distance to seamount on the Akaike's Information Criterion (ΔAIC), the parameter estimate for the relationship with distance-to-seamount, and whether the effect represents a significantly higher or lower catch rate close to seamounts summits (SM).

| **Species** | **Lat.** | **Lon.** | **Estimate**  **distSM** | **std error** | **t value** | **p(t>0)** | **ΔAIC** | **N** | **Pseudo-*R*^2^** | **SM effect** |
| --- | --- | --- | --- | --- | --- | --- | --- | --- | --- | --- |
| YFT | -40 | 140 | 0.00280 | 0.00013 | 22.212 | 0.000 | -491.006 | 77015 | 0.36 | lower |
| YFT | -40 | 160 | -0.00041 | 0.00013 | -3.090 | 0.002 | -7.591 | 106868 | 0.48 | higher |
| YFT | -40 | 180 | -0.00036 | 0.00018 | -1.984 | 0.047 | -1.980 | 39116 | 0.34 | higher |
| YFT | -40 | 200 | -0.00021 | 0.00026 | -0.819 | 0.413 | 1.317 | 14519 | 0.33 |  |
| YFT | -40 | 220 | -0.00044 | 0.00042 | -1.057 | 0.290 | 0.830 | 5478 | 0.30 |  |
| YFT | -40 | 240 | -0.00100 | 0.00090 | -1.106 | 0.269 | 0.532 | 822 | 0.36 |  |
| YFT | -20 | 140 | -0.00003 | 0.00017 | -0.197 | 0.844 | 1.961 | 58261 | 0.48 |  |
| YFT | -20 | 160 | 0.00011 | 0.00011 | 0.986 | 0.324 | 1.026 | 145227 | 0.41 |  |
| YFT | -20 | 180 | -0.00082 | 0.00011 | -7.452 | 0.000 | -53.720 | 148021 | 0.40 | higher |
| YFT | -20 | 200 | -0.00066 | 0.00011 | -5.950 | 0.000 | -33.520 | 120683 | 0.36 | higher |
| YFT | -20 | 220 | -0.00159 | 0.00023 | -6.820 | 0.000 | -44.830 | 22648 | 0.44 | higher |
| YFT | -20 | 240 | 0.00072 | 0.00061 | 1.180 | 0.238 | 0.535 | 2411 | 0.45 |  |
| YFT | 0 | 140 | 0.00017 | 0.00012 | 1.505 | 0.132 | -0.271 | 121217 | 0.44 | lower |
| YFT | 0 | 160 | 0.00008 | 0.00016 | 0.496 | 0.620 | 1.753 | 66633 | 0.40 |  |
| YFT | 0 | 180 | -0.00022 | 0.00043 | -0.500 | 0.617 | 1.740 | 9833 | 0.38 |  |
| YFT | 0 | 200 | 0.00010 | 0.00034 | 0.281 | 0.779 | 1.920 | 16642 | 0.24 |  |
| YFT | 20 | 160 | -0.00017 | 0.00081 | -0.207 | 0.836 | 1.949 | 1187 | 0.30 |  |
| YFT | 20 | 180 | -0.00080 | 0.00043 | -1.850 | 0.064 | -1.780 | 2803 | 0.43 | higher |
| YFT | 20 | 200 | 0.00087 | 0.00048 | 1.824 | 0.068 | -1.510 | 2332 | 0.28 | lower |
|  |  |  |  |  |  |  |  |  |  |  |
| **Species** | **Lat.** | **Lon.** | **Estimate**  **distSM** | **std error** | **t value** | **p(t>0)** | **ΔAIC** | **N** | **Pseudo-*R*^2^** | **SM effect** |
| BET | -40 | 140 | 0.00038 | 0.00011 | 3.548 | 0.000 | -10.538 | 77015 | 0.33 | lower |
| BET | -40 | 160 | 0.00025 | 0.00013 | 1.857 | 0.063 | -1.464 | 106868 | 0.37 | lower |
| BET | -40 | 180 | 0.00043 | 0.00017 | 2.571 | 0.010 | -4.684 | 39116 | 0.25 | lower |
| BET | -40 | 200 | -0.00058 | 0.00024 | -2.404 | 0.016 | -3.880 | 14519 | 0.21 | higher |
| BET | -40 | 220 | -0.00072 | 0.00040 | -1.795 | 0.073 | -1.373 | 5478 | 0.42 | higher |
| BET | -40 | 240 | 0.00017 | 0.00114 | 0.149 | 0.882 | 1.973 | 822 | 0.58 |  |
| BET | -20 | 140 | 0.00014 | 0.00016 | 0.888 | 0.375 | 1.209 | 58261 | 0.36 |  |
| BET | -20 | 160 | -0.00012 | 0.00009 | -1.278 | 0.201 | 0.361 | 145227 | 0.43 |  |
| BET | -20 | 180 | -0.00012 | 0.00010 | -1.293 | 0.196 | 0.323 | 148021 | 0.52 |  |
| BET | -20 | 200 | 0.00002 | 0.00010 | 0.247 | 0.805 | 1.939 | 120683 | 0.56 |  |
| BET | -20 | 220 | -0.00031 | 0.00022 | -1.425 | 0.154 | -0.045 | 22648 | 0.51 | higher |
| BET | -20 | 240 | 0.00078 | 0.00069 | 1.132 | 0.258 | 0.651 | 2411 | 0.42 |  |
| BET | 0 | 140 | 0.00016 | 0.00010 | 1.624 | 0.104 | -0.646 | 121217 | 0.32 | lower |
| BET | 0 | 160 | 0.00068 | 0.00013 | 5.152 | 0.000 | -24.689 | 66633 | 0.36 | lower |
| BET | 0 | 180 | -0.00049 | 0.00038 | -1.291 | 0.197 | 0.273 | 9833 | 0.28 |  |
| BET | 0 | 200 | 0.00022 | 0.00029 | 0.758 | 0.448 | 1.417 | 16642 | 0.21 |  |
| BET | 20 | 160 | -0.00009 | 0.00117 | -0.073 | 0.942 | 1.994 | 1187 | 0.45 |  |
| BET | 20 | 180 | 0.00160 | 0.00066 | 2.410 | 0.016 | -4.412 | 2803 | 0.47 | lower |
| BET | 20 | 200 | 0.00030 | 0.00076 | 0.395 | 0.693 | 1.835 | 2332 | 0.60 |  |

| **Species** | **Lat.** | **Lon.** | **Estimate**  **distSM** | **std error** | **t value** | **p(t>0)** | **ΔAIC** | **N** | **Pseudo-*R*^2^** | **SM effect** |
| --- | --- | --- | --- | --- | --- | --- | --- | --- | --- | --- |
| ALB | -40 | 140 | 0.00042 | 0.00014 | 3.040 | 0.002 | -7.124 | 77015 | 0.63 | lower |
| ALB | -40 | 160 | 0.00095 | 0.00021 | 4.616 | 0.000 | -19.404 | 106868 | 0.45 | lower |
| ALB | -40 | 180 | 0.00079 | 0.00018 | 4.390 | 0.000 | -17.488 | 39116 | 0.64 | lower |
| ALB | -40 | 200 | 0.00063 | 0.00023 | 2.717 | 0.007 | -5.513 | 14519 | 0.80 | lower |
| ALB | -40 | 220 | -0.00008 | 0.00034 | -0.226 | 0.821 | 1.946 | 5478 | 0.55 |  |
| ALB | -40 | 240 | 0.00030 | 0.00125 | 0.240 | 0.810 | 1.931 | 822 | 0.65 |  |
| ALB | -20 | 140 | 0.00026 | 0.00017 | 1.509 | 0.131 | -0.286 | 58261 | 0.62 | lower |
| ALB | -20 | 160 | 0.00039 | 0.00011 | 3.664 | 0.000 | -11.463 | 145227 | 0.56 | lower |
| ALB | -20 | 180 | 0.00045 | 0.00010 | 4.393 | 0.000 | -17.365 | 148021 | 0.61 | lower |
| ALB | -20 | 200 | 0.00067 | 0.00011 | 6.250 | 0.000 | -37.185 | 120683 | 0.66 | lower |
| ALB | -20 | 220 | -0.00037 | 0.00023 | -1.655 | 0.098 | -0.760 | 22648 | 0.70 | higher |
| ALB | -20 | 240 | -0.00005 | 0.00054 | -0.090 | 0.928 | 1.991 | 2411 | 0.83 |  |
| ALB | 0 | 140 | -0.00005 | 0.00004 | -1.070 | 0.285 | 0.851 | 121217 | 0.21 |  |
| ALB | 0 | 160 | -0.00015 | 0.00008 | -2.022 | 0.043 | -2.112 | 66633 | 0.40 | higher |
| ALB | 0 | 180 | 0.00017 | 0.00026 | 0.683 | 0.495 | 1.517 | 9833 | 0.48 |  |
| ALB | 0 | 200 | -0.00005 | 0.00014 | -0.381 | 0.703 | 1.853 | 16642 | 0.26 |  |
| ALB | 20 | 160 | -0.00057 | 0.00096 | -0.593 | 0.553 | 1.580 | 1187 | 0.59 |  |
| ALB | 20 | 180 | -0.00079 | 0.00070 | -1.138 | 0.255 | 0.568 | 2803 | 0.52 |  |
| ALB | 20 | 200 | 0.00004 | 0.00070 | 0.052 | 0.958 | 1.997 | 2332 | 0.51 |  |
