# Supplementary material for: Tuna Longline Fishing around West and Central Pacific Seamounts
Source: PLoS One. 2010 Dec 29;5(12):e14453. doi: 10.1371/journal.pone.0014453 (PMC3012065; doi:10.1371/journal.pone.0014453)
Supplement: Table S2 — Summary statistics for the GLM used to identify seamounts with significantly higher catch rates close to their summits, restricted to seamounts with more than 100 longline sets (N) within 100 km from their summits. Models were run for each individual seamount. For each model we present the effect of including the term for distance to seamount on the Akaike's Information Criterion (ΔAIC), the parameter estimate for the relationship with distance-to-seamount. (1.15 MB DOC) [file pone.0014453.s009.doc]

**Table S2**. Summary statistics for the GLM used to identify seamounts with significantly higher catch rates close to their summits, restricted to seamounts with more than 100 longline sets (N) within 100 km from their summits. Models were run for each individual seamount. For each model we present the effect of including the term for distance to seamount on the Akaike's Information Criterion (ΔAIC), the parameter estimate for the relationship with distance-to-seamount.

| **Species** | **SM Code** | **Seamount name** | **EEZ / High seas** | **Lon.** | **Latitude** | **Estimate**  **distSM** | **Std error** | **t value** | **P value** | ΔAIC | **N** |
| --- | --- | --- | --- | --- | --- | --- | --- | --- | --- | --- | --- |
| ALB | 5655 |  | American Samoa | 190.383 | -10.295 | -0.0021 | 0.0011 | -1.8591 | 0.0630 | -1.487 | 1233 |
| YFT | 5655 |  | American Samoa | 190.383 | -10.295 | -0.0032 | 0.0014 | -2.2299 | 0.0258 | -3.017 | 1233 |
| ALB | 5733 |  | American Samoa | 186.683 | -10.875 | -0.0038 | 0.0014 | -2.7774 | 0.0055 | -5.782 | 588 |
| BET | 5733 |  | American Samoa | 186.683 | -10.875 | -0.0030 | 0.0013 | -2.2011 | 0.0278 | -2.888 | 588 |
| ALB | 5734 |  | American Samoa | 187.483 | -10.908 | -0.0016 | 0.0010 | -1.6131 | 0.1068 | -0.626 | 863 |
| BET | 5734 |  | American Samoa | 187.483 | -10.908 | -0.0056 | 0.0009 | -6.0230 | 0.0000 | -34.549 | 863 |
| YFT | 5734 |  | American Samoa | 187.483 | -10.908 | -0.0048 | 0.0012 | -4.0697 | 0.0000 | -14.703 | 863 |
| BET | 5819 |  | American Samoa | 190.150 | -11.504 | -0.0027 | 0.0009 | -3.1328 | 0.0017 | -7.885 | 1170 |
| YFT | 5819 |  | American Samoa | 190.150 | -11.504 | -0.0028 | 0.0010 | -2.8415 | 0.0045 | -6.132 | 1170 |
| BET | 5822 |  | American Samoa | 189.700 | -11.521 | -0.0095 | 0.0057 | -1.6669 | 0.0956 | -0.800 | 500 |
| YFT | 5822 |  | American Samoa | 189.700 | -11.521 | -0.0147 | 0.0065 | -2.2702 | 0.0232 | -3.192 | 500 |
| YFT | 5851 |  | American Samoa | 189.617 | -11.868 | -0.0018 | 0.0010 | -1.8273 | 0.0677 | -1.363 | 1287 |
| BET | 6128 | Muli | American Samoa | 189.917 | -14.055 | -0.0031 | 0.0019 | -1.6099 | 0.1075 | -0.613 | 185 |
| YFT | 6246 | Papatua | American Samoa | 189.357 | -14.888 | -0.0036 | 0.0011 | -3.4040 | 0.0007 | -9.688 | 1410 |
| ALB | 6344 |  | American Samoa | 192.717 | -15.743 | -0.0033 | 0.0015 | -2.1473 | 0.0319 | -2.744 | 338 |
| ALB | 6410 |  | American Samoa | 191.983 | -16.207 | -0.0037 | 0.0017 | -2.1233 | 0.0338 | -2.638 | 226 |
| YFT | 9223 |  | American Samoa | 192.445 | -14.712 | -0.0029 | 0.0020 | -1.4266 | 0.1538 | -0.080 | 331 |
| YFT | 6619 |  | Australia | 155.500 | -17.783 | -0.0032 | 0.0020 | -1.5896 | 0.1123 | -0.672 | 360 |
| ALB | 6802 |  | Australia | 154.717 | -19.700 | -0.0060 | 0.0014 | -4.1473 | 0.0000 | -15.682 | 645 |
| ALB | 7346 | Moreton South | Australia | 154.939 | -25.200 | -0.0040 | 0.0006 | -6.5939 | 0.0000 | -41.539 | 2536 |
| BET | 7436 | Brisbane North | Australia | 155.089 | -25.707 | -0.0027 | 0.0018 | -1.5054 | 0.1322 | -0.142 | 921 |
| ALB | 7489 | Moreton / Brisbane South | Australia | 154.916 | -26.041 | -0.0048 | 0.0004 | -10.8527 | 0.0000 | -115.805 | 5630 |
| BET | 7489 | Moreton / Brisbane South | Australia | 154.916 | -26.041 | -0.0038 | 0.0004 | -10.1351 | 0.0000 | -100.824 | 5630 |
| ALB | 7591 |  | Australia | 155.133 | -26.733 | -0.0016 | 0.0007 | -2.1863 | 0.0288 | -2.555 | 2233 |
| BET | 7591 |  | Australia | 155.133 | -26.733 | -0.0011 | 0.0006 | -1.7620 | 0.0781 | -1.224 | 2233 |
| YFT | 7636 |  | Australia | 157.133 | -27.100 | -0.0018 | 0.0005 | -3.5492 | 0.0004 | -10.643 | 3411 |
| YFT | 7790 |  | Australia | 159.067 | -29.183 | -0.0021 | 0.0006 | -3.4061 | 0.0007 | -9.699 | 2722 |
| ALB | 7951 |  | Australia | 157.583 | -32.117 | -0.0030 | 0.0020 | -1.4633 | 0.1434 | -0.159 | 466 |
| BET | 7951 |  | Australia | 157.583 | -32.117 | -0.0032 | 0.0017 | -1.8385 | 0.0660 | -1.408 | 466 |
| BET | 7991 | Barcoo | Australia | 156.251 | -32.610 | -0.0020 | 0.0009 | -2.2415 | 0.0250 | -3.072 | 1146 |
| ALB | 8038 |  | Australia | 159.109 | -33.068 | -0.0043 | 0.0015 | -2.9051 | 0.0037 | -6.544 | 503 |
| BET | 8041 | Taupo | Australia | 156.150 | -33.167 | -0.0029 | 0.0007 | -4.0947 | 0.0000 | -14.914 | 1893 |
| YFT | 8041 | Taupo | Australia | 156.150 | -33.167 | -0.0023 | 0.0008 | -2.8977 | 0.0038 | -6.476 | 1893 |
| ALB | 8124 |  | Australia | 152.517 | -34.233 | -0.0013 | 0.0007 | -1.7703 | 0.0767 | -1.161 | 4914 |
| BET | 8124 |  | Australia | 152.517 | -34.233 | -0.0018 | 0.0004 | -4.2108 | 0.0000 | -15.855 | 4914 |
| BET | 8145 | Flinders | Australia | 159.733 | -34.667 | -0.0015 | 0.0010 | -1.4710 | 0.1414 | -0.205 | 877 |
| ALB | 8425 |  | Australia | 153.200 | -39.317 | -0.0045 | 0.0012 | -3.9278 | 0.0001 | -13.828 | 735 |
| ALB | 8509 |  | Australia | 152.183 | -41.533 | -0.0178 | 0.0036 | -4.9836 | 0.0000 | -24.796 | 138 |
| ALB | 8561 | Cascade | Australia | 150.380 | -43.920 | -0.0031 | 0.0009 | -3.6352 | 0.0003 | -11.341 | 2040 |
| ALB | 8712 | Cairns | Australia | 147.167 | -16.517 | -0.0013 | 0.0004 | -3.0537 | 0.0023 | -7.364 | 7903 |
| BET | 8712 | Cairns | Australia | 147.167 | -16.517 | -0.0006 | 0.0004 | -1.4692 | 0.1418 | -0.169 | 7903 |
| ALB | 9308 |  | Australia | 158.278 | -33.233 | -0.0039 | 0.0011 | -3.6252 | 0.0003 | -11.258 | 943 |
| YFT | 5318 |  | Cook Island | 198.983 | -7.067 | -0.0059 | 0.0037 | -1.6045 | 0.1087 | -0.622 | 167 |
| ALB | 5333 |  | Cook Island | 198.217 | -7.233 | -0.0078 | 0.0052 | -1.4913 | 0.1359 | -0.263 | 210 |
| BET | 5342 |  | Cook Island | 197.483 | -7.333 | -0.0057 | 0.0024 | -2.3124 | 0.0208 | -3.439 | 348 |
| YFT | 5342 |  | Cook Island | 197.483 | -7.333 | -0.0058 | 0.0024 | -2.4572 | 0.0140 | -4.141 | 348 |
| ALB | 5374 |  | Cook Island | 198.183 | -7.633 | -0.0089 | 0.0033 | -2.6774 | 0.0074 | -5.285 | 260 |
| YFT | 5467 |  | Cook Island | 201.917 | -8.600 | -0.0045 | 0.0012 | -3.6742 | 0.0002 | -11.743 | 894 |
| ALB | 5491 |  | Cook Island | 192.200 | -8.883 | -0.0055 | 0.0021 | -2.6598 | 0.0078 | -5.174 | 287 |
| ALB | 5495 |  | Cook Island | 192.767 | -8.917 | -0.0036 | 0.0024 | -1.5373 | 0.1243 | -0.397 | 257 |
| ALB | 5524 |  | Cook Island | 199.633 | -9.217 | -0.0023 | 0.0013 | -1.7005 | 0.0891 | -0.937 | 521 |
| ALB | 5560 |  | Cook Island | 200.033 | -9.550 | -0.0043 | 0.0026 | -1.6395 | 0.1012 | -0.729 | 224 |
| BET | 5593 |  | Cook Island | 202.117 | -9.767 | -0.0035 | 0.0014 | -2.5824 | 0.0098 | -4.769 | 746 |
| YFT | 5593 |  | Cook Island | 202.117 | -9.767 | -0.0027 | 0.0014 | -1.8772 | 0.0606 | -1.578 | 746 |
| ALB | 5646 |  | Cook Island | 200.050 | -10.233 | -0.0033 | 0.0020 | -1.6578 | 0.0974 | -0.789 | 376 |
| BET | 5650 |  | Cook Island | 198.583 | -10.267 | -0.0023 | 0.0013 | -1.8592 | 0.0631 | -1.513 | 798 |
| YFT | 5650 |  | Cook Island | 198.583 | -10.267 | -0.0024 | 0.0013 | -1.8132 | 0.0699 | -1.341 | 798 |
| BET | 5691 |  | Cook Island | 194.800 | -10.583 | -0.0040 | 0.0025 | -1.6123 | 0.1070 | -0.632 | 297 |
| YFT | 5691 |  | Cook Island | 194.800 | -10.583 | -0.0085 | 0.0027 | -3.1804 | 0.0015 | -8.237 | 297 |
| BET | 5710 |  | Cook Island | 194.050 | -10.700 | -0.0059 | 0.0021 | -2.7770 | 0.0055 | -5.801 | 381 |
| YFT | 5710 |  | Cook Island | 194.050 | -10.700 | -0.0104 | 0.0023 | -4.5629 | 0.0000 | -19.041 | 381 |
| ALB | 5766 |  | Cook Island | 199.633 | -11.117 | -0.0022 | 0.0010 | -2.2593 | 0.0239 | -3.196 | 960 |
| BET | 5766 |  | Cook Island | 199.633 | -11.117 | -0.0018 | 0.0012 | -1.5219 | 0.1281 | -0.359 | 960 |
| BET | 5795 |  | Cook Island | 194.200 | -11.317 | -0.0068 | 0.0038 | -1.7904 | 0.0734 | -1.244 | 111 |
| YFT | 5795 |  | Cook Island | 194.200 | -11.317 | -0.0135 | 0.0041 | -3.2557 | 0.0011 | -8.720 | 111 |
| BET | 5820 |  | Cook Island | 191.733 | -11.483 | -0.0015 | 0.0010 | -1.5644 | 0.1177 | -0.467 | 1519 |
| YFT | 5820 |  | Cook Island | 191.733 | -11.483 | -0.0017 | 0.0011 | -1.5165 | 0.1294 | -0.318 | 1519 |
| BET | 5916 |  | Cook Island | 193.600 | -12.450 | -0.0022 | 0.0015 | -1.4633 | 0.1434 | -0.168 | 691 |
| BET | 5940 |  | Cook Island | 192.767 | -12.583 | -0.0021 | 0.0009 | -2.2561 | 0.0241 | -3.152 | 1225 |
| YFT | 6066 |  | Cook Island | 196.283 | -13.300 | -0.0059 | 0.0027 | -2.1808 | 0.0293 | -2.904 | 151 |
| YFT | 6075 |  | Cook Island | 196.400 | -13.450 | -0.0053 | 0.0030 | -1.8072 | 0.0709 | -1.387 | 169 |
| YFT | 6133 |  | Cook Island | 198.767 | -14.100 | -0.0061 | 0.0023 | -2.5965 | 0.0096 | -5.377 | 180 |
| BET | 6269 |  | Cook Island | 196.267 | -14.933 | -0.0043 | 0.0026 | -1.6176 | 0.1060 | -0.782 | 141 |
| BET | 6636 |  | Cook Island | 196.583 | -17.967 | -0.0040 | 0.0019 | -2.0841 | 0.0376 | -2.783 | 195 |
| YFT | 6636 |  | Cook Island | 196.583 | -17.967 | -0.0094 | 0.0024 | -3.9401 | 0.0001 | -14.936 | 195 |
| YFT | 6675 |  | Cook Island | 195.483 | -18.333 | -0.0046 | 0.0031 | -1.4644 | 0.1436 | -0.361 | 123 |
| BET | 6688 |  | Cook Island | 201.617 | -18.467 | -0.0040 | 0.0028 | -1.4610 | 0.1443 | -0.265 | 113 |
| YFT | 6688 |  | Cook Island | 201.617 | -18.467 | -0.0048 | 0.0033 | -1.4751 | 0.1405 | -0.309 | 113 |
| YFT | 6759 | Eclipse | Cook Island | 200.650 | -19.167 | -0.0047 | 0.0015 | -3.0632 | 0.0022 | -7.728 | 318 |
| ALB | 6877 |  | Cook Island | 203.433 | -20.450 | -0.0055 | 0.0028 | -1.9808 | 0.0481 | -2.340 | 106 |
| YFT | 3201 |  | Federated States of Micronesia | 140.100 | 12.900 | -0.0171 | 0.0073 | -2.3475 | 0.0190 | -3.728 | 111 |
| ALB | 3264 |  | Federated States of Micronesia | 140.950 | 12.400 | -0.0082 | 0.0030 | -2.7211 | 0.0065 | -5.600 | 119 |
| ALB | 3321 |  | Federated States of Micronesia | 141.633 | 11.983 | -0.0096 | 0.0037 | -2.6087 | 0.0091 | -4.989 | 127 |
| ALB | 3462 |  | Federated States of Micronesia | 144.200 | 10.917 | -0.0011 | 0.0007 | -1.6775 | 0.0935 | -0.842 | 666 |
| ALB | 3484 |  | Federated States of Micronesia | 148.667 | 10.783 | -0.0011 | 0.0005 | -2.0175 | 0.0437 | -2.097 | 1086 |
| ALB | 3512 |  | Federated States of Micronesia | 146.767 | 10.550 | -0.0014 | 0.0010 | -1.4494 | 0.1473 | -0.113 | 924 |
| ALB | 3523 |  | Federated States of Micronesia | 144.500 | 10.483 | -0.0016 | 0.0005 | -3.0266 | 0.0025 | -7.234 | 1190 |
| BET | 3530 |  | Federated States of Micronesia | 145.050 | 10.433 | -0.0040 | 0.0023 | -1.7800 | 0.0751 | -1.190 | 880 |
| ALB | 3566 |  | Federated States of Micronesia | 148.333 | 10.200 | -0.0020 | 0.0013 | -1.4947 | 0.1350 | -0.247 | 695 |
| ALB | 3608 |  | Federated States of Micronesia | 156.483 | 9.967 | -0.0020 | 0.0004 | -5.1682 | 0.0000 | -24.853 | 2676 |
| ALB | 3617 |  | Federated States of Micronesia | 146.950 | 9.867 | -0.0010 | 0.0006 | -1.6926 | 0.0905 | -0.881 | 1557 |
| BET | 3617 |  | Federated States of Micronesia | 146.950 | 9.867 | -0.0015 | 0.0009 | -1.5663 | 0.1173 | -0.467 | 1557 |
| YFT | 3617 |  | Federated States of Micronesia | 146.950 | 9.867 | -0.0028 | 0.0011 | -2.4621 | 0.0138 | -4.095 | 1557 |
| ALB | 3628 |  | Federated States of Micronesia | 145.567 | 9.783 | -0.0018 | 0.0007 | -2.7164 | 0.0066 | -5.421 | 1064 |
| BET | 3628 |  | Federated States of Micronesia | 145.567 | 9.783 | -0.0027 | 0.0010 | -2.6009 | 0.0093 | -4.803 | 1064 |
| YFT | 3724 |  | Federated States of Micronesia | 139.217 | 9.167 | -0.0084 | 0.0056 | -1.5049 | 0.1324 | -0.293 | 144 |
| YFT | 3730 |  | Federated States of Micronesia | 144.950 | 9.133 | -0.0037 | 0.0013 | -2.8997 | 0.0037 | -6.466 | 781 |
| BET | 3743 |  | Federated States of Micronesia | 139.867 | 9.083 | -0.0092 | 0.0059 | -1.5617 | 0.1184 | -0.465 | 133 |
| BET | 3811 |  | Federated States of Micronesia | 140.800 | 8.683 | -0.0126 | 0.0052 | -2.4356 | 0.0149 | -3.992 | 122 |
| BET | 3872 |  | Federated States of Micronesia | 159.883 | 8.300 | -0.0018 | 0.0007 | -2.7100 | 0.0067 | -5.428 | 2208 |
| YFT | 3872 |  | Federated States of Micronesia | 159.883 | 8.300 | -0.0022 | 0.0008 | -2.7971 | 0.0052 | -5.912 | 2208 |
| ALB | 3894 |  | Federated States of Micronesia | 141.033 | 8.200 | -0.0032 | 0.0013 | -2.4890 | 0.0128 | -4.254 | 188 |
| BET | 3932 |  | Federated States of Micronesia | 140.600 | 8.017 | -0.0028 | 0.0018 | -1.5070 | 0.1319 | -0.291 | 277 |
| YFT | 4000 |  | Federated States of Micronesia | 141.533 | 7.550 | -0.0052 | 0.0031 | -1.6618 | 0.0966 | -0.785 | 219 |
| ALB | 4069 |  | Federated States of Micronesia | 142.583 | 7.133 | -0.0008 | 0.0005 | -1.6802 | 0.0930 | -0.851 | 398 |
| BET | 4088 |  | Federated States of Micronesia | 146.800 | 7.000 | -0.0035 | 0.0023 | -1.5181 | 0.1290 | -0.331 | 240 |
| ALB | 4090 |  | Federated States of Micronesia | 142.067 | 6.983 | -0.0020 | 0.0010 | -1.9649 | 0.0495 | -1.896 | 208 |
| YFT | 4095 |  | Federated States of Micronesia | 144.867 | 6.917 | -0.0083 | 0.0044 | -1.9102 | 0.0562 | -1.688 | 215 |
| YFT | 4101 |  | Federated States of Micronesia | 136.550 | 6.867 | -0.0013 | 0.0008 | -1.5593 | 0.1190 | -0.448 | 1850 |
| BET | 4159 |  | Federated States of Micronesia | 141.900 | 6.417 | -0.0023 | 0.0012 | -1.8771 | 0.0605 | -1.554 | 497 |
| YFT | 4165 |  | Federated States of Micronesia | 141.733 | 6.383 | -0.0083 | 0.0028 | -3.0071 | 0.0026 | -7.113 | 178 |
| ALB | 4167 |  | Federated States of Micronesia | 143.733 | 6.350 | -0.0006 | 0.0003 | -1.8934 | 0.0583 | -1.627 | 859 |
| BET | 4169 |  | Federated States of Micronesia | 149.501 | 6.299 | -0.0083 | 0.0045 | -1.8499 | 0.0643 | -1.442 | 114 |
| YFT | 4181 |  | Federated States of Micronesia | 147.950 | 6.233 | -0.0027 | 0.0010 | -2.8450 | 0.0045 | -6.164 | 1491 |
| BET | 4203 |  | Federated States of Micronesia | 154.133 | 6.050 | -0.0032 | 0.0018 | -1.7411 | 0.0817 | -1.054 | 310 |
| ALB | 4208 |  | Federated States of Micronesia | 141.067 | 6.033 | -0.0004 | 0.0002 | -1.8464 | 0.0649 | -1.436 | 1212 |
| BET | 4208 |  | Federated States of Micronesia | 141.067 | 6.033 | -0.0023 | 0.0007 | -3.1073 | 0.0019 | -7.728 | 1212 |
| BET | 4214 |  | Federated States of Micronesia | 145.428 | 5.961 | -0.0021 | 0.0014 | -1.4950 | 0.1350 | -0.258 | 562 |
| BET | 4231 |  | Federated States of Micronesia | 140.250 | 5.833 | -0.0017 | 0.0007 | -2.2892 | 0.0221 | -3.282 | 1556 |
| ALB | 4300 |  | Federated States of Micronesia | 150.167 | 5.333 | -0.0003 | 0.0002 | -1.8476 | 0.0647 | -1.400 | 1548 |
| BET | 4305 |  | Federated States of Micronesia | 158.167 | 5.283 | -0.0007 | 0.0005 | -1.4794 | 0.1391 | -0.205 | 3849 |
| YFT | 4305 |  | Federated States of Micronesia | 158.167 | 5.283 | -0.0014 | 0.0005 | -2.7177 | 0.0066 | -5.439 | 3849 |
| BET | 4342 |  | Federated States of Micronesia | 148.283 | 4.933 | -0.0078 | 0.0040 | -1.9640 | 0.0495 | -1.875 | 320 |
| YFT | 4350 | Dmitri Mendeleev | Federated States of Micronesia | 154.967 | 4.867 | -0.0015 | 0.0007 | -2.2033 | 0.0276 | -2.884 | 3000 |
| BET | 4353 |  | Federated States of Micronesia | 150.000 | 4.800 | -0.0029 | 0.0019 | -1.5292 | 0.1262 | -0.310 | 688 |
| BET | 4442 |  | Federated States of Micronesia | 148.633 | 3.900 | -0.0024 | 0.0009 | -2.6703 | 0.0076 | -5.158 | 1256 |
| ALB | 5712 |  | Fiji | 174.433 | -10.709 | -0.0015 | 0.0008 | -1.9942 | 0.0462 | -2.031 | 1924 |
| ALB | 5797 |  | Fiji | 174.217 | -11.333 | -0.0056 | 0.0016 | -3.5417 | 0.0004 | -10.685 | 438 |
| BET | 5828 | Alexa | Fiji | 175.367 | -11.550 | -0.0119 | 0.0015 | -7.9895 | 0.0000 | -62.401 | 692 |
| YFT | 5936 |  | Fiji | 176.867 | -12.550 | -0.0120 | 0.0072 | -1.6739 | 0.0942 | -0.836 | 107 |
| BET | 5941 |  | Fiji | 177.550 | -12.583 | -0.0028 | 0.0020 | -1.4340 | 0.1516 | -0.087 | 460 |
| YFT | 5941 |  | Fiji | 177.550 | -12.583 | -0.0065 | 0.0024 | -2.7369 | 0.0062 | -5.598 | 460 |
| BET | 5958 |  | Fiji | 178.833 | -12.717 | -0.0023 | 0.0013 | -1.8260 | 0.0679 | -1.400 | 708 |
| YFT | 5967 |  | Fiji | 179.033 | -12.733 | -0.0031 | 0.0022 | -1.4037 | 0.1605 | -0.012 | 441 |
| YFT | 5977 |  | Fiji | 177.533 | -12.783 | -0.0047 | 0.0019 | -2.4216 | 0.0155 | -3.948 | 478 |
| YFT | 5981 |  | Fiji | 176.033 | -12.817 | -0.0038 | 0.0022 | -1.7592 | 0.0786 | -1.128 | 451 |
| YFT | 5999 |  | Fiji | 177.200 | -12.917 | -0.0021 | 0.0014 | -1.5552 | 0.1199 | -0.451 | 717 |
| BET | 6040 |  | Fiji | 175.167 | -13.183 | -0.0020 | 0.0011 | -1.7340 | 0.0830 | -1.044 | 887 |
| YFT | 6040 |  | Fiji | 175.167 | -13.183 | -0.0040 | 0.0014 | -2.8063 | 0.0050 | -5.970 | 887 |
| BET | 6050 |  | Fiji | 174.133 | -13.233 | -0.0033 | 0.0014 | -2.3404 | 0.0193 | -3.549 | 447 |
| YFT | 6050 |  | Fiji | 174.133 | -13.233 | -0.0044 | 0.0018 | -2.4941 | 0.0126 | -4.301 | 447 |
| BET | 6063 |  | Fiji | 174.600 | -13.283 | -0.0024 | 0.0010 | -2.3777 | 0.0174 | -3.725 | 876 |
| YFT | 6063 |  | Fiji | 174.600 | -13.283 | -0.0032 | 0.0013 | -2.5551 | 0.0106 | -4.611 | 876 |
| YFT | 6082 |  | Fiji | 175.950 | -13.533 | -0.0017 | 0.0010 | -1.6441 | 0.1002 | -0.737 | 1366 |
| YFT | 6329 |  | Fiji | 177.400 | -15.583 | -0.0033 | 0.0011 | -2.9361 | 0.0033 | -6.703 | 877 |
| BET | 6355 | Braemar | Fiji | 176.900 | -15.783 | -0.0027 | 0.0018 | -1.4958 | 0.1347 | -0.256 | 260 |
| ALB | 6434 |  | Fiji | 176.533 | -16.417 | -0.0082 | 0.0035 | -2.3763 | 0.0175 | -3.683 | 377 |
| BET | 6434 |  | Fiji | 176.533 | -16.417 | -0.0045 | 0.0032 | -1.4321 | 0.1521 | -0.064 | 377 |
| ALB | 6466 |  | Fiji | 175.767 | -16.583 | -0.0061 | 0.0025 | -2.4254 | 0.0153 | -3.919 | 533 |
| BET | 6467 |  | Fiji | 177.167 | -16.583 | -0.0038 | 0.0011 | -3.3941 | 0.0007 | -9.594 | 1218 |
| BET | 6469 |  | Fiji | 174.083 | -16.600 | -0.0013 | 0.0007 | -2.0001 | 0.0455 | -2.026 | 1493 |
| YFT | 6530 |  | Fiji | 182.317 | -16.967 | -0.0042 | 0.0015 | -2.7675 | 0.0057 | -5.780 | 879 |
| ALB | 6609 |  | Fiji | 175.717 | -17.700 | -0.0033 | 0.0007 | -4.5366 | 0.0000 | -18.683 | 1652 |
| ALB | 6626 | Argo Reef | Fiji | 181.710 | -17.883 | -0.0028 | 0.0011 | -2.5595 | 0.0105 | -4.629 | 1093 |
| BET | 6626 | Argo Reef | Fiji | 181.710 | -17.883 | -0.0037 | 0.0010 | -3.5994 | 0.0003 | -11.105 | 1093 |
| BET | 6633 |  | Fiji | 175.400 | -17.917 | -0.0021 | 0.0009 | -2.3285 | 0.0199 | -3.452 | 968 |
| ALB | 6670 |  | Fiji | 175.967 | -18.283 | -0.0036 | 0.0021 | -1.7253 | 0.0845 | -0.992 | 635 |
| ALB | 6687 |  | Fiji | 176.467 | -18.450 | -0.0036 | 0.0009 | -3.8518 | 0.0001 | -12.910 | 969 |
| ALB | 6692 |  | Fiji | 176.733 | -18.483 | -0.0040 | 0.0009 | -4.5591 | 0.0000 | -18.890 | 1634 |
| ALB | 6701 |  | Fiji | 179.433 | -18.550 | -0.0014 | 0.0008 | -1.8020 | 0.0716 | -1.269 | 1771 |
| ALB | 6749 |  | Fiji | 179.167 | -18.933 | -0.0010 | 0.0007 | -1.4203 | 0.1555 | -0.013 | 1765 |
| BET | 6775 |  | Fiji | 179.133 | -19.250 | -0.0015 | 0.0008 | -1.9501 | 0.0512 | -1.826 | 1843 |
| ALB | 6786 |  | Fiji | 177.833 | -19.467 | -0.0036 | 0.0008 | -4.4911 | 0.0000 | -18.261 | 1918 |
| YFT | 6791 | Lau | Fiji | 181.800 | -19.450 | -0.0018 | 0.0010 | -1.7945 | 0.0728 | -1.288 | 1465 |
| BET | 6801 | Denham | Fiji | 178.483 | -19.683 | -0.0019 | 0.0010 | -1.9104 | 0.0561 | -1.669 | 1216 |
| YFT | 6801 | Denham | Fiji | 178.483 | -19.683 | -0.0026 | 0.0012 | -2.2495 | 0.0245 | -3.088 | 1216 |
| YFT | 6862 |  | Fiji | 176.933 | -20.350 | -0.0017 | 0.0011 | -1.5133 | 0.1302 | -0.305 | 906 |
| YFT | 6885 | Moore | Fiji | 177.483 | -20.533 | -0.0042 | 0.0012 | -3.4157 | 0.0006 | -9.746 | 629 |
| ALB | 6888 |  | Fiji | 182.867 | -20.567 | -0.0058 | 0.0014 | -4.0553 | 0.0001 | -14.660 | 759 |
| BET | 6888 |  | Fiji | 182.867 | -20.567 | -0.0031 | 0.0012 | -2.5891 | 0.0096 | -4.795 | 759 |
| YFT | 6909 | Moore | Fiji | 177.267 | -20.683 | -0.0063 | 0.0013 | -4.8308 | 0.0000 | -21.491 | 528 |
| ALB | 6947 |  | Fiji | 173.717 | -21.567 | -0.0032 | 0.0016 | -1.9545 | 0.0507 | -1.890 | 239 |
| BET | 6947 |  | Fiji | 173.717 | -21.567 | -0.0032 | 0.0014 | -2.2391 | 0.0252 | -3.105 | 239 |
| YFT | 6947 |  | Fiji | 173.717 | -21.567 | -0.0051 | 0.0020 | -2.4957 | 0.0126 | -4.341 | 239 |
| ALB | 6951 |  | Fiji | 179.167 | -21.617 | -0.0032 | 0.0011 | -3.0221 | 0.0025 | -7.288 | 920 |
| ALB | 6965 |  | Fiji | 176.150 | -21.267 | -0.0042 | 0.0012 | -3.5738 | 0.0004 | -10.895 | 797 |
| BET | 6965 |  | Fiji | 176.150 | -21.267 | -0.0026 | 0.0011 | -2.4584 | 0.0140 | -4.104 | 797 |
| BET | 6987 | Colwyn | Fiji | 174.333 | -21.850 | -0.0020 | 0.0011 | -1.7767 | 0.0757 | -1.202 | 463 |
| YFT | 6987 | Colwyn | Fiji | 174.333 | -21.850 | -0.0038 | 0.0016 | -2.4022 | 0.0163 | -3.852 | 463 |
| ALB | 6997 |  | Fiji | 176.667 | -21.983 | -0.0016 | 0.0010 | -1.6064 | 0.1082 | -0.614 | 960 |
| BET | 6997 |  | Fiji | 176.667 | -21.983 | -0.0015 | 0.0009 | -1.6977 | 0.0896 | -0.920 | 960 |
| ALB | 7032 |  | Fiji | 181.833 | -22.283 | -0.0136 | 0.0034 | -4.0182 | 0.0001 | -14.686 | 140 |
| YFT | 7096 |  | Fiji | 174.300 | -22.950 | -0.0024 | 0.0011 | -2.2409 | 0.0251 | -3.123 | 847 |
| BET | 7152 |  | Fiji | 181.128 | -23.628 | -0.0043 | 0.0028 | -1.5170 | 0.1294 | -0.408 | 136 |
| ALB | 8594 | Sud Bayonnaise | Fiji | 180.067 | -13.717 | -0.0028 | 0.0012 | -2.4360 | 0.0149 | -4.128 | 759 |
| YFT | 8595 |  | Fiji | 181.042 | -16.483 | -0.0018 | 0.0012 | -1.5274 | 0.1267 | -0.364 | 1099 |
| YFT | 8707 | Nukucikobia | Fiji | 181.417 | -17.150 | -0.0140 | 0.0040 | -3.5134 | 0.0004 | -10.480 | 283 |
| ALB | 8708 | Katafaga | Fiji | 181.433 | -17.400 | -0.0044 | 0.0024 | -1.8278 | 0.0676 | -1.378 | 202 |
| ALB | 8709 | Taveuni | Fiji | 180.017 | -17.283 | -0.0027 | 0.0009 | -3.2015 | 0.0014 | -8.340 | 1812 |
| YFT | 5376 |  | French Polynesia | 219.450 | -7.633 | -0.0090 | 0.0020 | -4.5532 | 0.0000 | -19.098 | 407 |
| YFT | 5393 |  | French Polynesia | 220.067 | -7.850 | -0.0034 | 0.0024 | -1.4079 | 0.1593 | -0.026 | 279 |
| YFT | 5395 |  | French Polynesia | 219.050 | -7.867 | -0.0098 | 0.0038 | -2.5495 | 0.0108 | -4.623 | 229 |
| YFT | 5401 |  | French Polynesia | 218.583 | -7.930 | -0.0053 | 0.0028 | -1.9211 | 0.0548 | -1.769 | 187 |
| YFT | 5416 |  | French Polynesia | 218.050 | -8.096 | -0.0051 | 0.0018 | -2.8903 | 0.0039 | -6.536 | 540 |
| BET | 5427 |  | French Polynesia | 218.617 | -8.129 | -0.0072 | 0.0038 | -1.9075 | 0.0565 | -1.710 | 147 |
| YFT | 5427 |  | French Polynesia | 218.617 | -8.129 | -0.0090 | 0.0046 | -1.9650 | 0.0495 | -1.936 | 147 |
| YFT | 5482 | Tupa | French Polynesia | 220.283 | -8.733 | -0.0095 | 0.0021 | -4.5778 | 0.0000 | -19.325 | 368 |
| YFT | 5509 | Marchand | French Polynesia | 219.400 | -9.150 | -0.0083 | 0.0019 | -4.3487 | 0.0000 | -17.184 | 468 |
| YFT | 5563 | Kena | French Polynesia | 220.233 | -9.600 | -0.0045 | 0.0029 | -1.5620 | 0.1184 | -0.483 | 275 |
| BET | 5598 |  | French Polynesia | 220.433 | -9.783 | -0.0063 | 0.0019 | -3.3258 | 0.0009 | -9.243 | 296 |
| YFT | 5598 |  | French Polynesia | 220.433 | -9.783 | -0.0059 | 0.0024 | -2.4353 | 0.0149 | -4.032 | 296 |
| ALB | 5601 |  | French Polynesia | 221.700 | -9.850 | -0.0075 | 0.0024 | -3.1059 | 0.0019 | -7.853 | 270 |
| ALB | 5609 |  | French Polynesia | 221.850 | -9.967 | -0.0079 | 0.0030 | -2.6346 | 0.0085 | -5.096 | 113 |
| YFT | 5609 |  | French Polynesia | 221.850 | -9.967 | -0.0064 | 0.0030 | -2.1617 | 0.0307 | -2.778 | 113 |
| YFT | 5634 |  | French Polynesia | 217.783 | -10.100 | -0.0042 | 0.0014 | -2.9671 | 0.0030 | -6.957 | 797 |
| ALB | 5641 | Meihano | French Polynesia | 222.100 | -10.217 | -0.0078 | 0.0025 | -3.0975 | 0.0020 | -7.808 | 180 |
| YFT | 5641 | Meihano | French Polynesia | 222.100 | -10.217 | -0.0048 | 0.0024 | -2.0096 | 0.0445 | -2.131 | 180 |
| ALB | 5642 |  | French Polynesia | 225.083 | -10.200 | -0.0052 | 0.0018 | -2.9191 | 0.0036 | -6.829 | 445 |
| ALB | 5665 |  | French Polynesia | 224.350 | -10.367 | -0.0070 | 0.0019 | -3.6168 | 0.0003 | -11.444 | 361 |
| ALB | 5686 |  | French Polynesia | 223.467 | -10.550 | -0.0072 | 0.0023 | -3.0875 | 0.0020 | -7.766 | 237 |
| ALB | 5701 |  | French Polynesia | 223.050 | -10.633 | -0.0043 | 0.0030 | -1.4513 | 0.1468 | -0.157 | 153 |
| ALB | 5722 |  | French Polynesia | 222.233 | -10.783 | -0.0173 | 0.0068 | -2.5405 | 0.0111 | -4.590 | 142 |
| BET | 5722 |  | French Polynesia | 222.233 | -10.783 | -0.0095 | 0.0050 | -1.8810 | 0.0600 | -1.614 | 142 |
| YFT | 5727 |  | French Polynesia | 224.133 | -10.817 | -0.0041 | 0.0022 | -1.8940 | 0.0583 | -1.688 | 219 |
| BET | 5751 |  | French Polynesia | 218.367 | -11.017 | -0.0019 | 0.0009 | -2.0187 | 0.0436 | -2.133 | 1445 |
| ALB | 5775 |  | French Polynesia | 220.650 | -11.200 | -0.0031 | 0.0016 | -1.9511 | 0.0511 | -1.869 | 548 |
| BET | 5775 |  | French Polynesia | 220.650 | -11.200 | -0.0020 | 0.0013 | -1.5408 | 0.1234 | -0.414 | 548 |
| ALB | 5803 |  | French Polynesia | 221.833 | -11.383 | -0.0061 | 0.0032 | -1.9398 | 0.0525 | -1.843 | 167 |
| YFT | 5812 |  | French Polynesia | 221.650 | -11.450 | -0.0054 | 0.0023 | -2.4086 | 0.0161 | -3.920 | 274 |
| YFT | 5832 |  | French Polynesia | 220.950 | -11.633 | -0.0032 | 0.0016 | -2.0795 | 0.0376 | -2.402 | 497 |
| BET | 5845 |  | French Polynesia | 219.317 | -11.800 | -0.0022 | 0.0008 | -2.7880 | 0.0053 | -5.881 | 1690 |
| ALB | 5872 |  | French Polynesia | 215.467 | -12.117 | -0.0034 | 0.0012 | -2.8175 | 0.0049 | -6.036 | 429 |
| BET | 5872 |  | French Polynesia | 215.467 | -12.117 | -0.0030 | 0.0015 | -1.9925 | 0.0464 | -2.020 | 429 |
| YFT | 5872 |  | French Polynesia | 215.467 | -12.117 | -0.0026 | 0.0015 | -1.7822 | 0.0748 | -1.217 | 429 |
| ALB | 5881 |  | French Polynesia | 215.600 | -12.183 | -0.0025 | 0.0014 | -1.7622 | 0.0781 | -1.144 | 253 |
| BET | 5891 |  | French Polynesia | 211.233 | -12.250 | -0.0056 | 0.0015 | -3.6451 | 0.0003 | -11.423 | 631 |
| YFT | 5891 |  | French Polynesia | 211.233 | -12.250 | -0.0038 | 0.0015 | -2.5029 | 0.0123 | -4.331 | 631 |
| ALB | 6070 |  | French Polynesia | 212.117 | -13.392 | -0.0079 | 0.0028 | -2.8845 | 0.0039 | -6.387 | 394 |
| BET | 6070 |  | French Polynesia | 212.117 | -13.392 | -0.0115 | 0.0036 | -3.2058 | 0.0014 | -8.359 | 394 |
| YFT | 6070 |  | French Polynesia | 212.117 | -13.392 | -0.0105 | 0.0037 | -2.8023 | 0.0051 | -5.916 | 394 |
| ALB | 6090 |  | French Polynesia | 213.167 | -13.650 | -0.0037 | 0.0007 | -5.1300 | 0.0000 | -24.525 | 1257 |
| BET | 6090 |  | French Polynesia | 213.167 | -13.650 | -0.0017 | 0.0009 | -1.8064 | 0.0709 | -1.293 | 1257 |
| YFT | 6090 |  | French Polynesia | 213.167 | -13.650 | -0.0015 | 0.0010 | -1.4705 | 0.1415 | -0.182 | 1257 |
| BET | 6093 |  | French Polynesia | 209.733 | -13.683 | -0.0067 | 0.0026 | -2.5763 | 0.0100 | -4.716 | 294 |
| YFT | 6093 |  | French Polynesia | 209.733 | -13.683 | -0.0062 | 0.0028 | -2.2187 | 0.0265 | -2.981 | 294 |
| YFT | 6132 |  | French Polynesia | 210.900 | -14.083 | -0.0091 | 0.0032 | -2.8057 | 0.0050 | -5.935 | 329 |
| ALB | 6162 |  | French Polynesia | 211.917 | -14.319 | -0.0038 | 0.0009 | -4.1466 | 0.0000 | -15.324 | 1239 |
| BET | 6162 |  | French Polynesia | 211.917 | -14.319 | -0.0043 | 0.0011 | -3.9191 | 0.0001 | -13.476 | 1239 |
| YFT | 6178 |  | French Polynesia | 216.450 | -14.400 | -0.0104 | 0.0052 | -2.0021 | 0.0453 | -2.053 | 195 |
| BET | 6190 |  | French Polynesia | 210.100 | -14.450 | -0.0095 | 0.0027 | -3.4637 | 0.0005 | -10.111 | 533 |
| YFT | 6190 |  | French Polynesia | 210.100 | -14.450 | -0.0086 | 0.0030 | -2.8988 | 0.0038 | -6.485 | 533 |
| YFT | 6205 |  | French Polynesia | 218.250 | -14.551 | -0.0033 | 0.0014 | -2.4153 | 0.0158 | -3.925 | 656 |
| ALB | 6208 |  | French Polynesia | 217.017 | -14.584 | -0.0052 | 0.0021 | -2.5057 | 0.0122 | -4.358 | 349 |
| BET | 6227 |  | French Polynesia | 220.817 | -14.683 | -0.0044 | 0.0014 | -3.0416 | 0.0024 | -7.457 | 586 |
| YFT | 6227 |  | French Polynesia | 220.817 | -14.683 | -0.0046 | 0.0015 | -3.0560 | 0.0023 | -7.547 | 586 |
| BET | 6240 |  | French Polynesia | 210.883 | -14.816 | -0.0029 | 0.0013 | -2.3175 | 0.0205 | -3.418 | 929 |
| YFT | 6243 |  | French Polynesia | 218.200 | -14.783 | -0.0105 | 0.0024 | -4.4433 | 0.0000 | -18.037 | 180 |
| YFT | 6256 |  | French Polynesia | 217.600 | -14.883 | -0.0095 | 0.0033 | -2.9059 | 0.0037 | -6.566 | 317 |
| YFT | 6273 |  | French Polynesia | 210.450 | -14.981 | -0.0034 | 0.0021 | -1.6262 | 0.1040 | -0.669 | 336 |
| BET | 6284 |  | French Polynesia | 224.267 | -15.083 | -0.0046 | 0.0026 | -1.7763 | 0.0759 | -1.273 | 138 |
| YFT | 6284 |  | French Polynesia | 224.267 | -15.083 | -0.0048 | 0.0026 | -1.8015 | 0.0718 | -1.367 | 138 |
| BET | 6287 |  | French Polynesia | 223.783 | -15.180 | -0.0049 | 0.0020 | -2.4366 | 0.0149 | -4.181 | 228 |
| YFT | 6287 |  | French Polynesia | 223.783 | -15.180 | -0.0042 | 0.0021 | -1.9937 | 0.0463 | -2.141 | 228 |
| YFT | 6291 |  | French Polynesia | 216.367 | -15.183 | -0.0056 | 0.0018 | -3.1957 | 0.0014 | -8.374 | 364 |
| YFT | 6295 |  | French Polynesia | 224.933 | -15.250 | -0.0025 | 0.0016 | -1.5309 | 0.1260 | -0.435 | 410 |
| ALB | 6330 |  | French Polynesia | 209.933 | -15.617 | -0.0019 | 0.0009 | -2.2351 | 0.0254 | -3.042 | 1089 |
| BET | 6330 |  | French Polynesia | 209.933 | -15.617 | -0.0018 | 0.0008 | -2.2210 | 0.0264 | -2.979 | 1089 |
| YFT | 6367 |  | French Polynesia | 216.950 | -15.867 | -0.0046 | 0.0025 | -1.8419 | 0.0656 | -1.473 | 246 |
| ALB | 6397 |  | French Polynesia | 212.517 | -16.107 | -0.0059 | 0.0012 | -4.9515 | 0.0000 | -22.746 | 529 |
| BET | 6397 |  | French Polynesia | 212.517 | -16.107 | -0.0020 | 0.0011 | -1.7513 | 0.0799 | -1.101 | 529 |
| YFT | 6400 |  | French Polynesia | 211.883 | -16.107 | -0.0035 | 0.0009 | -3.9052 | 0.0001 | -13.378 | 1119 |
| YFT | 6455 |  | French Polynesia | 207.517 | -16.500 | -0.0082 | 0.0008 | -10.1280 | 0.0000 | -101.084 | 1443 |
| ALB | 6503 |  | French Polynesia | 213.833 | -16.783 | -0.0055 | 0.0014 | -3.8345 | 0.0001 | -12.971 | 344 |
| YFT | 6511 |  | French Polynesia | 206.400 | -16.850 | -0.0022 | 0.0014 | -1.5823 | 0.1136 | -0.539 | 954 |
| ALB | 6542 |  | French Polynesia | 214.200 | -17.083 | -0.0048 | 0.0018 | -2.7310 | 0.0064 | -5.613 | 299 |
| ALB | 6560 |  | French Polynesia | 213.483 | -17.283 | -0.0034 | 0.0015 | -2.2658 | 0.0235 | -3.207 | 316 |
| ALB | 6565 |  | French Polynesia | 215.800 | -17.317 | -0.0037 | 0.0018 | -2.0864 | 0.0371 | -2.503 | 208 |
| ALB | 6595 |  | French Polynesia | 219.917 | -17.598 | -0.0026 | 0.0016 | -1.6468 | 0.0999 | -0.899 | 214 |
| BET | 6597 |  | French Polynesia | 214.750 | -17.567 | -0.0030 | 0.0016 | -1.8924 | 0.0585 | -1.674 | 295 |
| YFT | 6606 |  | French Polynesia | 216.617 | -17.697 | -0.0045 | 0.0028 | -1.6253 | 0.1043 | -0.754 | 243 |
| BET | 6621 | 'Ori'o Mata / Savannah nord | French Polynesia | 205.917 | -17.783 | -0.0063 | 0.0025 | -2.4970 | 0.0126 | -4.353 | 313 |
| YFT | 6653 |  | French Polynesia | 215.317 | -18.100 | -0.0037 | 0.0022 | -1.6743 | 0.0942 | -0.885 | 273 |
| YFT | 6672 | Moua Pihaa | French Polynesia | 211.474 | -18.326 | -0.0036 | 0.0012 | -2.9999 | 0.0027 | -7.084 | 745 |
| YFT | 6676 | Honu / Savannah Ouest | French Polynesia | 205.920 | -18.380 | -0.0111 | 0.0064 | -1.7326 | 0.0833 | -1.075 | 124 |
| ALB | 6718 |  | French Polynesia | 212.900 | -18.717 | -0.0025 | 0.0009 | -2.6566 | 0.0079 | -5.166 | 767 |
| ALB | 6738 |  | French Polynesia | 218.467 | -18.883 | -0.0052 | 0.0017 | -3.0272 | 0.0025 | -7.614 | 151 |
| YFT | 6738 |  | French Polynesia | 218.467 | -18.883 | -0.0051 | 0.0026 | -1.9454 | 0.0519 | -1.978 | 151 |
| YFT | 6747 | Savannah / Tarava | French Polynesia | 208.000 | -19.000 | -0.0048 | 0.0026 | -1.8547 | 0.0637 | -1.489 | 119 |
| BET | 6764 | Ari'i Moana / Rigault de Genouilly | French Polynesia | 208.458 | -19.223 | -0.0080 | 0.0019 | -4.3171 | 0.0000 | -16.850 | 593 |
| ALB | 6769 |  | French Polynesia | 209.533 | -19.217 | -0.0042 | 0.0021 | -1.9371 | 0.0528 | -1.792 | 386 |
| BET | 6769 |  | French Polynesia | 209.533 | -19.217 | -0.0047 | 0.0021 | -2.2605 | 0.0238 | -3.164 | 386 |
| YFT | 6769 |  | French Polynesia | 209.533 | -19.217 | -0.0100 | 0.0024 | -4.1528 | 0.0000 | -15.417 | 386 |
| BET | 6771 | Punu Taipu / Savanah Est | French Polynesia | 209.015 | -19.283 | -0.0101 | 0.0018 | -5.5760 | 0.0000 | -29.394 | 671 |
| YFT | 6771 | Punu Taipu / Savanah Est | French Polynesia | 209.015 | -19.283 | -0.0057 | 0.0021 | -2.7199 | 0.0065 | -5.482 | 671 |
| YFT | 6785 |  | French Polynesia | 210.483 | -19.467 | -0.0034 | 0.0020 | -1.7435 | 0.0813 | -1.079 | 308 |
| YFT | 6823 |  | French Polynesia | 214.792 | -19.878 | -0.0051 | 0.0013 | -4.0613 | 0.0001 | -15.047 | 520 |
| BET | 7057 | Lotus | French Polynesia | 209.000 | -22.583 | -0.0118 | 0.0056 | -2.1232 | 0.0342 | -3.055 | 125 |
| YFT | 7057 | Lotus | French Polynesia | 209.000 | -22.583 | -0.0089 | 0.0059 | -1.5054 | 0.1328 | -0.546 | 125 |
| ALB | 7662 |  | French Polynesia | 219.117 | -27.430 | -0.0021 | 0.0014 | -1.4309 | 0.1529 | -0.188 | 172 |
| YFT | 7721 |  | French Polynesia | 216.150 | -28.000 | -0.0084 | 0.0029 | -2.8694 | 0.0042 | -6.876 | 112 |
| YFT | 7726 |  | French Polynesia | 213.367 | -28.050 | -0.0036 | 0.0021 | -1.7028 | 0.0890 | -1.147 | 217 |
| YFT | 7732 |  | French Polynesia | 216.617 | -28.160 | -0.0033 | 0.0023 | -1.4197 | 0.1561 | -0.166 | 214 |
| YFT | 7779 | Mc Donald | French Polynesia | 219.750 | -28.980 | -0.0062 | 0.0021 | -2.9665 | 0.0031 | -7.196 | 243 |
| ALB | 8783 | Repe | French Polynesia | 206.450 | -18.183 | -0.0060 | 0.0024 | -2.4721 | 0.0135 | -4.207 | 181 |
| ALB | 3245 |  | High seas | 149.669 | 12.502 | -0.0039 | 0.0014 | -2.8439 | 0.0045 | -6.246 | 428 |
| ALB | 3406 |  | High seas | 134.467 | 11.283 | -0.0022 | 0.0011 | -1.8902 | 0.0589 | -1.704 | 286 |
| YFT | 3965 |  | High seas | 203.199 | 7.717 | -0.0057 | 0.0041 | -1.4013 | 0.1613 | -0.026 | 110 |
| YFT | 3978 |  | High seas | 176.700 | 7.667 | -0.0080 | 0.0037 | -2.1242 | 0.0338 | -2.688 | 102 |
| BET | 4047 |  | High seas | 202.217 | 7.301 | -0.0079 | 0.0042 | -1.8822 | 0.0599 | -1.609 | 172 |
| YFT | 4201 |  | High seas | 203.800 | 6.026 | -0.0035 | 0.0017 | -2.0568 | 0.0398 | -2.285 | 458 |
| ALB | 4226 |  | High seas | 176.050 | 5.867 | -0.0018 | 0.0008 | -2.3461 | 0.0190 | -3.637 | 222 |
| ALB | 4311 |  | High seas | 191.733 | 5.250 | -0.0017 | 0.0009 | -1.7457 | 0.0820 | -1.441 | 312 |
| BET | 4313 |  | High seas | 203.868 | 5.194 | -0.0020 | 0.0013 | -1.5994 | 0.1098 | -0.588 | 526 |
| YFT | 4373 |  | High seas | 184.967 | 4.650 | -0.0035 | 0.0021 | -1.7135 | 0.0870 | -1.144 | 374 |
| YFT | 4420 |  | High seas | 176.267 | 4.150 | -0.0034 | 0.0020 | -1.7364 | 0.0826 | -1.071 | 418 |
| BET | 4476 |  | High seas | 186.533 | 3.433 | -0.0065 | 0.0029 | -2.2373 | 0.0255 | -3.283 | 115 |
| YFT | 4476 |  | High seas | 186.533 | 3.433 | -0.0084 | 0.0031 | -2.7392 | 0.0063 | -5.909 | 115 |
| YFT | 4509 |  | High seas | 178.533 | 3.017 | -0.0035 | 0.0021 | -1.6711 | 0.0949 | -0.904 | 423 |
| ALB | 4520 |  | High seas | 194.783 | 2.767 | -0.0041 | 0.0020 | -2.0055 | 0.0453 | -2.334 | 190 |
| BET | 4520 |  | High seas | 194.783 | 2.767 | -0.0037 | 0.0026 | -1.3968 | 0.1629 | -0.105 | 190 |
| ALB | 4532 |  | High seas | 149.683 | 2.667 | -0.0005 | 0.0003 | -1.8159 | 0.0694 | -1.312 | 1249 |
| BET | 4532 |  | High seas | 149.683 | 2.667 | -0.0024 | 0.0011 | -2.0518 | 0.0402 | -2.229 | 1249 |
| YFT | 4534 |  | High seas | 196.867 | 2.583 | -0.0046 | 0.0024 | -1.8962 | 0.0581 | -1.791 | 281 |
| ALB | 4561 |  | High seas | 195.000 | 2.150 | -0.0043 | 0.0023 | -1.8593 | 0.0633 | -1.728 | 111 |
| ALB | 4615 |  | High seas | 190.633 | 1.817 | -0.0051 | 0.0027 | -1.9116 | 0.0577 | -2.672 | 137 |
| BET | 4615 |  | High seas | 190.633 | 1.817 | -0.0044 | 0.0034 | -1.3186 | 0.1892 | -0.236 | 137 |
| BET | 4972 |  | High seas | 163.783 | -2.831 | -0.0045 | 0.0026 | -1.7792 | 0.0761 | -1.718 | 197 |
| YFT | 4974 |  | High seas | 194.783 | -2.867 | -0.0103 | 0.0032 | -3.2473 | 0.0012 | -8.968 | 160 |
| YFT | 5029 |  | High seas | 200.200 | -3.650 | -0.0060 | 0.0031 | -1.9102 | 0.0562 | -1.763 | 214 |
| YFT | 5034 |  | High seas | 194.967 | -3.717 | -0.0155 | 0.0055 | -2.7863 | 0.0054 | -6.019 | 105 |
| YFT | 5042 |  | High seas | 195.367 | -3.817 | -0.0083 | 0.0044 | -1.8967 | 0.0580 | -1.726 | 122 |
| ALB | 5045 |  | High seas | 195.967 | -3.850 | -0.0066 | 0.0030 | -2.2080 | 0.0273 | -3.072 | 143 |
| BET | 5071 |  | High seas | 196.483 | -4.200 | -0.0031 | 0.0022 | -1.3904 | 0.1646 | -0.007 | 187 |
| YFT | 5116 |  | High seas | 197.633 | -4.550 | -0.0048 | 0.0034 | -1.4014 | 0.1612 | -0.031 | 120 |
| BET | 5176 |  | High seas | 200.767 | -5.233 | -0.0033 | 0.0020 | -1.6400 | 0.1011 | -0.735 | 303 |
| YFT | 5182 |  | High seas | 193.983 | -5.267 | -0.0062 | 0.0033 | -1.8924 | 0.0585 | -1.645 | 193 |
| BET | 5186 |  | High seas | 197.233 | -5.317 | -0.0072 | 0.0036 | -2.0307 | 0.0423 | -2.220 | 130 |
| YFT | 5195 |  | High seas | 200.100 | -5.383 | -0.0055 | 0.0030 | -1.8648 | 0.0623 | -1.546 | 141 |
| YFT | 5202 |  | High seas | 199.900 | -5.483 | -0.0036 | 0.0025 | -1.4082 | 0.1591 | -0.024 | 163 |
| ALB | 5203 |  | High seas | 193.367 | -5.500 | -0.0043 | 0.0028 | -1.5548 | 0.1200 | -0.455 | 223 |
| ALB | 5223 |  | High seas | 198.933 | -5.650 | -0.0072 | 0.0028 | -2.5483 | 0.0109 | -4.641 | 213 |
| YFT | 5245 |  | High seas | 197.317 | -6.000 | -0.0027 | 0.0019 | -1.4490 | 0.1474 | -0.143 | 448 |
| ALB | 5257 |  | High seas | 192.633 | -6.117 | -0.0034 | 0.0017 | -2.0221 | 0.0432 | -2.136 | 637 |
| ALB | 5259 |  | High seas | 172.033 | -6.150 | -0.0041 | 0.0018 | -2.3285 | 0.0200 | -3.575 | 503 |
| YFT | 5268 |  | High seas | 182.400 | -6.250 | -0.0013 | 0.0008 | -1.7182 | 0.0858 | -0.984 | 2211 |
| ALB | 5269 |  | High seas | 192.200 | -6.250 | -0.0023 | 0.0014 | -1.6491 | 0.0992 | -0.750 | 918 |
| ALB | 5278 |  | High seas | 171.317 | -6.333 | -0.0071 | 0.0024 | -2.9507 | 0.0032 | -6.944 | 245 |
| YFT | 5421 |  | High seas | 172.267 | -8.050 | -0.0025 | 0.0014 | -1.7689 | 0.0770 | -1.186 | 724 |
| BET | 6080 |  | High seas | 173.333 | -13.517 | -0.0024 | 0.0008 | -2.9299 | 0.0034 | -6.694 | 1260 |
| YFT | 6080 |  | High seas | 173.333 | -13.517 | -0.0022 | 0.0011 | -2.0854 | 0.0371 | -2.406 | 1260 |
| YFT | 6384 |  | High seas | 173.417 | -15.967 | -0.0010 | 0.0007 | -1.4235 | 0.1546 | -0.040 | 2764 |
| ALB | 9273 |  | High seas | 176.094 | -24.641 | -0.0024 | 0.0013 | -1.9303 | 0.0536 | -1.809 | 585 |
| BET | 4366 |  | Kiribati (Gilbert Islands) | 172.900 | 4.700 | -0.0044 | 0.0026 | -1.6567 | 0.0976 | -0.781 | 189 |
| BET | 4407 | Sua | Kiribati (Gilbert Islands) | 172.436 | 4.269 | -0.0023 | 0.0015 | -1.4894 | 0.1364 | -0.248 | 397 |
| YFT | 4412 | Pulu | Kiribati (Gilbert Islands) | 172.899 | 4.268 | -0.0126 | 0.0070 | -1.7943 | 0.0728 | -1.266 | 141 |
| YFT | 4451 |  | Kiribati (Gilbert Islands) | 173.333 | 3.817 | -0.0029 | 0.0013 | -2.1761 | 0.0296 | -2.808 | 774 |
| BET | 4524 |  | Kiribati (Gilbert Islands) | 176.283 | 2.750 | -0.0029 | 0.0020 | -1.4423 | 0.1493 | -0.109 | 295 |
| YFT | 4549 |  | Kiribati (Gilbert Islands) | 173.550 | 2.333 | -0.0053 | 0.0030 | -1.7603 | 0.0784 | -1.150 | 212 |
| BET | 4560 |  | Kiribati (Gilbert Islands) | 174.417 | 2.167 | -0.0068 | 0.0046 | -1.4849 | 0.1376 | -0.239 | 137 |
| ALB | 4638 |  | Kiribati (Gilbert Islands) | 175.867 | 1.483 | -0.0023 | 0.0012 | -1.9707 | 0.0488 | -1.939 | 260 |
| ALB | 4655 |  | Kiribati (Gilbert Islands) | 176.967 | 1.250 | -0.0023 | 0.0010 | -2.2072 | 0.0274 | -2.956 | 408 |
| YFT | 4678 |  | Kiribati (Gilbert Islands) | 175.150 | 1.000 | -0.0025 | 0.0017 | -1.4771 | 0.1397 | -0.223 | 410 |
| YFT | 4724 |  | Kiribati (Gilbert Islands) | 177.250 | 0.400 | -0.0029 | 0.0020 | -1.4019 | 0.1610 | -0.005 | 381 |
| YFT | 4826 |  | Kiribati (Gilbert Islands) | 177.183 | -0.817 | -0.0029 | 0.0020 | -1.4294 | 0.1530 | -0.085 | 453 |
| ALB | 4830 | Palutu | Kiribati (Gilbert Islands) | 175.511 | -0.877 | -0.0021 | 0.0011 | -1.8501 | 0.0644 | -1.510 | 351 |
| ALB | 4858 |  | Kiribati (Gilbert Islands) | 178.500 | -1.217 | -0.0023 | 0.0013 | -1.8752 | 0.0608 | -1.564 | 504 |
| BET | 4858 |  | Kiribati (Gilbert Islands) | 178.500 | -1.217 | -0.0036 | 0.0016 | -2.2598 | 0.0239 | -3.175 | 504 |
| ALB | 4926 | Iroij | Kiribati (Gilbert Islands) | 176.211 | -2.104 | -0.0035 | 0.0022 | -1.6118 | 0.1071 | -0.633 | 209 |
| YFT | 4934 |  | Kiribati (Gilbert Islands) | 178.100 | -2.217 | -0.0079 | 0.0045 | -1.7705 | 0.0767 | -1.165 | 224 |
| BET | 5037 |  | Kiribati (Gilbert Islands) | 175.500 | -3.733 | -0.0031 | 0.0021 | -1.4604 | 0.1442 | -0.165 | 267 |
| YFT | 5037 |  | Kiribati (Gilbert Islands) | 175.500 | -3.733 | -0.0062 | 0.0023 | -2.7109 | 0.0067 | -5.458 | 267 |
| ALB | 5038 |  | Kiribati (Gilbert Islands) | 176.967 | -3.750 | -0.0025 | 0.0018 | -1.4301 | 0.1527 | -0.068 | 776 |
| ALB | 5074 |  | Kiribati (Gilbert Islands) | 177.833 | -4.217 | -0.0064 | 0.0012 | -5.4855 | 0.0000 | -28.367 | 1147 |
| YFT | 5074 |  | Kiribati (Gilbert Islands) | 177.833 | -4.217 | -0.0024 | 0.0012 | -2.0294 | 0.0424 | -2.163 | 1147 |
| YFT | 8742 | Kautu | Kiribati (Gilbert Islands) | 175.362 | -1.369 | -0.0075 | 0.0038 | -1.9871 | 0.0470 | -2.042 | 103 |
| YFT | 8747 |  | Kiribati (Gilbert Islands) | 179.976 | -2.287 | -0.0039 | 0.0019 | -2.0956 | 0.0362 | -2.456 | 422 |
| ALB | 4116 |  | Kiribati (Line Islands) | 202.300 | 6.750 | -0.0031 | 0.0007 | -4.6419 | 0.0000 | -19.789 | 312 |
| BET | 4182 |  | Kiribati (Line Islands) | 202.233 | 6.217 | -0.0038 | 0.0025 | -1.5222 | 0.1280 | -0.340 | 288 |
| BET | 4191 |  | Kiribati (Line Islands) | 200.467 | 6.117 | -0.0043 | 0.0017 | -2.6024 | 0.0093 | -4.864 | 470 |
| BET | 4282 |  | Kiribati (Line Islands) | 203.167 | 5.483 | -0.0032 | 0.0020 | -1.6011 | 0.1094 | -0.588 | 480 |
| ALB | 4325 | Christmas | Kiribati (Line Islands) | 200.050 | 5.116 | -0.0032 | 0.0020 | -1.6342 | 0.1023 | -0.702 | 289 |
| YFT | 4361 |  | Kiribati (Line Islands) | 199.417 | 4.733 | -0.0083 | 0.0051 | -1.6244 | 0.1043 | -0.676 | 242 |
| BET | 4391 |  | Kiribati (Line Islands) | 201.033 | 4.450 | -0.0032 | 0.0017 | -1.9470 | 0.0516 | -1.825 | 471 |
| ALB | 4498 |  | Kiribati (Line Islands) | 200.117 | 3.133 | -0.0016 | 0.0007 | -2.3174 | 0.0205 | -3.450 | 469 |
| BET | 4498 |  | Kiribati (Line Islands) | 200.117 | 3.133 | -0.0027 | 0.0013 | -2.0070 | 0.0448 | -2.088 | 469 |
| BET | 4506 |  | Kiribati (Line Islands) | 200.700 | 3.050 | -0.0044 | 0.0028 | -1.5519 | 0.1207 | -0.443 | 260 |
| YFT | 4630 |  | Kiribati (Line Islands) | 202.983 | 1.667 | -0.0052 | 0.0027 | -1.9608 | 0.0501 | -2.055 | 271 |
| ALB | 4647 | Gagarin | Kiribati (Line Islands) | 205.800 | 1.291 | -0.0020 | 0.0014 | -1.4742 | 0.1410 | -0.417 | 227 |
| ALB | 4684 |  | Kiribati (Line Islands) | 204.233 | 0.900 | -0.0042 | 0.0016 | -2.6172 | 0.0090 | -5.417 | 115 |
| YFT | 4904 | Menard | Kiribati (Line Islands) | 204.933 | -1.717 | -0.0019 | 0.0010 | -1.9109 | 0.0560 | -1.688 | 1365 |
| BET | 5103 | Malden | Kiribati (Line Islands) | 204.954 | -4.523 | -0.0016 | 0.0011 | -1.4494 | 0.1473 | -0.119 | 752 |
| BET | 5138 |  | Kiribati (Line Islands) | 206.667 | -4.767 | -0.0015 | 0.0009 | -1.7160 | 0.0862 | -0.970 | 1189 |
| ALB | 5153 |  | Kiribati (Line Islands) | 202.267 | -4.983 | -0.0042 | 0.0013 | -3.3122 | 0.0009 | -9.107 | 904 |
| ALB | 5155 |  | Kiribati (Line Islands) | 201.800 | -5.017 | -0.0039 | 0.0021 | -1.8360 | 0.0664 | -1.417 | 375 |
| ALB | 5362 | Wageman | Kiribati (Line Islands) | 208.450 | -7.560 | -0.0062 | 0.0017 | -3.6915 | 0.0002 | -11.758 | 557 |
| BET | 5362 | Wageman | Kiribati (Line Islands) | 208.450 | -7.560 | -0.0019 | 0.0011 | -1.6544 | 0.0981 | -0.765 | 557 |
| ALB | 5399 |  | Kiribati (Line Islands) | 208.783 | -7.900 | -0.0028 | 0.0014 | -1.9973 | 0.0458 | -2.031 | 804 |
| BET | 5399 |  | Kiribati (Line Islands) | 208.783 | -7.900 | -0.0026 | 0.0009 | -2.7457 | 0.0061 | -5.616 | 804 |
| BET | 5400 |  | Kiribati (Line Islands) | 204.950 | -7.950 | -0.0012 | 0.0008 | -1.4912 | 0.1360 | -0.256 | 1621 |
| ALB | 5431 |  | Kiribati (Line Islands) | 208.886 | -8.237 | -0.0048 | 0.0021 | -2.2156 | 0.0267 | -2.960 | 466 |
| BET | 5431 |  | Kiribati (Line Islands) | 208.886 | -8.237 | -0.0021 | 0.0014 | -1.4718 | 0.1411 | -0.189 | 466 |
| ALB | 5476 |  | Kiribati (Line Islands) | 206.550 | -8.692 | -0.0016 | 0.0011 | -1.4538 | 0.1461 | -0.142 | 1797 |
| ALB | 5511 |  | Kiribati (Line Islands) | 209.733 | -9.050 | -0.0028 | 0.0014 | -1.9527 | 0.0509 | -1.858 | 872 |
| YFT | 5557 |  | Kiribati (Line Islands) | 209.717 | -9.553 | -0.0050 | 0.0015 | -3.3000 | 0.0010 | -9.019 | 277 |
| BET | 5562 |  | Kiribati (Line Islands) | 209.983 | -9.586 | -0.0025 | 0.0014 | -1.7833 | 0.0746 | -1.219 | 391 |
| YFT | 5562 |  | Kiribati (Line Islands) | 209.983 | -9.586 | -0.0054 | 0.0015 | -3.5015 | 0.0005 | -10.403 | 391 |
| ALB | 5566 |  | Kiribati (Line Islands) | 211.472 | -9.572 | -0.0044 | 0.0012 | -3.5599 | 0.0004 | -10.842 | 1653 |
| ALB | 5604 |  | Kiribati (Line Islands) | 210.117 | -9.917 | -0.0061 | 0.0029 | -2.1070 | 0.0352 | -2.494 | 244 |
| YFT | 5620 | Vostok | Kiribati (Line Islands) | 207.610 | -10.100 | -0.0019 | 0.0012 | -1.6180 | 0.1057 | -0.659 | 822 |
| BET | 5638 |  | Kiribati (Line Islands) | 210.233 | -10.167 | -0.0054 | 0.0018 | -2.9971 | 0.0027 | -7.098 | 326 |
| BET | 5673 |  | Kiribati (Line Islands) | 205.833 | -10.450 | -0.0031 | 0.0011 | -2.7649 | 0.0057 | -5.817 | 931 |
| YFT | 5673 |  | Kiribati (Line Islands) | 205.833 | -10.450 | -0.0033 | 0.0012 | -2.7605 | 0.0058 | -5.792 | 931 |
| ALB | 5717 |  | Kiribati (Line Islands) | 211.433 | -10.750 | -0.0034 | 0.0017 | -2.0468 | 0.0407 | -2.246 | 680 |
| YFT | 5760 |  | Kiribati (Line Islands) | 207.967 | -11.083 | -0.0035 | 0.0022 | -1.5609 | 0.1186 | -0.492 | 243 |
| BET | 5779 |  | Kiribati (Line Islands) | 210.600 | -11.217 | -0.0036 | 0.0024 | -1.5170 | 0.1293 | -0.334 | 177 |
| BET | 5809 |  | Kiribati (Line Islands) | 207.833 | -11.417 | -0.0031 | 0.0018 | -1.7080 | 0.0877 | -0.992 | 301 |
| YFT | 5809 |  | Kiribati (Line Islands) | 207.833 | -11.417 | -0.0041 | 0.0019 | -2.1377 | 0.0326 | -2.686 | 301 |
| YFT | 5963 |  | Kiribati (Line Islands) | 209.150 | -12.829 | -0.0040 | 0.0019 | -2.0412 | 0.0413 | -2.244 | 347 |
| YFT | 4804 |  | Kiribati (Phoenix Islands) | 189.467 | -0.567 | -0.0071 | 0.0032 | -2.1994 | 0.0280 | -3.040 | 180 |
| YFT | 4906 |  | Kiribati (Phoenix Islands) | 185.383 | -1.767 | -0.0131 | 0.0017 | -7.6578 | 0.0000 | -57.514 | 480 |
| BET | 4941 |  | Kiribati (Phoenix Islands) | 189.900 | -2.350 | -0.0027 | 0.0015 | -1.7312 | 0.0835 | -1.036 | 683 |
| YFT | 4945 |  | Kiribati (Phoenix Islands) | 188.117 | -2.467 | -0.0040 | 0.0011 | -3.7031 | 0.0002 | -11.880 | 1277 |
| YFT | 4953 |  | Kiribati (Phoenix Islands) | 189.317 | -2.600 | -0.0023 | 0.0016 | -1.4953 | 0.1349 | -0.261 | 736 |
| YFT | 4979 |  | Kiribati (Phoenix Islands) | 188.700 | -2.983 | -0.0098 | 0.0036 | -2.7552 | 0.0059 | -5.671 | 346 |
| BET | 4994 |  | Kiribati (Phoenix Islands) | 191.783 | -3.200 | -0.0037 | 0.0020 | -1.7833 | 0.0746 | -1.230 | 401 |
| YFT | 4994 |  | Kiribati (Phoenix Islands) | 191.783 | -3.200 | -0.0035 | 0.0023 | -1.5331 | 0.1253 | -0.387 | 401 |
| YFT | 5032 |  | Kiribati (Phoenix Islands) | 189.333 | -3.683 | -0.0068 | 0.0019 | -3.6091 | 0.0003 | -11.151 | 655 |
| BET | 5033 |  | Kiribati (Phoenix Islands) | 188.067 | -3.700 | -0.0024 | 0.0014 | -1.7957 | 0.0726 | -1.261 | 569 |
| YFT | 5033 |  | Kiribati (Phoenix Islands) | 188.067 | -3.700 | -0.0032 | 0.0016 | -2.0540 | 0.0400 | -2.266 | 569 |
| ALB | 5043 | Tai | Kiribati (Phoenix Islands) | 186.735 | -3.878 | -0.0017 | 0.0011 | -1.5910 | 0.1117 | -0.560 | 1133 |
| BET | 5043 | Tai | Kiribati (Phoenix Islands) | 186.735 | -3.878 | -0.0013 | 0.0009 | -1.4745 | 0.1404 | -0.198 | 1133 |
| ALB | 5119 |  | Kiribati (Phoenix Islands) | 189.967 | -4.617 | -0.0025 | 0.0015 | -1.7425 | 0.0815 | -1.067 | 944 |
| ALB | 5167 |  | Kiribati (Phoenix Islands) | 190.517 | -5.100 | -0.0026 | 0.0014 | -1.9287 | 0.0538 | -1.756 | 1003 |
| ALB | 5169 |  | Kiribati (Phoenix Islands) | 184.433 | -5.183 | -0.0033 | 0.0014 | -2.3840 | 0.0171 | -3.746 | 787 |
| YFT | 5189 |  | Kiribati (Phoenix Islands) | 185.217 | -5.350 | -0.0024 | 0.0014 | -1.6901 | 0.0910 | -0.882 | 582 |
| YFT | 5234 | Fautasi | Kiribati (Phoenix Islands) | 185.887 | -5.821 | -0.0022 | 0.0014 | -1.5569 | 0.1195 | -0.448 | 611 |
| ALB | 3282 |  | Marshall Island | 173.233 | 12.250 | -0.0065 | 0.0012 | -5.4053 | 0.0000 | -28.337 | 650 |
| ALB | 3291 | Lawun-Pikaar | Marshall Island | 168.860 | 12.160 | -0.0100 | 0.0031 | -3.2443 | 0.0012 | -9.320 | 103 |
| ALB | 3300 |  | Marshall Island | 169.783 | 12.103 | -0.0057 | 0.0036 | -1.5713 | 0.1163 | -0.557 | 114 |
| ALB | 3367 |  | Marshall Island | 157.917 | 11.333 | -0.0047 | 0.0019 | -2.4011 | 0.0164 | -3.851 | 170 |
| BET | 3367 |  | Marshall Island | 157.917 | 11.333 | -0.0038 | 0.0026 | -1.4687 | 0.1420 | -0.190 | 170 |
| ALB | 3378 |  | Marshall Island | 159.150 | 11.242 | -0.0033 | 0.0021 | -1.5283 | 0.1265 | -0.382 | 311 |
| YFT | 3378 |  | Marshall Island | 159.150 | 11.242 | -0.0082 | 0.0031 | -2.6348 | 0.0085 | -5.074 | 311 |
| BET | 3401 | Lojemeja | Marshall Island | 161.090 | 11.440 | -0.0072 | 0.0041 | -1.7395 | 0.0820 | -1.074 | 110 |
| ALB | 3507 | Lewonjoui | Marshall Island | 166.533 | 10.583 | -0.0019 | 0.0011 | -1.8037 | 0.0714 | -1.325 | 471 |
| ALB | 3515 |  | Marshall Island | 162.717 | 10.533 | -0.0029 | 0.0019 | -1.5037 | 0.1327 | -0.288 | 375 |
| BET | 3515 |  | Marshall Island | 162.717 | 10.533 | -0.0033 | 0.0023 | -1.4282 | 0.1533 | -0.064 | 375 |
| ALB | 3554 |  | Marshall Island | 163.467 | 10.283 | -0.0026 | 0.0014 | -1.9168 | 0.0553 | -1.713 | 873 |
| BET | 3559 |  | Marshall Island | 162.017 | 10.233 | -0.0037 | 0.0021 | -1.7570 | 0.0790 | -1.122 | 501 |
| YFT | 3607 |  | Marshall Island | 170.683 | 9.983 | -0.0025 | 0.0015 | -1.6612 | 0.0968 | -0.799 | 505 |
| ALB | 3612 |  | Marshall Island | 164.333 | 9.917 | -0.0018 | 0.0008 | -2.3631 | 0.0182 | -3.642 | 1021 |
| BET | 3612 |  | Marshall Island | 164.333 | 9.917 | -0.0023 | 0.0009 | -2.5011 | 0.0124 | -4.320 | 1021 |
| BET | 3655 |  | Marshall Island | 162.967 | 9.583 | -0.0027 | 0.0018 | -1.5072 | 0.1318 | -0.296 | 401 |
| BET | 3701 |  | Marshall Island | 170.983 | 9.317 | -0.0022 | 0.0011 | -2.0556 | 0.0399 | -2.273 | 603 |
| YFT | 3701 |  | Marshall Island | 170.983 | 9.317 | -0.0048 | 0.0013 | -3.8223 | 0.0001 | -12.761 | 603 |
| ALB | 3703 | Randall | Marshall Island | 160.917 | 9.267 | -0.0032 | 0.0018 | -1.7496 | 0.0802 | -1.094 | 224 |
| YFT | 3796 |  | Marshall Island | 168.800 | 8.767 | -0.0032 | 0.0016 | -2.0142 | 0.0440 | -2.107 | 577 |
| BET | 3856 |  | Marshall Island | 161.717 | 8.394 | -0.0141 | 0.0054 | -2.5895 | 0.0096 | -4.786 | 111 |
| ALB | 3943 |  | Marshall Island | 175.367 | 7.917 | -0.0062 | 0.0010 | -6.3434 | 0.0000 | -39.023 | 147 |
| ALB | 3959 |  | Marshall Island | 169.483 | 7.800 | -0.0028 | 0.0013 | -2.0532 | 0.0401 | -2.251 | 225 |
| ALB | 3960 |  | Marshall Island | 169.033 | 7.783 | -0.0020 | 0.0008 | -2.4324 | 0.0150 | -3.958 | 144 |
| ALB | 3961 |  | Marshall Island | 171.633 | 7.783 | -0.0011 | 0.0004 | -2.7772 | 0.0055 | -5.798 | 484 |
| BET | 3961 |  | Marshall Island | 171.633 | 7.783 | -0.0019 | 0.0013 | -1.4664 | 0.1426 | -0.175 | 484 |
| BET | 3969 |  | Marshall Island | 175.083 | 7.733 | -0.0019 | 0.0013 | -1.4689 | 0.1420 | -0.204 | 509 |
| BET | 4043 |  | Marshall Island | 169.567 | 7.350 | -0.0023 | 0.0016 | -1.4260 | 0.1539 | -0.049 | 636 |
| YFT | 4074 |  | Marshall Island | 172.817 | 7.100 | -0.0064 | 0.0015 | -4.1490 | 0.0000 | -15.429 | 606 |
| YFT | 4220 | Keats Reef | Marshall Island | 173.480 | 5.900 | -0.0025 | 0.0014 | -1.7330 | 0.0832 | -1.043 | 661 |
| YFT | 4259 | Harrie / Limalok | Marshall Island | 172.360 | 5.600 | -0.0032 | 0.0020 | -1.6030 | 0.1090 | -0.598 | 256 |
| BET | 4297 |  | Marshall Island | 169.233 | 5.350 | -0.0024 | 0.0011 | -2.2910 | 0.0220 | -3.269 | 975 |
| ALB | 4421 |  | Marshall Island | 169.250 | 4.133 | -0.0003 | 0.0002 | -1.4692 | 0.1418 | -0.170 | 3148 |
| YFT | 13783 | Refractaire | New Caledonia | 167.735 | -23.918 | -0.0084 | 0.0037 | -2.2768 | 0.0230 | -3.525 | 103 |
| ALB | 6534 |  | New Caledonia | 163.317 | -16.983 | -0.0033 | 0.0023 | -1.4440 | 0.1489 | -0.163 | 190 |
| BET | 6605 |  | New Caledonia | 164.050 | -17.633 | -0.0039 | 0.0020 | -1.9971 | 0.0459 | -2.131 | 199 |
| YFT | 6714 |  | New Caledonia | 164.683 | -18.700 | -0.0126 | 0.0050 | -2.5354 | 0.0113 | -4.668 | 143 |
| ALB | 6756 |  | New Caledonia | 164.083 | -19.033 | -0.0074 | 0.0022 | -3.3720 | 0.0008 | -9.797 | 225 |
| BET | 6756 |  | New Caledonia | 164.083 | -19.033 | -0.0043 | 0.0018 | -2.4249 | 0.0154 | -4.113 | 225 |
| ALB | 6824 |  | New Caledonia | 164.850 | -19.900 | -0.0032 | 0.0019 | -1.6581 | 0.0976 | -0.887 | 349 |
| YFT | 6998 |  | New Caledonia | 168.634 | -22.040 | -0.0050 | 0.0019 | -2.6921 | 0.0071 | -5.405 | 725 |
| YFT | 7035 |  | New Caledonia | 168.717 | -22.330 | -0.0275 | 0.0050 | -5.5086 | 0.0000 | -29.004 | 116 |
| YFT | 7043 |  | New Caledonia | 169.000 | -22.367 | -0.0184 | 0.0060 | -3.0871 | 0.0020 | -7.774 | 120 |
| YFT | 7072 |  | New Caledonia | 159.705 | -22.701 | -0.0071 | 0.0019 | -3.8159 | 0.0001 | -12.802 | 487 |
| YFT | 7116 | Argo | New Caledonia | 159.550 | -23.167 | -0.0061 | 0.0026 | -2.3633 | 0.0182 | -3.684 | 339 |
| YFT | 7136 |  | New Caledonia | 159.805 | -23.395 | -0.0115 | 0.0023 | -4.8919 | 0.0000 | -22.281 | 315 |
| YFT | 7184 | Kelso | New Caledonia | 159.470 | -24.070 | -0.0059 | 0.0020 | -3.0057 | 0.0027 | -7.207 | 524 |
| BET | 8736 | Holopus | New Caledonia | 167.900 | -21.140 | -0.0028 | 0.0008 | -3.5442 | 0.0004 | -10.835 | 1290 |
| YFT | 8739 | Lansdowne | New Caledonia | 161.000 | -20.500 | -0.0066 | 0.0021 | -3.1179 | 0.0019 | -8.057 | 822 |
| ALB | 7508 | Colville | New Zealand | 180.600 | -26.117 | -0.0040 | 0.0017 | -2.3269 | 0.0200 | -3.568 | 302 |
| YFT | 7568 | Colville | New Zealand | 180.667 | -26.500 | -0.0081 | 0.0047 | -1.7059 | 0.0881 | -1.000 | 120 |
| ALB | 7803 | Kermadec | New Zealand | 181.983 | -29.500 | -0.0077 | 0.0034 | -2.2632 | 0.0239 | -3.549 | 109 |
| ALB | 7809 |  | New Zealand | 178.233 | -29.650 | -0.0048 | 0.0025 | -1.9047 | 0.0570 | -1.817 | 294 |
| BET | 7928 |  | New Zealand | 179.400 | -31.733 | -0.0019 | 0.0009 | -2.0911 | 0.0366 | -2.549 | 557 |
| BET | 7966 |  | New Zealand | 169.021 | -32.286 | -0.0049 | 0.0035 | -1.4120 | 0.1580 | -0.037 | 228 |
| BET | 8045 | Reinga | New Zealand | 170.000 | -33.333 | -0.0109 | 0.0042 | -2.5919 | 0.0096 | -4.841 | 575 |
| BET | 8103 |  | New Zealand | 172.750 | -33.833 | -0.0146 | 0.0017 | -8.3581 | 0.0000 | -68.087 | 1382 |
| ALB | 8144 |  | New Zealand | 179.570 | -34.530 | -0.0062 | 0.0020 | -3.1060 | 0.0019 | -7.848 | 151 |
| BET | 8144 |  | New Zealand | 179.570 | -34.530 | -0.0020 | 0.0012 | -1.6037 | 0.1088 | -0.627 | 151 |
| ALB | 8228 | Mokohinau | New Zealand | 176.283 | -35.667 | -0.0027 | 0.0009 | -2.8771 | 0.0040 | -6.327 | 2603 |
| YFT | 8228 | Mokohinau | New Zealand | 176.283 | -35.667 | -0.0012 | 0.0004 | -3.0639 | 0.0022 | -7.443 | 2603 |
| BET | 8343 |  | New Zealand | 181.060 | -37.468 | -0.0096 | 0.0040 | -2.3885 | 0.0169 | -3.740 | 649 |
| YFT | 8343 |  | New Zealand | 181.060 | -37.468 | -0.0079 | 0.0029 | -2.7498 | 0.0060 | -5.607 | 649 |
| BET | 8431 | Mahia | New Zealand | 179.283 | -39.685 | -0.0044 | 0.0018 | -2.3907 | 0.0168 | -3.738 | 2785 |
| YFT | 8978 | + Twin Peaks ridge | New Zealand | 178.615 | -37.391 | -0.0039 | 0.0024 | -1.6379 | 0.1015 | -0.691 | 4670 |
| YFT | 9142 |  | New Zealand | 182.038 | -27.086 | -0.0080 | 0.0027 | -2.9755 | 0.0030 | -7.207 | 258 |
| ALB | 9143 |  | New Zealand | 179.241 | -27.424 | -0.0032 | 0.0015 | -2.2046 | 0.0276 | -3.042 | 321 |
| BET | 9145 |  | New Zealand | 178.692 | -27.858 | -0.0051 | 0.0027 | -1.8733 | 0.0612 | -1.654 | 152 |
| YFT | 9145 |  | New Zealand | 178.692 | -27.858 | -0.0046 | 0.0030 | -1.5394 | 0.1239 | -0.468 | 152 |
| BET | 9168 |  | New Zealand | 174.085 | -32.809 | -0.0117 | 0.0032 | -3.6168 | 0.0003 | -11.171 | 1031 |
| ALB | 9175 |  | New Zealand | 175.841 | -34.468 | -0.0026 | 0.0012 | -2.2272 | 0.0260 | -2.990 | 3569 |
| BET | 9191 | Telecom Knoll | New Zealand | 170.672 | -36.942 | -0.0065 | 0.0036 | -1.8325 | 0.0679 | -1.795 | 104 |
| YFT | 9191 | Telecom Knoll | New Zealand | 170.672 | -36.942 | -0.0041 | 0.0027 | -1.5150 | 0.1309 | -0.598 | 104 |
| BET | 9210 |  | New Zealand | 178.243 | -40.278 | -0.0050 | 0.0024 | -2.0726 | 0.0382 | -2.316 | 1639 |
| BET | 9221 |  | New Zealand | 178.484 | -41.992 | -0.0024 | 0.0010 | -2.3255 | 0.0201 | -3.458 | 131 |
| BET | 6535 |  | Niue | 189.083 | -17.000 | -0.0020 | 0.0011 | -1.7520 | 0.0799 | -1.170 | 736 |
| YFT | 6535 |  | Niue | 189.083 | -17.000 | -0.0021 | 0.0014 | -1.4504 | 0.1471 | -0.173 | 736 |
| BET | 6667 | Antiope reef | Niue | 191.609 | -18.261 | -0.0081 | 0.0022 | -3.7677 | 0.0002 | -12.809 | 162 |
| YFT | 6667 | Antiope reef | Niue | 191.609 | -18.261 | -0.0240 | 0.0030 | -8.1256 | 0.0000 | -65.816 | 162 |
| YFT | 6767 | Lachlan | Niue | 190.467 | -19.233 | -0.0033 | 0.0022 | -1.5212 | 0.1285 | -0.435 | 280 |
| ALB | 6776 |  | Niue | 192.350 | -19.354 | -0.0036 | 0.0025 | -1.3963 | 0.1630 | -0.125 | 107 |
| BET | 6842 |  | Niue | 191.250 | -20.140 | -0.0077 | 0.0030 | -2.6004 | 0.0095 | -5.211 | 120 |
| ALB | 7818 |  | Norfolk Island | 169.183 | -29.767 | -0.0051 | 0.0019 | -2.6928 | 0.0072 | -5.563 | 333 |
| BET | 8786 |  | Norfolk Island | 169.040 | -26.999 | -0.0036 | 0.0023 | -1.5494 | 0.1216 | -0.588 | 134 |
| YFT | 8786 |  | Norfolk Island | 169.040 | -26.999 | -0.0040 | 0.0027 | -1.4696 | 0.1420 | -0.329 | 134 |
| ALB | 8787 |  | Norfolk Island | 170.754 | -27.639 | -0.0085 | 0.0026 | -3.2835 | 0.0011 | -9.361 | 114 |
| ALB | 3985 |  | Palau | 135.333 | 7.617 | -0.0048 | 0.0017 | -2.7809 | 0.0054 | -5.790 | 203 |
| YFT | 4066 |  | Palau | 135.733 | 7.183 | -0.0060 | 0.0028 | -2.0951 | 0.0362 | -2.418 | 388 |
| ALB | 4240 |  | Palau | 133.983 | 5.733 | -0.0009 | 0.0002 | -4.3116 | 0.0000 | -16.677 | 1054 |
| YFT | 4268 |  | Palau | 131.267 | 5.583 | -0.0025 | 0.0016 | -1.5184 | 0.1290 | -0.369 | 397 |
| ALB | 4390 |  | Palau | 131.017 | 4.467 | -0.0017 | 0.0011 | -1.4652 | 0.1429 | -0.196 | 106 |
| BET | 4439 |  | Palau | 130.583 | 3.933 | -0.0030 | 0.0021 | -1.4307 | 0.1526 | -0.097 | 322 |
| YFT | 4376 |  | Palmyra | 198.317 | 4.633 | -0.0041 | 0.0020 | -2.0681 | 0.0387 | -2.372 | 485 |
| BET | 4521 | Mussau | Papua New Guinea | 148.950 | 2.767 | -0.0014 | 0.0009 | -1.4471 | 0.1479 | -0.105 | 1292 |
| BET | 4641 |  | Papua New Guinea | 151.050 | 1.467 | -0.0046 | 0.0024 | -1.8859 | 0.0594 | -1.611 | 173 |
| YFT | 4781 | Mussau | Papua New Guinea | 149.550 | -0.200 | -0.0065 | 0.0026 | -2.5590 | 0.0106 | -4.726 | 195 |
| ALB | 4843 |  | Papua New Guinea | 147.900 | -1.017 | -0.0023 | 0.0005 | -4.3340 | 0.0000 | -17.133 | 560 |
| YFT | 4848 |  | Papua New Guinea | 151.850 | -1.100 | -0.0031 | 0.0013 | -2.3417 | 0.0193 | -3.599 | 665 |
| BET | 4852 |  | Papua New Guinea | 143.033 | -1.167 | -0.0044 | 0.0026 | -1.7408 | 0.0819 | -1.097 | 173 |
| YFT | 4852 |  | Papua New Guinea | 143.033 | -1.167 | -0.0045 | 0.0024 | -1.9009 | 0.0575 | -1.692 | 173 |
| BET | 4853 |  | Papua New Guinea | 148.250 | -1.167 | -0.0025 | 0.0017 | -1.4329 | 0.1520 | -0.093 | 415 |
| BET | 4895 | Lyra | Papua New Guinea | 153.420 | -1.758 | -0.0050 | 0.0018 | -2.8234 | 0.0048 | -6.111 | 510 |
| BET | 4925 |  | Papua New Guinea | 142.383 | -2.000 | -0.0043 | 0.0020 | -2.1735 | 0.0299 | -2.826 | 238 |
| ALB | 4939 |  | Papua New Guinea | 152.083 | -2.333 | -0.0013 | 0.0007 | -1.9151 | 0.0556 | -1.728 | 509 |
| ALB | 4942 |  | Papua New Guinea | 152.800 | -2.350 | -0.0021 | 0.0010 | -2.0404 | 0.0414 | -2.232 | 359 |
| BET | 5002 | Sherburne Reef | Papua New Guinea | 148.021 | -3.300 | -0.0028 | 0.0019 | -1.4611 | 0.1441 | -0.157 | 464 |
| ALB | 5031 |  | Papua New Guinea | 154.517 | -3.667 | -0.0034 | 0.0014 | -2.3435 | 0.0192 | -3.620 | 322 |
| ALB | 5073 |  | Papua New Guinea | 154.533 | -4.217 | -0.0042 | 0.0016 | -2.6659 | 0.0077 | -5.277 | 302 |
| ALB | 5133 |  | Papua New Guinea | 154.417 | -4.717 | -0.0070 | 0.0022 | -3.2100 | 0.0013 | -8.561 | 256 |
| ALB | 5154 |  | Papua New Guinea | 153.083 | -5.000 | -0.0073 | 0.0024 | -3.0515 | 0.0023 | -7.536 | 184 |
| ALB | 5296 |  | Papua New Guinea | 148.333 | -6.733 | -0.0017 | 0.0008 | -2.2290 | 0.0259 | -3.066 | 987 |
| BET | 5503 |  | Papua New Guinea | 153.633 | -8.967 | -0.0071 | 0.0038 | -1.8559 | 0.0635 | -1.493 | 257 |
| ALB | 5629 | Portlock | Papua New Guinea | 145.590 | -10.083 | -0.0028 | 0.0013 | -2.1525 | 0.0315 | -2.726 | 1406 |
| ALB | 5636 |  | Papua New Guinea | 155.483 | -10.133 | -0.0072 | 0.0033 | -2.1424 | 0.0322 | -2.665 | 151 |
| YFT | 5688 | Plibersek | Papua New Guinea | 153.767 | -10.550 | -0.0023 | 0.0013 | -1.7509 | 0.0800 | -1.104 | 939 |
| YFT | 5982 |  | Papua New Guinea | 153.850 | -12.833 | -0.0020 | 0.0010 | -1.9134 | 0.0558 | -1.738 | 1139 |
| YFT | 8705 | Dyaul | Papua New Guinea | 150.983 | -3.250 | -0.0059 | 0.0023 | -2.5277 | 0.0115 | -4.495 | 213 |
| BET | 8734 |  | Papua New Guinea | 151.180 | -4.157 | -0.0032 | 0.0010 | -3.2976 | 0.0010 | -9.105 | 884 |
| YFT | 9229 | Moresby | Papua New Guinea | 151.567 | -9.817 | -0.0051 | 0.0014 | -3.6916 | 0.0002 | -11.900 | 626 |
| ALB | 9231 | Papua | Papua New Guinea | 146.000 | -10.750 | -0.0025 | 0.0013 | -1.9384 | 0.0527 | -1.833 | 933 |
| YFT | 9231 | Papua | Papua New Guinea | 146.000 | -10.750 | -0.0022 | 0.0013 | -1.7404 | 0.0819 | -1.090 | 933 |
| YFT | 9311 |  | Papua New Guinea | 153.445 | -9.264 | -0.0087 | 0.0041 | -2.1083 | 0.0350 | -2.504 | 145 |
| BET | 9312 |  | Papua New Guinea | 153.237 | -9.245 | -0.0056 | 0.0014 | -3.9956 | 0.0001 | -14.156 | 780 |
| YFT | 9312 |  | Papua New Guinea | 153.237 | -9.245 | -0.0023 | 0.0014 | -1.6445 | 0.1001 | -0.740 | 780 |
| ALB | 6916 |  | Pitcairn Islands | 229.500 | -20.817 | -0.0032 | 0.0011 | -3.0674 | 0.0022 | -7.877 | 403 |
| ALB | 6926 |  | Pitcairn Islands | 230.400 | -21.050 | -0.0025 | 0.0014 | -1.7360 | 0.0829 | -1.205 | 297 |
| ALB | 6042 | Si'usi'u | Samoa | 186.420 | -13.217 | -0.0038 | 0.0020 | -1.8854 | 0.0594 | -1.596 | 497 |
| BET | 6042 | Si'usi'u | Samoa | 186.420 | -13.217 | -0.0088 | 0.0017 | -5.2205 | 0.0000 | -25.530 | 497 |
| BET | 6145 |  | Samoa | 187.367 | -14.217 | -0.0030 | 0.0018 | -1.6497 | 0.0990 | -0.742 | 204 |
| YFT | 8591 | Taumatau | Samoa | 187.749 | -13.257 | -0.0050 | 0.0017 | -2.9368 | 0.0033 | -6.688 | 1114 |
| BET | 5312 |  | Solomon Islands | 159.150 | -6.967 | -0.0013 | 0.0007 | -1.8620 | 0.0626 | -1.495 | 3039 |
| ALB | 5331 |  | Solomon Islands | 163.367 | -7.183 | -0.0068 | 0.0021 | -3.1577 | 0.0016 | -8.277 | 553 |
| YFT | 5402 |  | Solomon Islands | 160.167 | -7.917 | -0.0027 | 0.0017 | -1.5746 | 0.1154 | -0.502 | 942 |
| YFT | 5450 |  | Solomon Islands | 164.700 | -8.383 | -0.0045 | 0.0019 | -2.3069 | 0.0211 | -3.473 | 348 |
| ALB | 5460 |  | Solomon Islands | 167.933 | -8.517 | -0.0079 | 0.0039 | -2.0297 | 0.0425 | -2.286 | 180 |
| YFT | 5466 |  | Solomon Islands | 170.700 | -8.583 | -0.0062 | 0.0031 | -2.0259 | 0.0428 | -2.201 | 134 |
| ALB | 5504 |  | Solomon Islands | 170.183 | -8.967 | -0.0082 | 0.0032 | -2.5746 | 0.0101 | -4.819 | 158 |
| ALB | 5530 |  | Solomon Islands | 156.067 | -9.283 | -0.0090 | 0.0025 | -3.5834 | 0.0003 | -11.059 | 289 |
| BET | 5530 |  | Solomon Islands | 156.067 | -9.283 | -0.0048 | 0.0023 | -2.0635 | 0.0391 | -2.334 | 289 |
| YFT | 5530 |  | Solomon Islands | 156.067 | -9.283 | -0.0083 | 0.0023 | -3.6000 | 0.0003 | -11.180 | 289 |
| YFT | 5548 |  | Solomon Islands | 162.550 | -9.400 | -0.0022 | 0.0016 | -1.4252 | 0.1542 | -0.067 | 668 |
| BET | 5586 |  | Solomon Islands | 158.767 | -9.717 | -0.0049 | 0.0026 | -1.8823 | 0.0599 | -1.588 | 378 |
| ALB | 5611 |  | Solomon Islands | 157.000 | -9.983 | -0.0067 | 0.0034 | -2.0104 | 0.0444 | -2.103 | 397 |
| BET | 5626 |  | Solomon Islands | 159.033 | -10.067 | -0.0046 | 0.0021 | -2.2153 | 0.0268 | -2.967 | 689 |
| YFT | 5626 |  | Solomon Islands | 159.033 | -10.067 | -0.0053 | 0.0022 | -2.3788 | 0.0174 | -3.727 | 689 |
| ALB | 5678 |  | Solomon Islands | 162.967 | -10.483 | -0.0099 | 0.0030 | -3.2536 | 0.0011 | -8.734 | 516 |
| BET | 5680 | Hammondsport | Solomon Islands | 159.583 | -10.533 | -0.0029 | 0.0018 | -1.6774 | 0.0935 | -0.851 | 712 |
| YFT | 5742 |  | Solomon Islands | 159.967 | -10.917 | -0.0057 | 0.0031 | -1.8542 | 0.0638 | -1.487 | 136 |
| YFT | 5790 |  | Solomon Islands | 168.500 | -11.250 | -0.0055 | 0.0030 | -1.8391 | 0.0660 | -1.524 | 194 |
| YFT | 5791 |  | Solomon Islands | 164.233 | -11.267 | -0.0029 | 0.0014 | -2.0934 | 0.0364 | -2.459 | 544 |
| ALB | 5808 |  | Solomon Islands | 169.617 | -11.400 | -0.0067 | 0.0033 | -1.9885 | 0.0468 | -2.070 | 157 |
| YFT | 5808 |  | Solomon Islands | 169.617 | -11.400 | -0.0076 | 0.0040 | -1.8930 | 0.0584 | -1.689 | 157 |
| YFT | 5894 |  | Solomon Islands | 171.533 | -12.283 | -0.0094 | 0.0062 | -1.5122 | 0.1306 | -0.332 | 117 |
| BET | 5928 |  | Solomon Islands | 170.106 | -12.506 | -0.0026 | 0.0016 | -1.6172 | 0.1059 | -0.672 | 426 |
| YFT | 5933 |  | Solomon Islands | 168.200 | -12.517 | -0.0069 | 0.0040 | -1.7100 | 0.0874 | -1.012 | 177 |
| BET | 6008 |  | Solomon Islands | 171.667 | -12.967 | -0.0074 | 0.0050 | -1.4667 | 0.1425 | -0.192 | 102 |
| YFT | 6008 |  | Solomon Islands | 171.667 | -12.967 | -0.0222 | 0.0063 | -3.5075 | 0.0005 | -10.527 | 102 |
| YFT | 6017 |  | Solomon Islands | 172.667 | -13.083 | -0.0036 | 0.0022 | -1.6462 | 0.0998 | -0.752 | 340 |
| BET | 6024 |  | Solomon Islands | 158.133 | -13.117 | -0.0014 | 0.0009 | -1.4977 | 0.1343 | -0.289 | 1501 |
| BET | 6025 |  | Solomon Islands | 162.233 | -13.117 | -0.0018 | 0.0008 | -2.2047 | 0.0275 | -2.915 | 1854 |
| ALB | 6115 |  | Solomon Islands | 160.350 | -13.867 | -0.0088 | 0.0023 | -3.7829 | 0.0002 | -12.439 | 361 |
| YFT | 6129 |  | Solomon Islands | 170.917 | -14.050 | -0.0043 | 0.0010 | -4.1052 | 0.0000 | -15.142 | 1571 |
| BET | 6148 |  | Solomon Islands | 161.283 | -14.217 | -0.0034 | 0.0019 | -1.8333 | 0.0668 | -1.397 | 230 |
| YFT | 6148 |  | Solomon Islands | 161.283 | -14.217 | -0.0033 | 0.0022 | -1.5074 | 0.1318 | -0.297 | 230 |
| ALB | 6166 |  | Solomon Islands | 161.100 | -14.317 | -0.0102 | 0.0070 | -1.4659 | 0.1427 | -0.172 | 113 |
| YFT | 6209 |  | Solomon Islands | 159.550 | -14.533 | -0.0043 | 0.0016 | -2.5843 | 0.0098 | -4.761 | 494 |
| YFT | 6223 |  | Solomon Islands | 160.767 | -14.617 | -0.0035 | 0.0014 | -2.4662 | 0.0137 | -4.148 | 676 |
| BET | 8588 |  | Solomon Islands | 158.030 | -8.920 | -0.0114 | 0.0046 | -2.4812 | 0.0131 | -4.236 | 133 |
| BET | 10104 |  | South Pacific RFMO High seas | 240.217 | -16.935 | -0.0040 | 0.0028 | -1.4197 | 0.1561 | -0.157 | 108 |
| YFT | 10104 |  | South Pacific RFMO High seas | 240.217 | -16.935 | -0.0057 | 0.0024 | -2.3664 | 0.0182 | -3.980 | 108 |
| BET | 10115 | Taipaka | South Pacific RFMO High seas | 242.628 | -17.804 | -0.0066 | 0.0026 | -2.5451 | 0.0111 | -4.794 | 163 |
| ALB | 10123 | Apitoka | South Pacific RFMO High seas | 242.844 | -18.686 | -0.0020 | 0.0014 | -1.3895 | 0.1650 | -0.039 | 343 |
| BET | 10129 |  | South Pacific RFMO High seas | 239.999 | -20.121 | -0.0022 | 0.0012 | -1.8457 | 0.0653 | -1.613 | 690 |
| ALB | 13554 | Standard | South Pacific RFMO High seas | 157.783 | -35.017 | -0.0036 | 0.0010 | -3.4048 | 0.0007 | -9.713 | 1111 |
| ALB | 13636 |  | South Pacific RFMO High seas | 188.528 | -25.327 | -0.0018 | 0.0012 | -1.5121 | 0.1306 | -0.356 | 346 |
| YFT | 13636 |  | South Pacific RFMO High seas | 188.528 | -25.327 | -0.0026 | 0.0016 | -1.5811 | 0.1140 | -0.575 | 346 |
| BET | 13637 |  | South Pacific RFMO High seas | 190.137 | -25.564 | -0.0041 | 0.0028 | -1.5033 | 0.1329 | -0.335 | 117 |
| YFT | 13649 |  | South Pacific RFMO High seas | 191.069 | -28.964 | -0.0041 | 0.0023 | -1.7321 | 0.0834 | -1.101 | 185 |
| ALB | 4859 |  | South Pacific RFMO High seas | 215.515 | -1.302 | -0.0021 | 0.0013 | -1.5870 | 0.1137 | -0.815 | 280 |
| ALB | 5083 |  | South Pacific RFMO High seas | 216.967 | -4.371 | -0.0021 | 0.0013 | -1.6185 | 0.1066 | -1.003 | 247 |
| ALB | 5146 |  | South Pacific RFMO High seas | 214.950 | -4.950 | -0.0041 | 0.0019 | -2.1325 | 0.0334 | -2.937 | 183 |
| ALB | 5295 |  | South Pacific RFMO High seas | 207.733 | -6.717 | -0.0028 | 0.0017 | -1.6478 | 0.0994 | -0.745 | 540 |
| BET | 5295 |  | South Pacific RFMO High seas | 207.733 | -6.717 | -0.0034 | 0.0012 | -2.8507 | 0.0044 | -6.211 | 540 |
| BET | 5555 |  | South Pacific RFMO High seas | 213.259 | -9.561 | -0.0022 | 0.0011 | -2.0867 | 0.0370 | -2.441 | 991 |
| BET | 5585 |  | South Pacific RFMO High seas | 213.517 | -9.752 | -0.0018 | 0.0010 | -1.7827 | 0.0747 | -1.248 | 1089 |
| YFT | 5804 |  | South Pacific RFMO High seas | 229.495 | -11.423 | -0.0042 | 0.0019 | -2.1680 | 0.0306 | -3.172 | 353 |
| ALB | 6038 |  | South Pacific RFMO High seas | 230.429 | -13.222 | -0.0038 | 0.0019 | -2.0108 | 0.0446 | -2.268 | 280 |
| ALB | 6105 |  | South Pacific RFMO High seas | 225.161 | -13.825 | -0.0021 | 0.0013 | -1.6361 | 0.1020 | -0.796 | 423 |
| ALB | 6319 |  | South Pacific RFMO High seas | 228.583 | -15.511 | -0.0022 | 0.0014 | -1.5935 | 0.1112 | -0.636 | 396 |
| ALB | 6325 |  | South Pacific RFMO High seas | 229.469 | -15.608 | -0.0034 | 0.0015 | -2.2804 | 0.0227 | -3.393 | 345 |
| YFT | 6325 |  | South Pacific RFMO High seas | 229.469 | -15.608 | -0.0027 | 0.0017 | -1.6412 | 0.1009 | -0.795 | 345 |
| BET | 6429 |  | South Pacific RFMO High seas | 234.550 | -16.439 | -0.0030 | 0.0021 | -1.4327 | 0.1521 | -0.132 | 208 |
| YFT | 6473 |  | South Pacific RFMO High seas | 237.833 | -16.654 | -0.0030 | 0.0015 | -2.0278 | 0.0429 | -2.411 | 278 |
| BET | 6659 |  | South Pacific RFMO High seas | 235.733 | -18.178 | -0.0041 | 0.0011 | -3.7706 | 0.0002 | -12.605 | 801 |
| YFT | 6659 |  | South Pacific RFMO High seas | 235.733 | -18.178 | -0.0058 | 0.0010 | -5.7278 | 0.0000 | -31.569 | 801 |
| BET | 6745 |  | South Pacific RFMO High seas | 233.883 | -18.956 | -0.0017 | 0.0010 | -1.6749 | 0.0942 | -0.927 | 908 |
| ALB | 6757 |  | South Pacific RFMO High seas | 236.368 | -19.079 | -0.0013 | 0.0009 | -1.5169 | 0.1295 | -0.377 | 938 |
| YFT | 6757 |  | South Pacific RFMO High seas | 236.368 | -19.079 | -0.0033 | 0.0011 | -3.0969 | 0.0020 | -7.886 | 938 |
| ALB | 7205 |  | South Pacific RFMO High seas | 179.417 | -24.133 | -0.0031 | 0.0011 | -2.7625 | 0.0058 | -5.828 | 589 |
| YFT | 7297 |  | South Pacific RFMO High seas | 198.317 | -24.878 | -0.0050 | 0.0026 | -1.8915 | 0.0590 | -1.932 | 117 |
| BET | 7359 |  | South Pacific RFMO High seas | 178.933 | -25.283 | -0.0015 | 0.0009 | -1.6789 | 0.0933 | -0.882 | 819 |
| ALB | 7376 |  | South Pacific RFMO High seas | 190.417 | -25.408 | -0.0037 | 0.0023 | -1.6123 | 0.1070 | -0.694 | 104 |
| BET | 7376 |  | South Pacific RFMO High seas | 190.417 | -25.408 | -0.0048 | 0.0028 | -1.6976 | 0.0897 | -0.986 | 104 |
| YFT | 7532 |  | South Pacific RFMO High seas | 172.600 | -26.233 | -0.0048 | 0.0031 | -1.5508 | 0.1211 | -0.487 | 239 |
| BET | 7557 | Sonne 167 Volcano 32 | South Pacific RFMO High seas | 185.280 | -26.450 | -0.0031 | 0.0016 | -1.9633 | 0.0497 | -1.940 | 284 |
| YFT | 7557 | Sonne 167 Volcano 32 | South Pacific RFMO High seas | 185.280 | -26.450 | -0.0028 | 0.0018 | -1.5443 | 0.1226 | -0.438 | 284 |
| YFT | 7587 | Gifford / North Tasman Seamount No. 5A | South Pacific RFMO High seas | 159.483 | -26.700 | -0.0043 | 0.0008 | -5.4574 | 0.0000 | -27.988 | 1359 |
| YFT | 7623 | North Tasman Seamount | South Pacific RFMO High seas | 159.333 | -26.983 | -0.0028 | 0.0006 | -4.3200 | 0.0000 | -16.785 | 2068 |
| ALB | 7733 |  | South Pacific RFMO High seas | 186.400 | -28.150 | -0.0014 | 0.0010 | -1.4878 | 0.1369 | -0.267 | 324 |
| ALB | 7734 |  | South Pacific RFMO High seas | 189.436 | -28.221 | -0.0035 | 0.0020 | -1.7810 | 0.0750 | -1.255 | 159 |
| BET | 7752 |  | South Pacific RFMO High seas | 208.150 | -28.491 | -0.0030 | 0.0019 | -1.6219 | 0.1052 | -0.822 | 203 |
| ALB | 7765 | Sonne 167 Volcano 34 | South Pacific RFMO High seas | 186.410 | -28.660 | -0.0062 | 0.0021 | -2.9527 | 0.0032 | -6.962 | 117 |
| YFT | 7827 |  | South Pacific RFMO High seas | 219.350 | -29.950 | -0.0036 | 0.0023 | -1.5876 | 0.1127 | -0.651 | 205 |
| BET | 7828 |  | South Pacific RFMO High seas | 221.255 | -29.998 | -0.0018 | 0.0013 | -1.4569 | 0.1456 | -0.258 | 465 |
| YFT | 7828 |  | South Pacific RFMO High seas | 221.255 | -29.998 | -0.0020 | 0.0014 | -1.4899 | 0.1367 | -0.362 | 465 |
| ALB | 7921 |  | South Pacific RFMO High seas | 196.950 | -31.656 | -0.0052 | 0.0018 | -2.8248 | 0.0049 | -6.779 | 174 |
| BET | 8199 |  | South Pacific RFMO High seas | 156.150 | -35.317 | -0.0018 | 0.0010 | -1.8782 | 0.0604 | -1.565 | 951 |
| BET | 8225 |  | South Pacific RFMO High seas | 213.317 | -35.700 | -0.0073 | 0.0025 | -2.8529 | 0.0046 | -7.178 | 163 |
| YFT | 8225 |  | South Pacific RFMO High seas | 213.317 | -35.700 | -0.0049 | 0.0025 | -1.9685 | 0.0499 | -2.400 | 163 |
| ALB | 8248 |  | South Pacific RFMO High seas | 214.952 | -36.245 | -0.0030 | 0.0021 | -1.4075 | 0.1606 | -0.300 | 148 |
| YFT | 8280 | Gascoyne | South Pacific RFMO High seas | 156.167 | -36.650 | -0.0032 | 0.0014 | -2.2945 | 0.0218 | -3.334 | 488 |
| BET | 8284 |  | South Pacific RFMO High seas | 155.717 | -36.683 | -0.0018 | 0.0008 | -2.2845 | 0.0224 | -3.289 | 1195 |
| YFT | 8284 |  | South Pacific RFMO High seas | 155.717 | -36.683 | -0.0018 | 0.0009 | -1.9821 | 0.0475 | -1.982 | 1195 |
| YFT | 8650 | Capel | South Pacific RFMO High seas | 159.717 | -25.000 | -0.0059 | 0.0015 | -4.0013 | 0.0001 | -14.266 | 913 |
| BET | 9281 |  | South Pacific RFMO High seas | 172.979 | -26.607 | -0.0039 | 0.0025 | -1.5575 | 0.1195 | -0.499 | 171 |
| YFT | 9281 |  | South Pacific RFMO High seas | 172.979 | -26.607 | -0.0059 | 0.0029 | -2.0314 | 0.0423 | -2.251 | 171 |
| YFT | 9283 |  | South Pacific RFMO High seas | 171.927 | -26.700 | -0.0030 | 0.0016 | -1.8469 | 0.0649 | -1.531 | 348 |
| ALB | 5323 |  | Tokelau | 188.417 | -7.100 | -0.0038 | 0.0010 | -3.7884 | 0.0002 | -12.507 | 1196 |
| ALB | 5359 |  | Tokelau | 187.417 | -7.500 | -0.0051 | 0.0034 | -1.5014 | 0.1333 | -0.280 | 228 |
| BET | 5359 |  | Tokelau | 187.417 | -7.500 | -0.0069 | 0.0035 | -1.9821 | 0.0475 | -1.974 | 228 |
| BET | 5425 | Pogisa | Tokelau | 187.337 | -8.152 | -0.0057 | 0.0026 | -2.2392 | 0.0252 | -3.073 | 224 |
| BET | 5438 |  | Tokelau | 186.933 | -8.250 | -0.0035 | 0.0022 | -1.5579 | 0.1193 | -0.456 | 321 |
| BET | 5516 |  | Tokelau | 185.933 | -9.117 | -0.0063 | 0.0018 | -3.4375 | 0.0006 | -9.948 | 403 |
| ALB | 5553 |  | Tokelau | 188.017 | -9.500 | -0.0053 | 0.0013 | -4.0131 | 0.0001 | -14.311 | 473 |
| ALB | 5590 |  | Tokelau | 191.318 | -9.744 | -0.0036 | 0.0018 | -1.9624 | 0.0497 | -1.891 | 446 |
| YFT | 5590 |  | Tokelau | 191.318 | -9.744 | -0.0069 | 0.0023 | -2.9846 | 0.0028 | -6.998 | 446 |
| BET | 6183 |  | Tonga | 184.039 | -14.428 | -0.0049 | 0.0027 | -1.7721 | 0.0765 | -1.257 | 211 |
| ALB | 6194 |  | Tonga | 184.383 | -14.467 | -0.0052 | 0.0025 | -2.0800 | 0.0376 | -2.475 | 176 |
| BET | 6278 |  | Tonga | 186.667 | -15.017 | -0.0035 | 0.0024 | -1.4406 | 0.1497 | -0.114 | 232 |
| ALB | 6301 |  | Tonga | 186.483 | -15.300 | -0.0034 | 0.0015 | -2.3268 | 0.0200 | -3.518 | 593 |
| BET | 6336 |  | Tonga | 187.017 | -15.633 | -0.0049 | 0.0012 | -4.1237 | 0.0000 | -15.328 | 874 |
| YFT | 6336 |  | Tonga | 187.017 | -15.633 | -0.0073 | 0.0016 | -4.6190 | 0.0000 | -19.730 | 874 |
| ALB | 6402 |  | Tonga | 183.733 | -16.117 | -0.0158 | 0.0047 | -3.3932 | 0.0007 | -9.766 | 129 |
| BET | 6402 |  | Tonga | 183.733 | -16.117 | -0.0156 | 0.0050 | -3.1097 | 0.0019 | -7.885 | 129 |
| ALB | 6470 |  | Tonga | 184.100 | -16.617 | -0.0046 | 0.0030 | -1.5215 | 0.1282 | -0.364 | 197 |
| BET | 6553 |  | Tonga | 185.317 | -17.200 | -0.0037 | 0.0014 | -2.5925 | 0.0096 | -4.842 | 544 |
| YFT | 6553 |  | Tonga | 185.317 | -17.200 | -0.0028 | 0.0017 | -1.6636 | 0.0963 | -0.818 | 544 |
| YFT | 6554 |  | Tonga | 186.083 | -17.200 | -0.0039 | 0.0020 | -1.9982 | 0.0458 | -2.059 | 403 |
| YFT | 6600 |  | Tonga | 185.283 | -17.600 | -0.0086 | 0.0035 | -2.4762 | 0.0133 | -4.232 | 307 |
| BET | 6612 |  | Tonga | 185.383 | -17.733 | -0.0036 | 0.0021 | -1.7007 | 0.0891 | -0.940 | 290 |
| YFT | 6612 |  | Tonga | 185.383 | -17.733 | -0.0049 | 0.0025 | -1.9482 | 0.0514 | -1.858 | 290 |
| BET | 6706 | Capricorn | Tonga | 187.800 | -18.600 | -0.0093 | 0.0011 | -8.1685 | 0.0000 | -65.943 | 620 |
| YFT | 6706 | Capricorn | Tonga | 187.800 | -18.600 | -0.0128 | 0.0014 | -9.1616 | 0.0000 | -83.226 | 620 |
| YFT | 6750 | Home Reef | Tonga | 185.225 | -18.992 | -0.0085 | 0.0030 | -2.8677 | 0.0041 | -6.326 | 280 |
| ALB | 6850 |  | Tonga | 186.033 | -20.150 | -0.0180 | 0.0054 | -3.3328 | 0.0009 | -9.276 | 183 |
| BET | 6856 | Falcon / Fonuafo'ou | Tonga | 184.580 | -20.320 | -0.0023 | 0.0012 | -1.9166 | 0.0553 | -1.719 | 679 |
| YFT | 6856 | Falcon / Fonuafo'ou | Tonga | 184.580 | -20.320 | -0.0081 | 0.0014 | -5.8101 | 0.0000 | -32.114 | 679 |
| YFT | 6876 |  | Tonga | 186.467 | -20.450 | -0.0062 | 0.0017 | -3.6677 | 0.0002 | -11.714 | 460 |
| BET | 6883 |  | Tonga | 184.517 | -20.533 | -0.0021 | 0.0014 | -1.4705 | 0.1415 | -0.190 | 498 |
| YFT | 6883 |  | Tonga | 184.517 | -20.533 | -0.0038 | 0.0017 | -2.2401 | 0.0251 | -3.081 | 498 |
| BET | 6889 |  | Tonga | 185.900 | -20.583 | -0.0018 | 0.0010 | -1.7806 | 0.0750 | -1.218 | 947 |
| YFT | 6889 |  | Tonga | 185.900 | -20.583 | -0.0025 | 0.0012 | -2.1058 | 0.0353 | -2.500 | 947 |
| YFT | 6929 |  | Tonga | 184.333 | -21.100 | -0.0035 | 0.0018 | -1.8944 | 0.0582 | -1.634 | 588 |
| ALB | 6967 |  | Tonga | 183.050 | -21.300 | -0.0049 | 0.0032 | -1.5488 | 0.1215 | -0.441 | 258 |
| YFT | 7000 |  | Tonga | 183.850 | -22.033 | -0.0082 | 0.0041 | -2.0023 | 0.0453 | -2.079 | 258 |
| YFT | 7059 |  | Tonga | 183.667 | -22.550 | -0.0125 | 0.0067 | -1.8661 | 0.0621 | -1.560 | 110 |
| YFT | 7237 |  | Tonga | 184.450 | -24.500 | -0.0052 | 0.0019 | -2.6977 | 0.0070 | -5.608 | 360 |
| BET | 8617 | Bicentenary | Tonga | 184.577 | -19.427 | -0.0034 | 0.0009 | -3.8743 | 0.0001 | -13.196 | 1313 |
| YFT | 8617 | Bicentenary | Tonga | 184.577 | -19.427 | -0.0036 | 0.0011 | -3.3781 | 0.0007 | -9.555 | 1313 |
| YFT | 8619 |  | Tonga | 184.470 | -20.850 | -0.0061 | 0.0024 | -2.5229 | 0.0117 | -4.440 | 332 |
| YFT | 8750 |  | Tonga | 185.850 | -17.550 | -0.0100 | 0.0028 | -3.5192 | 0.0004 | -10.587 | 201 |
| ALB | 5081 |  | Tuvalu | 176.650 | -4.333 | -0.0083 | 0.0042 | -1.9562 | 0.0505 | -1.874 | 254 |
| ALB | 5130 |  | Tuvalu | 179.033 | -4.683 | -0.0027 | 0.0014 | -1.9241 | 0.0544 | -1.750 | 865 |
| ALB | 5140 |  | Tuvalu | 175.383 | -4.850 | -0.0062 | 0.0026 | -2.4227 | 0.0154 | -3.975 | 243 |
| ALB | 5145 |  | Tuvalu | 176.017 | -4.900 | -0.0133 | 0.0056 | -2.3618 | 0.0182 | -3.659 | 167 |
| YFT | 5192 |  | Tuvalu | 175.167 | -5.350 | -0.0093 | 0.0053 | -1.7641 | 0.0778 | -1.178 | 133 |
| YFT | 5213 |  | Tuvalu | 174.767 | -5.550 | -0.0039 | 0.0016 | -2.4003 | 0.0164 | -3.906 | 558 |
| ALB | 5232 |  | Tuvalu | 181.417 | -5.767 | -0.0023 | 0.0012 | -1.9893 | 0.0467 | -1.997 | 1335 |
| BET | 5277 |  | Tuvalu | 181.117 | -6.333 | -0.0021 | 0.0008 | -2.4926 | 0.0127 | -4.278 | 1611 |
| YFT | 5277 |  | Tuvalu | 181.117 | -6.333 | -0.0017 | 0.0009 | -1.8659 | 0.0621 | -1.519 | 1611 |
| ALB | 5286 |  | Tuvalu | 176.783 | -6.500 | -0.0072 | 0.0049 | -1.4806 | 0.1388 | -0.237 | 174 |
| YFT | 5286 |  | Tuvalu | 176.783 | -6.500 | -0.0084 | 0.0045 | -1.8596 | 0.0630 | -1.529 | 174 |
| ALB | 5307 |  | Tuvalu | 179.433 | -6.917 | -0.0025 | 0.0013 | -2.0274 | 0.0427 | -2.174 | 894 |
| BET | 5367 |  | Tuvalu | 178.517 | -7.567 | -0.0053 | 0.0038 | -1.4022 | 0.1609 | -0.001 | 123 |
| YFT | 5437 |  | Tuvalu | 177.117 | -8.233 | -0.0037 | 0.0017 | -2.1117 | 0.0348 | -2.551 | 406 |
| ALB | 5439 |  | Tuvalu | 174.833 | -8.250 | -0.0022 | 0.0014 | -1.5874 | 0.1125 | -0.573 | 854 |
| BET | 5468 |  | Tuvalu | 178.000 | -8.600 | -0.0061 | 0.0024 | -2.5804 | 0.0099 | -4.788 | 351 |
| YFT | 5468 |  | Tuvalu | 178.000 | -8.600 | -0.0037 | 0.0026 | -1.4199 | 0.1557 | -0.057 | 351 |
| ALB | 5537 |  | Tuvalu | 178.950 | -9.333 | -0.0028 | 0.0016 | -1.7571 | 0.0790 | -1.159 | 486 |
| ALB | 5671 |  | Tuvalu | 179.282 | -10.435 | -0.0051 | 0.0034 | -1.5178 | 0.1292 | -0.366 | 162 |
| YFT | 5913 |  | Tuvalu | 180.433 | -12.450 | -0.0091 | 0.0037 | -2.4456 | 0.0145 | -4.206 | 154 |
| ALB | 6020 |  | Vanuatu | 168.083 | -13.100 | -0.0014 | 0.0008 | -1.7025 | 0.0888 | -0.992 | 918 |
| BET | 6020 |  | Vanuatu | 168.083 | -13.100 | -0.0020 | 0.0009 | -2.2870 | 0.0223 | -3.396 | 918 |
| YFT | 6020 |  | Vanuatu | 168.083 | -13.100 | -0.0039 | 0.0011 | -3.5546 | 0.0004 | -11.017 | 918 |
| BET | 6215 |  | Vanuatu | 165.317 | -14.567 | -0.0040 | 0.0013 | -3.2144 | 0.0013 | -8.520 | 612 |
| BET | 6224 |  | Vanuatu | 165.967 | -14.617 | -0.0036 | 0.0019 | -1.8636 | 0.0625 | -1.546 | 267 |
| BET | 6307 |  | Vanuatu | 165.000 | -15.383 | -0.0039 | 0.0023 | -1.7055 | 0.0882 | -0.961 | 596 |
| ALB | 6308 |  | Vanuatu | 164.050 | -15.400 | -0.0019 | 0.0012 | -1.4949 | 0.1350 | -0.280 | 455 |
| BET | 6308 |  | Vanuatu | 164.050 | -15.400 | -0.0027 | 0.0012 | -2.2114 | 0.0271 | -2.987 | 455 |
| YFT | 6376 |  | Vanuatu | 166.104 | -15.949 | -0.0050 | 0.0022 | -2.2471 | 0.0247 | -3.137 | 283 |
| YFT | 6387 |  | Vanuatu | 166.733 | -16.000 | -0.0044 | 0.0027 | -1.6194 | 0.1054 | -0.676 | 188 |
| BET | 6579 |  | Vanuatu | 167.233 | -17.383 | -0.0042 | 0.0010 | -4.0453 | 0.0001 | -14.682 | 916 |
| BET | 6602 |  | Vanuatu | 169.517 | -17.600 | -0.0040 | 0.0007 | -5.3391 | 0.0000 | -26.897 | 1461 |
| YFT | 6602 |  | Vanuatu | 169.517 | -17.600 | -0.0029 | 0.0009 | -3.0813 | 0.0021 | -7.645 | 1461 |
| BET | 6664 |  | Vanuatu | 167.750 | -18.200 | -0.0021 | 0.0009 | -2.2844 | 0.0224 | -3.339 | 1236 |
| BET | 6683 |  | Vanuatu | 169.800 | -18.417 | -0.0056 | 0.0011 | -5.1619 | 0.0000 | -24.916 | 742 |
| YFT | 6683 |  | Vanuatu | 169.800 | -18.417 | -0.0021 | 0.0014 | -1.4666 | 0.1425 | -0.177 | 742 |
| BET | 6731 |  | Vanuatu | 170.900 | -18.783 | -0.0010 | 0.0005 | -2.1095 | 0.0349 | -2.488 | 2499 |
| YFT | 6731 |  | Vanuatu | 170.900 | -18.783 | -0.0014 | 0.0007 | -1.9827 | 0.0474 | -1.965 | 2499 |
| ALB | 6753 |  | Vanuatu | 170.083 | -18.967 | -0.0097 | 0.0049 | -1.9774 | 0.0480 | -1.951 | 151 |
| BET | 6837 |  | Vanuatu | 170.733 | -20.000 | -0.0015 | 0.0006 | -2.4951 | 0.0126 | -4.297 | 1606 |
| YFT | 6837 |  | Vanuatu | 170.733 | -20.000 | -0.0031 | 0.0009 | -3.3380 | 0.0008 | -9.266 | 1606 |
| YFT | 6927 | Eastern Gemini | Vanuatu | 170.150 | -21.050 | -0.0067 | 0.0016 | -4.2423 | 0.0000 | -16.295 | 751 |
| BET | 5921 | Waterwich | Wallis et Futuna | 183.233 | -12.529 | -0.0077 | 0.0040 | -1.9118 | 0.0560 | -1.795 | 133 |
| YFT | 6100 |  | Wallis et Futuna | 182.750 | -13.767 | -0.0068 | 0.0037 | -1.8075 | 0.0708 | -1.436 | 104 |
| YFT | 6371 |  | Wallis et Futuna | 182.100 | -15.900 | -0.0126 | 0.0058 | -2.1586 | 0.0309 | -2.746 | 164 |
| BET | KL5627 |  |  | 129.633 | 8.050 | -0.0060 | 0.0030 | -1.9797 | 0.0486 | -2.504 | 109 |
| ALB | KL833 |  |  | 203.433 | 39.183 | -0.0042 | 0.0027 | -1.5383 | 0.1251 | -0.598 | 115 |
| YFT | KL833 |  |  | 203.433 | 39.183 | -0.0019 | 0.0012 | -1.5747 | 0.1165 | -0.722 | 115 |
