# Supplementary material for: Tuna Longline Fishing around West and Central Pacific Seamounts
Source: PLoS One. 2010 Dec 29;5(12):e14453. doi: 10.1371/journal.pone.0014453 (PMC3012065; doi:10.1371/journal.pone.0014453)
Supplement: Table S3 — Estimated seamounts catch of tuna for different EEZ in the Pacific Ocean. Prop. of EEZ catch is the proportion of the tuna catch in that EEZ allocated to seamounts. Catch values are in tons. (0.52 MB DOC) [file pone.0014453.s010.doc]

**Table S3**. Estimated seamounts catch of tuna for different EEZ in the Pacific Ocean. Prop. of EEZ catch is the proportion of the tuna catch in that EEZ allocated to seamounts. Catch values are in tons.

|  |  | Yellowfin tuna | | Bigeye tuna | | Albacore | |
| --- | --- | --- | --- | --- | --- | --- | --- |
| EZZ | Year | prop. of EEZ catch | Catch t. | prop. of EEZ catch | Catch t. | prop. of EEZ catch | Catch t. |
| American Samoa | 1990 | 0.767 | 0.6 |  |  | 0.579 | 1.4 |
|  | 1991 |  |  |  |  |  |  |
|  | 1992 | 0.357 | 0.1 |  |  |  |  |
|  | 1993 | 0.039 | 0.1 |  |  |  |  |
|  | 1994 |  |  |  |  |  |  |
|  | 1995 |  |  |  |  |  |  |
|  | 1996 | 0.667 | 0.0 |  |  |  |  |
|  | 1997 |  |  |  |  |  |  |
|  | 1998 | 0.045 | 4.2 | 0.098 | 3.4 | 0.010 | 5.0 |
|  | 1999 | 0.325 | 36.1 | 0.117 | 5.7 | 0.057 | 44.2 |
|  | 2000 | 0.458 | 135.0 | 0.238 | 13.9 | 0.002 | 2.4 |
|  | 2001 | 0.306 | 3.7 | 0.138 | 0.3 | 0.078 | 7.8 |
|  | 2002 | 0.384 | 14.3 | 0.154 | 2.0 | 0.178 | 59.6 |
|  | 2003 | 0.446 | 22.3 | 0.193 | 3.2 | 0.211 | 60.2 |
|  | 2004 | 0.452 | 16.8 | 0.184 | 1.5 | 0.132 | 10.9 |
|  | 2005 | 0.316 | 5.1 | 0.040 | 0.2 | 0.054 | 4.7 |
|  | 2006 | 0.475 | 15.8 | 0.402 | 4.9 | 0.227 | 39.1 |
|  | 2007 | 0.571 | 15.5 | 0.714 | 2.3 | 0.179 | 31.1 |
| Total |  |  | 269.5 |  | 37.5 |  | 266.4 |
| Australia | 1990 | 0.135 | 475.2 | 0.312 | 257.6 | 0.348 | 728.9 |
|  | 1991 | 0.181 | 342.5 | 0.253 | 77.3 | 0.256 | 259.1 |
|  | 1992 | 0.058 | 129.6 | 0.356 | 133.9 | 0.302 | 395.6 |
|  | 1993 | 0.103 | 298.6 | 0.382 | 116.7 | 0.342 | 770.3 |
|  | 1994 | 0.084 | 279.9 | 0.268 | 148.8 | 0.343 | 715.8 |
|  | 1995 | 0.140 | 492.1 | 0.410 | 221.7 | 0.414 | 698.3 |
|  | 1996 | 0.100 | 318.3 | 0.468 | 329.9 | 0.440 | 393.5 |
|  | 1997 | 0.152 | 423.9 | 0.335 | 455.2 | 0.238 | 379.1 |
|  | 1998 | 0.032 | 66.0 | 0.380 | 417.4 | 0.617 | 277.8 |
|  | 1999 | 0.049 | 86.9 | 0.344 | 291.5 | 0.560 | 199.2 |
|  | 2000 | 0.028 | 49.5 | 0.395 | 284.9 | 0.426 | 153.5 |
|  | 2001 | 0.031 | 83.2 | 0.292 | 365.6 | 0.387 | 214.3 |
|  | 2002 | 0.054 | 184.4 | 0.262 | 245.5 | 0.328 | 166.9 |
|  | 2003 | 0.081 | 268.6 | 0.290 | 261.6 | 0.306 | 119.8 |
|  | 2004 | 0.058 | 122.8 | 0.310 | 236.6 | 0.444 | 261.3 |
|  | 2005 | 0.058 | 76.8 | 0.223 | 151.0 | 0.414 | 255.0 |
|  | 2006 | 0.035 | 61.0 | 0.205 | 91.4 | 0.289 | 731.3 |
|  | 2007 | 0.058 | 78.0 | 0.283 | 271.6 | 0.280 | 523.2 |
| Total |  |  | 3837.2 |  | 4358.2 |  | 7242.9 |
| Cook Island | 1990 | 0.285 | 36.2 | 0.179 | 10.4 | 0.022 | 7.0 |
|  | 1991 | 0.191 | 16.5 | 0.189 | 12.7 | 0.134 | 51.9 |
|  | 1992 | 0.075 | 2.0 | 0.038 | 1.0 | 0.003 | 0.3 |
|  | 1993 | 0.250 | 5.2 | 0.247 | 6.7 | 0.014 | 3.4 |
|  | 1994 | 0.217 | 6.3 | 0.096 | 2.6 | 0.032 | 8.0 |
|  | 1995 | 0.012 | 0.2 | 0.000 | 0.0 | 0.007 | 0.2 |
|  | 1996 |  |  |  |  | 0.007 | 0.0 |
|  | 1997 |  |  |  |  |  |  |
|  | 1998 | 0.020 | 1.7 | 0.032 | 1.9 | 0.170 | 95.4 |
|  | 1999 | 0.210 | 1.3 | 0.055 | 0.2 | 0.003 | 0.1 |
|  | 2000 |  |  |  |  |  |  |
|  | 2001 |  |  |  |  |  |  |
|  | 2002 | 0.795 | 37.3 | 0.616 | 37.6 | 0.004 | 2.2 |
|  | 2003 | 0.271 | 56.4 | 0.216 | 53.1 | 0.081 | 118.2 |
|  | 2004 | 0.253 | 128.1 | 0.231 | 91.2 | 0.121 | 225.2 |
|  | 2005 | 0.186 | 71.5 | 0.209 | 43.7 | 0.128 | 289.7 |
|  | 2006 | 0.228 | 45.3 | 0.242 | 35.5 | 0.086 | 167.5 |
|  | 2007 | 0.152 | 25.6 | 0.241 | 36.9 | 0.173 | 352.5 |
| Total |  |  | 433.7 |  | 333.4 |  | 1321.6 |
| Fiji | 1990 | 0.055 | 2.6 | 0.077 | 2.4 | 0.862 | 73.2 |
|  | 1991 | 0.249 | 26.4 | 0.295 | 36.0 | 0.384 | 142.4 |
|  | 1992 | 0.106 | 22.3 | 0.227 | 42.7 | 0.341 | 135.8 |
|  | 1993 | 0.226 | 80.9 | 0.244 | 55.0 | 0.258 | 373.6 |
|  | 1994 | 0.217 | 157.7 | 0.138 | 35.2 | 0.233 | 540.7 |
|  | 1995 | 0.184 | 196.4 | 0.310 | 124.3 | 0.202 | 427.8 |
|  | 1996 | 0.227 | 324.0 | 0.332 | 197.9 | 0.206 | 509.2 |
|  | 1997 | 0.235 | 224.5 | 0.249 | 102.8 | 0.236 | 534.7 |
|  | 1998 | 0.176 | 173.2 | 0.216 | 101.8 | 0.284 | 703.0 |
|  | 1999 | 0.238 | 164.7 | 0.257 | 112.9 | 0.286 | 587.3 |
|  | 2000 | 0.271 | 551.1 | 0.216 | 124.5 | 0.249 | 1188.1 |
|  | 2001 | 0.312 | 589.7 | 0.225 | 136.5 | 0.220 | 1624.5 |
|  | 2002 | 0.287 | 460.7 | 0.194 | 125.6 | 0.241 | 1571.2 |
|  | 2003 | 0.207 | 344.3 | 0.183 | 96.6 | 0.258 | 1102.5 |
|  | 2004 | 0.287 | 631.4 | 0.220 | 117.8 | 0.203 | 1174.0 |
|  | 2005 | 0.283 | 242.4 | 0.230 | 36.4 | 0.269 | 1063.2 |
|  | 2006 | 0.260 | 288.1 | 0.196 | 90.3 | 0.174 | 902.2 |
|  | 2007 | 0.297 | 294.6 | 0.212 | 69.6 | 0.202 | 747.5 |
| Total |  |  | 4775.1 |  | 1608.3 |  | 13401.0 |
| Federated States of Micronesia | 1990 | 0.159 | 1089.3 | 0.122 | 989.1 | 0.076 | 0.9 |
|  | 1991 | 0.181 | 981.3 | 0.153 | 732.4 | 0.073 | 0.8 |
|  | 1992 | 0.131 | 958.3 | 0.182 | 1035.5 | 0.050 | 0.6 |
|  | 1993 | 0.143 | 1022.6 | 0.141 | 1115.6 | 0.132 | 7.2 |
|  | 1994 | 0.159 | 1121.3 | 0.217 | 1924.1 | 0.125 | 7.1 |
|  | 1995 | 0.127 | 1263.1 | 0.126 | 1084.8 | 0.096 | 8.3 |
|  | 1996 | 0.164 | 827.0 | 0.179 | 1076.8 | 0.195 | 15.7 |
|  | 1997 | 0.187 | 878.1 | 0.180 | 894.3 | 0.083 | 6.1 |
|  | 1998 | 0.135 | 636.5 | 0.145 | 699.5 | 0.151 | 11.2 |
|  | 1999 | 0.169 | 562.6 | 0.134 | 897.6 | 0.104 | 11.6 |
|  | 2000 | 0.155 | 590.4 | 0.171 | 991.7 | 0.114 | 14.9 |
|  | 2001 | 0.206 | 451.5 | 0.241 | 1053.3 | 0.184 | 3.7 |
|  | 2002 | 0.217 | 239.8 | 0.256 | 608.2 | 0.093 | 0.2 |
|  | 2003 | 0.218 | 638.7 | 0.224 | 911.1 | 0.294 | 19.4 |
|  | 2004 | 0.177 | 321.1 | 0.202 | 646.2 | 0.191 | 10.8 |
|  | 2005 | 0.112 | 372.5 | 0.163 | 473.4 | 0.228 | 22.9 |
|  | 2006 | 0.102 | 289.7 | 0.151 | 536.9 | 0.277 | 39.1 |
|  | 2007 | 0.146 | 269.2 | 0.205 | 643.3 | 0.086 | 3.8 |
| Total |  |  | 12512.9 |  | 16313.9 |  | 184.3 |
| Kiribati (Gilbert Islands) | 1990 | 0.154 | 90.2 | 0.080 | 49.6 | 0.448 | 1.8 |
|  | 1991 | 0.162 | 77.4 | 0.026 | 9.6 | 0.294 | 6.5 |
|  | 1992 | 0.096 | 227.9 | 0.058 | 89.8 | 0.166 | 2.2 |
|  | 1993 | 0.097 | 124.6 | 0.041 | 39.0 | 0.099 | 0.5 |
|  | 1994 | 0.080 | 180.4 | 0.064 | 139.8 | 0.016 | 0.0 |
|  | 1995 | 0.117 | 208.0 | 0.064 | 97.1 | 0.242 | 23.9 |
|  | 1996 | 0.076 | 274.4 | 0.078 | 44.3 | 0.163 | 7.7 |
|  | 1997 | 0.048 | 26.2 | 0.016 | 20.5 | 0.043 | 2.0 |
|  | 1998 | 0.128 | 25.5 | 0.072 | 39.1 | 0.186 | 3.0 |
|  | 1999 | 0.088 | 41.5 | 0.084 | 55.8 | 0.025 | 0.5 |
|  | 2000 | 0.122 | 251.0 | 0.042 | 34.5 | 0.048 | 0.8 |
|  | 2001 | 0.085 | 151.1 | 0.035 | 113.4 | 0.030 | 2.4 |
|  | 2002 | 0.078 | 134.6 | 0.054 | 155.4 | 0.156 | 12.8 |
|  | 2003 | 0.222 | 204.1 | 0.033 | 39.7 | 0.093 | 8.8 |
|  | 2004 | 0.138 | 118.4 | 0.066 | 81.9 | 0.137 | 26.9 |
|  | 2005 | 0.216 | 77.3 | 0.086 | 43.8 | 0.108 | 6.9 |
|  | 2006 | 0.158 | 104.3 | 0.044 | 41.8 | 0.116 | 3.3 |
|  | 2007 | 0.165 | 153.2 | 0.061 | 67.1 | 0.285 | 6.0 |
| Total |  |  | 2470.1 |  | 1162.1 |  | 116.2 |
| South Pacific RFMO High seas | 1990 | 0.180 | 5617.1 | 0.122 | 5997.5 | 0.098 | 1938.1 |
|  | 1991 | 0.201 | 4412.4 | 0.210 | 7853.0 | 0.106 | 2528.0 |
|  | 1992 | 0.194 | 5602.1 | 0.195 | 9427.0 | 0.095 | 3099.1 |
|  | 1993 | 0.122 | 3236.8 | 0.114 | 4332.6 | 0.093 | 2772.0 |
|  | 1994 | 0.145 | 4613.1 | 0.101 | 4344.1 | 0.129 | 3999.1 |
|  | 1995 | 0.139 | 4387.4 | 0.120 | 4404.1 | 0.113 | 3405.5 |
|  | 1996 | 0.131 | 4260.3 | 0.085 | 2574.9 | 0.092 | 2821.8 |
|  | 1997 | 0.080 | 2599.8 | 0.113 | 4434.2 | 0.066 | 2102.0 |
|  | 1998 | 0.083 | 2628.8 | 0.135 | 7410.6 | 0.093 | 3507.9 |
|  | 1999 | 0.105 | 2405.2 | 0.119 | 4572.4 | 0.066 | 2110.7 |
|  | 2000 | 0.087 | 2925.9 | 0.087 | 3512.3 | 0.054 | 1550.4 |
|  | 2001 | 0.060 | 1968.4 | 0.100 | 4323.5 | 0.137 | 5097.9 |
|  | 2002 | 0.158 | 6016.1 | 0.094 | 5628.6 | 0.052 | 2092.2 |
|  | 2003 | 0.135 | 4596.0 | 0.084 | 4152.0 | 0.060 | 2094.5 |
|  | 2004 | 0.162 | 5486.4 | 0.090 | 5510.7 | 0.028 | 992.6 |
|  | 2005 | 0.160 | 4965.7 | 0.082 | 3743.7 | 0.019 | 683.1 |
|  | 2006 | 0.168 | 4412.2 | 0.121 | 4971.9 | 0.039 | 1081.7 |
|  | 2007 | 0.167 | 3531.6 | 0.081 | 3256.7 | 0.027 | 674.0 |
| Total |  |  | 73665.4 |  | 90449.7 |  | 42550.5 |
| Kiribati (Line Islands) | 1990 | 0.070 | 81.5 | 0.081 | 112.5 | 0.126 | 9.7 |
|  | 1991 | 0.016 | 10.5 | 0.155 | 122.4 | 0.318 | 32.4 |
|  | 1992 | 0.077 | 28.1 | 0.155 | 98.0 | 0.332 | 16.4 |
|  | 1993 | 0.036 | 33.0 | 0.089 | 135.3 | 0.329 | 21.1 |
|  | 1994 | 0.022 | 56.7 | 0.131 | 584.9 | 0.454 | 201.5 |
|  | 1995 | 0.032 | 35.5 | 0.141 | 287.5 | 0.338 | 75.8 |
|  | 1996 | 0.027 | 47.0 | 0.094 | 162.5 | 0.262 | 20.5 |
|  | 1997 | 0.048 | 98.9 | 0.142 | 339.5 | 0.272 | 78.8 |
|  | 1998 | 0.063 | 100.0 | 0.148 | 373.3 | 0.381 | 297.6 |
|  | 1999 | 0.040 | 73.0 | 0.125 | 739.5 | 0.273 | 25.7 |
|  | 2000 | 0.076 | 196.5 | 0.089 | 485.8 | 0.213 | 39.1 |
|  | 2001 | 0.056 | 160.2 | 0.131 | 508.0 | 0.272 | 143.1 |
|  | 2002 | 0.115 | 205.8 | 0.137 | 540.9 | 0.165 | 165.3 |
|  | 2003 | 0.054 | 76.0 | 0.155 | 265.8 | 0.209 | 170.2 |
|  | 2004 | 0.035 | 89.4 | 0.157 | 562.0 | 0.302 | 103.5 |
|  | 2005 | 0.042 | 25.3 | 0.147 | 193.8 | 0.265 | 75.5 |
|  | 2006 | 0.059 | 69.1 | 0.150 | 321.0 | 0.209 | 87.0 |
|  | 2007 | 0.060 | 23.1 | 0.097 | 129.3 | 0.144 | 81.0 |
| Total |  |  | 1409.6 |  | 5961.9 |  | 1644.1 |
| Marshall Island | 1990 | 0.059 | 63.5 | 0.191 | 356.7 | 0.500 | 6.5 |
|  | 1991 | 0.049 | 66.4 | 0.124 | 209.2 | 0.277 | 9.3 |
|  | 1992 | 0.023 | 67.1 | 0.063 | 204.3 | 0.369 | 4.7 |
|  | 1993 | 0.054 | 81.0 | 0.132 | 284.2 | 0.395 | 7.1 |
|  | 1994 | 0.042 | 112.1 | 0.102 | 350.7 | 0.347 | 18.2 |
|  | 1995 | 0.017 | 47.7 | 0.092 | 190.2 | 0.485 | 7.9 |
|  | 1996 | 0.046 | 92.0 | 0.115 | 143.4 | 0.438 | 21.1 |
|  | 1997 | 0.059 | 45.3 | 0.146 | 149.2 | 0.416 | 6.8 |
|  | 1998 | 0.053 | 36.0 | 0.075 | 98.6 | 0.227 | 7.6 |
|  | 1999 | 0.027 | 45.1 | 0.074 | 195.9 | 0.306 | 59.1 |
|  | 2000 | 0.075 | 87.5 | 0.168 | 142.6 | 0.534 | 27.2 |
|  | 2001 | 0.144 | 181.8 | 0.077 | 212.3 | 0.663 | 29.1 |
|  | 2002 | 0.075 | 35.0 | 0.089 | 159.1 | 0.514 | 6.7 |
|  | 2003 | 0.030 | 28.0 | 0.117 | 270.2 | 0.480 | 8.2 |
|  | 2004 | 0.090 | 50.3 | 0.107 | 189.1 | 0.538 | 30.1 |
|  | 2005 | 0.040 | 30.8 | 0.107 | 145.1 | 0.314 | 17.7 |
|  | 2006 | 0.061 | 74.8 | 0.088 | 136.0 | 0.168 | 7.6 |
|  | 2007 | 0.107 | 102.9 | 0.173 | 450.5 | 0.091 | 0.9 |
| Total |  |  | 1247.3 |  | 3887.2 |  | 275.7 |
| New Caledonia | 1990 | 0.273 | 281.9 | 0.063 | 6.8 | 0.189 | 269.2 |
|  | 1991 | 0.253 | 143.4 | 0.021 | 1.0 | 0.079 | 75.8 |
|  | 1992 | 0.231 | 101.6 | 0.088 | 2.0 | 0.315 | 210.1 |
|  | 1993 | 0.081 | 32.1 | 0.077 | 7.3 | 0.181 | 122.6 |
|  | 1994 | 0.146 | 83.6 | 0.152 | 15.8 | 0.103 | 129.8 |
|  | 1995 | 0.207 | 226.6 | 0.134 | 19.4 | 0.107 | 79.4 |
|  | 1996 | 0.272 | 111.2 | 0.042 | 6.7 | 0.059 | 20.7 |
|  | 1997 | 0.399 | 188.3 | 0.134 | 27.1 | 0.133 | 24.6 |
|  | 1998 | 0.179 | 32.2 | 0.284 | 134.3 | 0.173 | 141.5 |
|  | 1999 | 0.127 | 46.3 | 0.358 | 191.9 | 0.200 | 130.0 |
|  | 2000 | 0.236 | 58.5 | 0.324 | 163.5 | 0.221 | 194.4 |
|  | 2001 | 0.336 | 191.3 | 0.187 | 23.4 | 0.222 | 225.9 |
|  | 2002 | 0.294 | 167.0 | 0.232 | 42.8 | 0.184 | 215.3 |
|  | 2003 | 0.178 | 134.3 | 0.269 | 38.1 | 0.117 | 127.6 |
|  | 2004 | 0.207 | 126.3 | 0.214 | 18.4 | 0.108 | 147.0 |
|  | 2005 | 0.183 | 82.0 | 0.317 | 23.9 | 0.072 | 113.3 |
|  | 2006 | 0.180 | 74.5 | 0.322 | 11.3 | 0.095 | 127.5 |
|  | 2007 | 0.393 | 154.6 | 0.277 | 14.6 | 0.096 | 125.6 |
| Total |  |  | 2236.0 |  | 748.2 |  | 2480.4 |
| Norfolk Island | 1990 | 0.136 | 15.6 | 0.041 | 1.8 | 0.185 | 11.4 |
|  | 1991 | 0.073 | 0.1 | 0.037 | 0.1 |  |  |
|  | 1992 |  |  |  |  | 0.040 | 1.5 |
|  | 1993 |  |  |  |  | 0.101 | 2.0 |
|  | 1994 |  |  |  |  |  |  |
|  | 1995 |  |  |  |  |  |  |
|  | 1996 |  |  |  |  |  |  |
|  | 1997 | 0.002 | 0.5 | 0.001 | 0.0 | 0.208 | 13.5 |
|  | 1998 |  |  |  |  |  |  |
|  | 1999 |  |  |  |  |  |  |
|  | 2000 |  |  |  |  | 0.000 | 0.0 |
|  | 2001 | 0.047 | 0.1 | 0.048 | 0.1 | 0.067 | 1.0 |
|  | 2002 | 0.045 | 0.2 | 0.096 | 0.3 |  |  |
|  | 2003 | 0.097 | 2.8 | 0.076 | 0.6 | 0.394 | 1.0 |
|  | 2004 |  |  |  |  | 0.047 | 0.4 |
|  | 2005 |  |  |  |  |  |  |
|  | 2006 |  |  |  |  |  |  |
|  | 2007 |  |  |  |  |  |  |
| Total |  |  | 19.4 |  | 2.9 |  | 30.7 |
| Niue | 1990 |  |  |  |  |  |  |
|  | 1991 |  |  |  |  |  |  |
|  | 1992 |  |  |  |  |  |  |
|  | 1993 | 0.367 | 4.0 | 0.200 | 0.4 | 0.046 | 5.6 |
|  | 1994 | 0.388 | 6.9 | 0.526 | 4.6 | 0.025 | 6.6 |
|  | 1995 | 0.468 | 2.9 | 0.133 | 0.2 |  |  |
|  | 1996 |  |  |  |  |  |  |
|  | 1997 | 0.200 | 0.1 | 0.000 | 0.0 |  |  |
|  | 1998 |  |  |  |  | 0.032 | 0.1 |
|  | 1999 |  |  |  |  |  |  |
|  | 2000 | 0.000 | 0.0 | 0.250 | 0.0 |  |  |
|  | 2001 |  |  |  |  |  |  |
|  | 2002 |  |  |  |  |  |  |
|  | 2003 |  |  |  |  |  |  |
|  | 2004 |  |  |  |  |  |  |
|  | 2005 | 0.641 | 21.8 | 0.606 | 6.1 | 0.035 | 1.9 |
|  | 2006 | 0.601 | 29.7 | 0.148 | 3.9 | 0.002 | 0.5 |
|  | 2007 | 0.555 | 16.0 | 0.081 | 1.2 |  |  |
| Total |  |  | 81.5 |  | 16.3 |  | 14.7 |
| New Zealand | 1990 | 0.031 | 1.0 | 0.087 | 32.6 | 0.048 | 28.2 |
|  | 1991 | 0.303 | 2.4 | 0.241 | 32.4 | 0.020 | 9.3 |
|  | 1992 | 0.405 | 5.3 | 0.453 | 52.1 | 0.019 | 9.9 |
|  | 1993 | 0.097 | 1.1 | 0.049 | 3.2 | 0.113 | 46.5 |
|  | 1994 | 0.222 | 7.1 | 0.044 | 3.0 | 0.061 | 38.9 |
|  | 1995 | 0.219 | 20.5 | 0.081 | 4.8 | 0.119 | 96.0 |
|  | 1996 | 0.320 | 42.3 | 0.037 | 3.1 | 0.102 | 106.0 |
|  | 1997 | 0.188 | 22.7 | 0.012 | 1.7 | 0.116 | 98.5 |
|  | 1998 | 0.130 | 15.1 | 0.039 | 15.0 | 0.189 | 396.0 |
|  | 1999 | 0.103 | 15.9 | 0.045 | 18.9 | 0.161 | 342.5 |
|  | 2000 | 0.063 | 5.9 | 0.110 | 45.1 | 0.070 | 98.0 |
|  | 2001 | 0.155 | 20.3 | 0.168 | 79.8 | 0.072 | 190.5 |
|  | 2002 | 0.088 | 2.5 | 0.107 | 21.4 | 0.070 | 179.3 |
|  | 2003 | 0.061 | 2.9 | 0.161 | 33.3 | 0.054 | 168.0 |
|  | 2004 | 0.105 | 3.2 | 0.150 | 26.8 | 0.116 | 148.3 |
|  | 2005 | 0.104 | 3.5 | 0.266 | 47.5 | 0.102 | 65.9 |
|  | 2006 | 0.108 | 0.4 | 0.094 | 16.7 | 0.146 | 76.8 |
|  | 2007 | 0.114 | 1.6 | 0.117 | 24.2 | 0.081 | 22.7 |
| Total |  |  | 173.6 |  | 461.6 |  | 2121.6 |
| French Polynesia | 1990 | 0.349 | 237.9 | 0.237 | 250.8 | 0.101 | 42.4 |
|  | 1991 | 0.351 | 187.3 | 0.258 | 271.8 | 0.155 | 41.7 |
|  | 1992 | 0.453 | 159.7 | 0.104 | 56.6 | 0.221 | 61.8 |
|  | 1993 | 0.590 | 300.5 | 0.163 | 79.0 | 0.224 | 170.1 |
|  | 1994 | 0.402 | 181.7 | 0.141 | 100.6 | 0.273 | 259.1 |
|  | 1995 | 0.418 | 176.4 | 0.077 | 64.8 | 0.207 | 180.6 |
|  | 1996 | 0.435 | 369.5 | 0.168 | 123.4 | 0.187 | 293.8 |
|  | 1997 | 0.432 | 265.8 | 0.178 | 145.9 | 0.160 | 407.5 |
|  | 1998 | 0.300 | 226.0 | 0.195 | 178.0 | 0.104 | 341.7 |
|  | 1999 | 0.441 | 512.8 | 0.100 | 128.0 | 0.184 | 475.0 |
|  | 2000 | 0.286 | 441.3 | 0.149 | 241.3 | 0.215 | 744.8 |
|  | 2001 | 0.343 | 322.3 | 0.282 | 204.7 | 0.191 | 815.6 |
|  | 2002 | 0.281 | 129.6 | 0.282 | 164.6 | 0.144 | 603.0 |
|  | 2003 | 0.256 | 147.4 | 0.245 | 101.0 | 0.161 | 584.7 |
|  | 2004 | 0.282 | 300.7 | 0.303 | 153.8 | 0.153 | 334.8 |
|  | 2005 | 0.436 | 317.7 | 0.367 | 187.3 | 0.119 | 248.4 |
|  | 2006 | 0.286 | 178.3 | 0.275 | 120.2 | 0.132 | 349.8 |
|  | 2007 | 0.372 | 169.9 | 0.415 | 174.8 | 0.080 | 285.6 |
| Total |  |  | 4625.0 |  | 2746.6 |  | 6240.4 |
| Papua New Guinea | 1990 | 0.360 | 120.0 | 0.225 | 11.5 | 0.455 | 172.1 |
|  | 1991 | 0.457 | 11.7 | 0.354 | 9.7 | 0.323 | 0.8 |
|  | 1992 | 0.086 | 7.4 | 0.308 | 41.8 |  |  |
|  | 1993 | 0.324 | 57.1 | 0.518 | 107.5 | 0.422 | 0.0 |
|  | 1994 | 0.401 | 181.0 | 0.219 | 60.7 | 0.389 | 0.0 |
|  | 1995 | 0.368 | 110.7 | 0.389 | 53.2 | 0.421 | 2.6 |
|  | 1996 | 0.351 | 66.1 | 0.402 | 6.5 | 0.392 | 14.9 |
|  | 1997 | 0.425 | 165.7 | 0.398 | 23.5 | 0.346 | 46.1 |
|  | 1998 | 0.385 | 185.6 | 0.613 | 25.8 | 0.209 | 21.7 |
|  | 1999 | 0.868 | 425.5 | 0.542 | 33.4 | 0.739 | 95.1 |
|  | 2000 | 0.316 | 269.0 | 0.509 | 99.1 | 0.534 | 84.8 |
|  | 2001 | 0.501 | 909.0 | 0.453 | 109.5 | 0.643 | 79.7 |
|  | 2002 | 0.480 | 840.4 | 0.278 | 88.9 | 0.536 | 75.9 |
|  | 2003 | 0.569 | 996.7 | 0.224 | 87.3 | 0.503 | 429.7 |
|  | 2004 | 0.573 | 1331.5 | 0.318 | 127.4 | 0.201 | 338.0 |
|  | 2005 | 0.677 | 840.9 | 0.414 | 98.3 | 0.205 | 462.6 |
|  | 2006 | 0.647 | 1450.3 | 0.450 | 112.7 | 0.351 | 655.2 |
|  | 2007 | 0.567 | 1078.4 | 0.210 | 31.8 | 0.096 | 189.2 |
| Total |  |  | 9047.0 |  | 1128.5 |  | 2668.3 |
| Palau | 1990 | 0.033 | 37.8 | 0.008 | 10.1 | 0.000 | 0.0 |
|  | 1991 | 0.013 | 11.1 | 0.002 | 2.0 | 0.000 | 0.0 |
|  | 1992 | 0.002 | 2.7 | 0.015 | 27.8 | 0.000 | 0.0 |
|  | 1993 | 0.017 | 18.9 |  |  | 0.000 | 0.0 |
|  | 1994 | 0.029 | 16.3 | 0.004 | 3.6 | 0.000 | 0.0 |
|  | 1995 | 0.010 | 5.4 | 0.001 | 0.5 | 0.000 | 0.0 |
|  | 1996 | 0.003 | 1.3 |  |  | 0.000 | 0.0 |
|  | 1997 | 0.000 | 0.0 |  |  | 0.000 | 0.0 |
|  | 1998 | 0.008 | 2.7 | 0.001 | 0.3 | 0.000 | 0.0 |
|  | 1999 | 0.015 | 8.2 |  |  | 0.312 | 7.9 |
|  | 2000 | 0.028 | 21.4 | 0.000 | 0.0 | 0.108 | 1.2 |
|  | 2001 | 0.013 | 12.3 | 0.001 | 0.4 | 0.089 | 0.9 |
|  | 2002 | 0.023 | 10.5 | 0.001 | 0.4 | 0.071 | 0.0 |
|  | 2003 | 0.025 | 17.8 | 0.007 | 3.5 | 0.000 | 0.0 |
|  | 2004 | 0.012 | 12.2 | 0.031 | 13.9 | 0.000 | 0.0 |
|  | 2005 | 0.014 | 18.5 | 0.010 | 15.1 | 0.014 | 2.1 |
|  | 2006 | 0.010 | 15.8 | 0.015 | 44.3 | 0.000 | 0.0 |
|  | 2007 | 0.001 | 1.9 | 0.028 | 45.4 | 0.000 | 0.0 |
| Total |  |  | 214.7 |  | 167.2 |  | 12.1 |
| Kiribati (Phoenix Islands) | 1990 | 0.162 | 73.2 | 0.101 | 32.2 | 0.090 | 3.0 |
|  | 1991 | 0.133 | 61.3 | 0.069 | 22.9 | 0.112 | 5.0 |
|  | 1992 | 0.198 | 125.5 | 0.085 | 56.1 | 0.080 | 6.3 |
|  | 1993 | 0.227 | 176.6 | 0.103 | 68.6 | 0.160 | 6.2 |
|  | 1994 | 0.233 | 232.0 | 0.163 | 231.4 | 0.119 | 19.9 |
|  | 1995 | 0.206 | 128.1 | 0.043 | 46.7 | 0.135 | 46.4 |
|  | 1996 | 0.266 | 234.7 | 0.084 | 34.9 | 0.101 | 11.3 |
|  | 1997 | 0.129 | 96.0 | 0.118 | 86.0 | 0.150 | 27.7 |
|  | 1998 | 0.213 | 116.6 | 0.139 | 114.9 | 0.165 | 25.9 |
|  | 1999 | 0.301 | 170.1 | 0.145 | 75.0 | 0.159 | 7.9 |
|  | 2000 | 0.235 | 212.0 | 0.085 | 59.3 | 0.154 | 11.8 |
|  | 2001 | 0.082 | 27.9 | 0.034 | 15.8 | 0.085 | 14.9 |
|  | 2002 | 0.167 | 103.4 | 0.087 | 79.2 | 0.099 | 15.8 |
|  | 2003 | 0.255 | 118.6 | 0.049 | 30.0 | 0.129 | 9.1 |
|  | 2004 | 0.194 | 262.4 | 0.114 | 147.2 | 0.098 | 30.4 |
|  | 2005 | 0.233 | 132.5 | 0.104 | 98.7 | 0.086 | 8.0 |
|  | 2006 | 0.215 | 87.7 | 0.099 | 58.6 | 0.107 | 4.5 |
|  | 2007 | 0.282 | 375.5 | 0.071 | 112.1 | 0.143 | 22.5 |
| Total |  |  | 2734.1 |  | 1369.9 |  | 276.6 |
| Palmyra | 1990 |  |  | 0.176 | 119.4 | 0.011 | 3.8 |
|  | 1991 |  |  | 0.320 | 462.8 | 0.016 | 3.9 |
|  | 1992 |  |  | 0.189 | 102.1 | 0.082 | 33.3 |
|  | 1993 |  |  | 0.135 | 73.7 | 0.020 | 12.5 |
|  | 1994 |  |  | 0.244 | 85.6 | 0.044 | 55.2 |
|  | 1995 |  |  | 0.246 | 441.9 | 0.056 | 94.9 |
|  | 1996 |  |  | 0.224 | 270.4 | 0.053 | 78.0 |
|  | 1997 |  |  | 0.136 | 220.5 | 0.078 | 46.9 |
|  | 1998 |  |  | 0.199 | 209.9 | 0.037 | 42.9 |
|  | 1999 |  |  | 0.104 | 88.3 | 0.131 | 42.9 |
|  | 2000 |  |  | 0.211 | 100.7 | 0.089 | 29.2 |
|  | 2001 |  |  | 0.245 | 58.9 | 0.076 | 14.1 |
|  | 2002 |  |  | 0.231 | 171.3 | 0.051 | 53.7 |
|  | 2003 |  |  | 0.334 | 360.8 | 0.051 | 47.5 |
|  | 2004 |  |  | 0.129 | 97.4 | 0.020 | 45.9 |
|  | 2005 |  |  | 0.105 | 56.7 | 0.026 | 82.2 |
|  | 2006 |  |  | 0.162 | 186.3 | 0.029 | 197.7 |
|  | 2007 |  |  | 0.150 | 163.6 | 0.055 | 268.2 |
| Total |  |  |  |  | 3270.6 |  | 1152.9 |
| Solomon Islands | 1990 | 0.085 | 269.1 | 0.096 | 0.5 | 0.607 | 2.3 |
|  | 1991 | 0.116 | 332.7 | 0.024 | 0.3 |  |  |
|  | 1992 | 0.148 | 217.0 | 0.010 | 0.2 | 0.259 | 2.1 |
|  | 1993 | 0.109 | 206.4 |  |  | 0.000 | 0.0 |
|  | 1994 | 0.076 | 121.3 |  |  |  |  |
|  | 1995 | 0.136 | 602.4 | 0.147 | 0.9 |  |  |
|  | 1996 | 0.145 | 385.3 |  |  |  |  |
|  | 1997 | 0.179 | 426.4 |  |  |  |  |
|  | 1998 | 0.117 | 133.6 |  |  |  |  |
|  | 1999 | 0.096 | 89.7 |  |  |  |  |
|  | 2000 | 0.126 | 105.0 |  |  |  |  |
|  | 2001 | 0.192 | 46.4 |  |  |  |  |
|  | 2002 | 0.114 | 173.2 |  |  | 0.364 | 1.3 |
|  | 2003 | 0.174 | 283.3 |  |  | 0.325 | 0.1 |
|  | 2004 | 0.312 | 537.1 |  |  | 0.689 | 0.3 |
|  | 2005 | 0.254 | 375.7 | 0.000 | 0.0 | 0.568 | 4.3 |
|  | 2006 | 0.138 | 510.0 |  |  |  |  |
|  | 2007 | 0.078 | 359.4 |  |  | 0.500 | 4.0 |
| Total |  |  | 5174.0 |  | 1.8 |  | 14.4 |
| Tokelau | 1990 |  |  | 0.231 | 2.3 | 0.030 | 2.9 |
|  | 1991 |  |  | 0.130 | 0.8 | 0.028 | 3.5 |
|  | 1992 |  |  | 0.245 | 0.9 | 0.021 | 2.9 |
|  | 1993 |  |  | 0.333 | 11.5 | 0.020 | 5.7 |
|  | 1994 |  |  | 0.255 | 4.6 | 0.013 | 4.2 |
|  | 1995 |  |  | 1.000 | 23.2 |  |  |
|  | 1996 |  |  | 0.733 | 44.4 | 0.023 | 10.1 |
|  | 1997 |  |  | 0.497 | 33.5 | 0.101 | 50.3 |
|  | 1998 |  |  | 0.264 | 40.7 | 0.243 | 456.8 |
|  | 1999 |  |  | 0.405 | 59.9 | 0.094 | 98.0 |
|  | 2000 |  |  | 0.411 | 48.3 | 0.045 | 39.4 |
|  | 2001 |  |  | 0.258 | 47.3 | 0.033 | 35.5 |
|  | 2002 | 0.679 | 0.7 | 0.374 | 60.0 | 0.076 | 71.5 |
|  | 2003 | 0.341 | 0.6 | 0.393 | 38.5 | 0.105 | 77.6 |
|  | 2004 | 0.561 | 0.7 | 0.426 | 18.3 | 0.024 | 4.8 |
|  | 2005 | 0.000 | 0.0 | 0.542 | 66.2 | 0.049 | 13.2 |
|  | 2006 |  |  | 0.444 | 52.0 | 0.043 | 20.2 |
|  | 2007 |  |  | 0.325 | 42.1 | 0.042 | 17.1 |
| Total |  |  | 2.0 |  | 594.6 |  | 913.6 |
| Tonga | 1990 | 0.248 | 6.2 | 0.147 | 15.0 | 0.290 | 72.4 |
|  | 1991 | 0.228 | 4.3 | 0.115 | 4.2 | 0.125 | 8.5 |
|  | 1992 | 0.360 | 6.0 | 0.172 | 4.2 | 0.118 | 1.9 |
|  | 1993 | 0.480 | 31.0 | 0.019 | 0.3 |  |  |
|  | 1994 | 0.508 | 22.4 |  |  |  |  |
|  | 1995 |  |  | 0.031 | 0.6 |  |  |
|  | 1996 | 0.604 | 54.3 |  |  |  |  |
|  | 1997 | 0.548 | 58.4 |  |  | 0.719 | 1.3 |
|  | 1998 | 0.380 | 125.1 | 0.070 | 15.5 | 0.279 | 49.3 |
|  | 1999 | 0.546 | 119.5 | 0.028 | 1.6 | 0.247 | 20.0 |
|  | 2000 | 0.533 | 97.0 | 0.179 | 55.7 | 0.280 | 68.3 |
|  | 2001 | 0.426 | 105.8 | 0.096 | 30.4 | 0.323 | 40.9 |
|  | 2002 | 0.555 | 125.2 | 0.075 | 31.6 | 0.232 | 48.0 |
|  | 2003 | 0.521 | 165.8 | 0.009 | 1.0 | 0.463 | 32.7 |
|  | 2004 | 0.425 | 75.1 | 0.041 | 9.7 | 0.189 | 49.0 |
|  | 2005 | 0.501 | 106.7 | 0.088 | 28.6 | 0.275 | 69.4 |
|  | 2006 | 0.559 | 127.1 | 0.013 | 0.3 |  |  |
|  | 2007 | 0.588 | 200.1 | 0.128 | 38.2 | 0.168 | 33.3 |
| Total |  |  | 1430.2 |  | 236.8 |  | 495.1 |
| Tuvalu | 1990 | 0.186 | 76.1 | 0.626 | 9.5 | 0.290 | 93.5 |
|  | 1991 | 0.145 | 10.6 | 0.792 | 4.2 | 0.157 | 17.5 |
|  | 1992 | 0.562 | 20.1 | 0.562 | 11.6 | 0.108 | 53.1 |
|  | 1993 | 0.390 | 13.0 | 0.591 | 25.6 | 0.039 | 80.7 |
|  | 1994 | 0.427 | 2.7 | 0.534 | 10.6 | 0.132 | 122.3 |
|  | 1995 | 0.402 | 3.7 | 0.566 | 16.0 | 0.165 | 150.8 |
|  | 1996 | 0.225 | 6.5 | 0.699 | 93.9 | 0.228 | 218.1 |
|  | 1997 |  |  | 0.669 | 11.4 | 0.088 | 55.7 |
|  | 1998 | 0.145 | 30.1 | 0.485 | 7.3 | 0.139 | 81.6 |
|  | 1999 | 0.157 | 7.3 | 0.551 | 20.4 |  |  |
|  | 2000 | 0.219 | 144.9 | 0.583 | 62.1 | 0.026 | 74.9 |
|  | 2001 | 0.337 | 76.0 | 0.637 | 130.7 | 0.049 | 135.8 |
|  | 2002 | 0.248 | 120.5 | 0.578 | 116.9 | 0.101 | 262.2 |
|  | 2003 | 0.248 | 24.0 | 0.608 | 223.8 | 0.032 | 88.7 |
|  | 2004 | 0.229 | 109.9 | 0.398 | 96.4 | 0.082 | 302.7 |
|  | 2005 | 0.161 | 69.2 | 0.553 | 120.2 | 0.110 | 634.7 |
|  | 2006 | 0.081 | 1.3 | 0.620 | 210.5 | 0.180 | 1489.4 |
|  | 2007 | 0.208 | 85.2 | 0.580 | 151.2 | 0.146 | 849.8 |
| Total |  |  | 801.1 |  | 1322.0 |  | 4711.5 |
| Vanuatu | 1990 | 0.435 | 52.8 |  |  | 0.097 | 2.7 |
|  | 1991 | 0.188 | 6.4 |  |  | 0.101 | 7.2 |
|  | 1992 | 0.443 | 39.7 |  |  |  |  |
|  | 1993 | 0.412 | 53.2 |  |  | 0.112 | 45.0 |
|  | 1994 | 0.320 | 37.9 |  |  | 0.164 | 134.1 |
|  | 1995 | 0.180 | 20.1 |  |  |  |  |
|  | 1996 | 0.247 | 101.8 |  |  |  |  |
|  | 1997 | 0.513 | 52.7 | 0.047 | 0.8 |  |  |
|  | 1998 | 0.508 | 29.7 |  |  | 0.016 | 46.1 |
|  | 1999 | 0.403 | 16.2 |  |  | 0.005 | 12.8 |
|  | 2000 | 0.411 | 236.2 |  |  | 0.005 | 10.8 |
|  | 2001 | 0.357 | 263.4 |  |  |  |  |
|  | 2002 | 0.361 | 321.2 |  |  | 0.017 | 64.6 |
|  | 2003 | 0.270 | 374.5 |  |  | 0.102 | 147.2 |
|  | 2004 | 0.332 | 294.3 |  |  | 0.065 | 73.9 |
|  | 2005 | 0.357 | 405.8 | 0.120 | 0.3 | 0.115 | 132.2 |
|  | 2006 | 0.446 | 615.8 |  |  | 0.183 | 347.1 |
|  | 2007 | 0.401 | 365.3 |  |  | 0.143 | 416.3 |
| Total |  |  | 3287.1 |  | 1.1 | 1.125 | 1440.1 |
| Wallis et Futuna | 1990 |  |  | 0.063 | 0.1 |  |  |
|  | 1991 |  |  | 0.156 | 1.1 |  |  |
|  | 1992 |  |  |  |  |  |  |
|  | 1993 |  |  | 0.114 | 0.9 |  |  |
|  | 1994 |  |  | 0.160 | 2.9 |  |  |
|  | 1995 |  |  |  |  |  |  |
|  | 1996 | 0.016 | 0.6 |  |  |  |  |
|  | 1997 | 0.024 | 0.3 |  |  |  |  |
|  | 1998 | 0.271 | 4.4 | 0.023 | 5.3 |  |  |
|  | 1999 |  |  | 0.002 | 0.5 |  |  |
|  | 2000 | 0.040 | 4.9 | 0.000 | 0.0 |  |  |
|  | 2001 | 0.070 | 0.9 |  |  |  |  |
|  | 2002 |  |  |  |  |  |  |
|  | 2003 |  |  |  |  |  |  |
|  | 2004 |  |  |  |  |  |  |
|  | 2005 | 0.278 | 5.1 | 0.080 | 4.6 |  |  |
|  | 2006 |  |  |  |  |  |  |
|  | 2007 |  |  | 0.118 | 11.4 |  |  |
| Total |  |  | 16.308 |  | 26.809 |  |  |
